# Metastasis of triple negative breast cancer is regulated by a targetable miR-342-E2F network

**DOI:** 10.1038/s44321-026-00496-4

**Published:** 2026-08-21

**Authors:** Victoria K Arnet, Cameron N Johnstone, Richard P Redvers, Caroline A Chambers, Katherine A Pillman, John Toubia, Rachael Lumb, B Kate Dredge, Andrew G Bert, Emily Hackett-Jones, Pannapa Pinweha, Julie M Bracken, Shruti Deshpande, Zoe K Price, Michael Ortiz, Kaitlin G Scheer, Jasleen Rajpal, Ashleigh B Geiger, Suraya Roslan, Xiaochun Li, Sandra O’Toole, Traude H Beilharz, Cameron P Bracken, Yeesim Khew-Goodall, Gregory J Goodall, Robin L Anderson, Philip A Gregory

**Affiliations:** 1 Centre for Cancer Biology, Adelaide University and SA Pathology, Adelaide, SA Australia; 2 Olivia Newton-John Cancer Research Institute, Heidelberg, VIC Australia; 3 South Australian immunoGENomics Cancer Institute, Adelaide University, Adelaide, SA Australia; 4 Garvan Institute of Medical Research, Sydney, NSW Australia; 5 Department of Biochemistry and Molecular Biology, Monash University, Clayton, VIC Australia; 6 School of Cancer Medicine, La Trobe University, Bundoora, VIC Australia; 7 Department of Clinical Pathology, The University of Melbourne, Parkville, VIC Australia

**Keywords:** Cancer, RNA Biology

## Abstract

Triple-negative breast cancer (TNBC) has a high incidence of metastasis and limited therapeutic options. Here, we identify a multimodal and targetable miR-342-E2F network that regulates metastatic outgrowth in TNBC. Through integrating clinical and experimental datasets, we uncover miR-342 as a suppressor of TNBC metastasis. Temporal re-expression of miR-342 significantly inhibited metastatic progression in both immunocompetent and xenograft TNBC models by specifically limiting the outgrowth of disseminated tumour cells. Using multi-omics profiling, we identified the global complement of miR-342 targets, demonstrating that it directly suppresses transcriptional and post-transcriptional networks that converge to dampen E2F signalling. Single-cell analysis of matched primary and metastatic patient-derived TNBC samples reveals activation of E2F signalling in metastasis that corresponds with reduced miR-342 host gene *EVL* expression. Furthermore, pharmacologic inhibition of this pathway with the CDK4/6 inhibitor palbociclib specifically reduces metastatic outgrowth of pre-established TNBC lesions. Our findings reveal that TNBCs with low miR-342/high E2F signalling have increased metastatic competency and may be amenable to CDK4/6 inhibitor therapy, offering a potential strategy for targeted intervention to limit TNBC metastasis.

## Synopsis

Triple negative breast cancers (TNBC) exhibit high rates of metastasis and have few treatment options. As TNBC are heterogeneous, subgroups may be vulnerable to specific treatments. We identify a miR-342-E2F network that regulates metastatic outgrowth of TNBC and is targetable by CDK4/6 inhibition.

- MiR-342 limits the metastatic outgrowth of disseminated TNBC cells.
- MiR-342 directly suppresses transcriptional and post-transcriptional networks that cooperatively dampen E2F signaling.
- TNBCs with reduced miR-342 and elevated E2F signaling are associated with poor outcomes.
- Pharmacological inhibition of E2F signaling using the CDK4/6 inhibitor palbociclib reduces metastatic outgrowth in a TNBC mouse model.

## Abstract (web summary)

Triple negative breast cancers (TNBC) exhibit high rates of metastasis and have few treatment options. As TNBC are heterogeneous, subgroups may be vulnerable to specific treatments. We identify a miR-342-E2F network that regulates metastatic outgrowth of TNBC and is targetable by CDK4/6 inhibition.

## Graphical abstract

**Figure:**
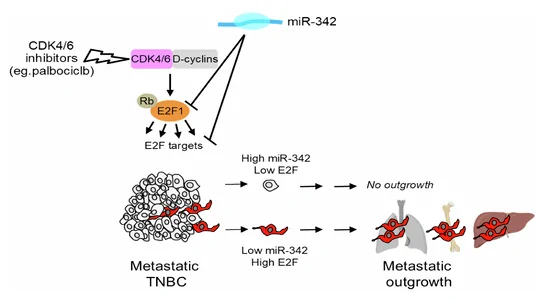

The paper explained **Problem**

The major challenges for the treatment of triple-negative breast cancer (TNBC) are its heterogeneous nature and its high metastatic capacity, leading to poor patient outcomes. Identifying factors that promote TNBC metastasis has the potential to reveal subgroups of TNBC patients where specific treatments may be particularly effective.

**Results**

In this study, we identify a multimodal miR-342-E2F network that specifically regulates metastatic outgrowth of TNBCs. TNBC patients with low levels of miR-342 and elevated E2F signalling are associated with metastatic progression and poor outcomes. Re-expression of miR-342 or treatment with the CDK4/6 inhibitor palbociclib reduced E2F signalling and suppressed metastatic outgrowth in pre-clinical mouse models.

**Impact**

Our findings reveal that TNBCs with low miR-342 expression and high E2F signalling display increased metastatic potential. This TNBC subgroup may be particularly responsive to CDK4/6 inhibitor therapy, offering a targeted strategy to limit TNBC metastasis.

## Introduction

Metastasis is the primary cause of death in breast cancer and occurs frequently in triple-negative breast cancer (TNBC), which lacks expression of oestrogen receptor (ER), progesterone receptor (PR) and human epidermal growth factor receptor 2 (HER2). Although metastatic TNBC often responds initially to chemotherapy, overall survival remains poor due to its intrinsic heterogeneity and the frequent emergence of drug resistance (Bianchini et al, 2022). Recent clinical trials have demonstrated that targeted therapies, such as immune checkpoint inhibitors and antibody-drug conjugates, can offer clinical benefit and modest survival improvements in patients with metastatic TNBC (Jiang et al, 2024; Yin et al, 2025). However, the pronounced heterogeneity of TNBC has complicated the development of targeted therapies and indicates that combinations of agents influencing multiple pathways may be required for effective treatment of metastatic disease.

MicroRNAs (miRNAs) are key regulators of gene expression and represent important diagnostic and promising therapeutic targets, due to their ability to regulate whole oncogenic networks of genes. Identifying miRNAs with clinical associations has the potential to reveal therapeutic vulnerabilities, including the need to co-target multiple pathways for clinical effectiveness. This is particularly critical in TNBC, which exhibits alterations in key proteins such as TP53, PIK3CA and PTEN, but lacks recurrent high-frequency driver mutations (Bianchini et al, 2016). Several miRNAs have been shown to influence breast cancer metastasis through inhibition of specific target genes, including miR-10b (RHOC), miR-335 (SOX4, TNC), miR-9 (CDH1, LIFR), miR-200 (ZEB1/2, SEC23A, MSN), miR-126 (CXCL12) and miR-21 (PDCD4, SERPINB5) among others (reviewed in (Pencheva and Tavazoie, 2013)). However, less is known about the spectrum of targets regulated by individual miRNAs and how these cooperate to influence breast cancer metastasis.

By profiling a clinically relevant mouse model of TNBC metastasis (4T1 and its variants) (Aslakson and Miller, 1992; Eckhardt et al, 2005), we identified several miRNAs that were associated with metastatic potential and also with poor outcome in TNBC patient cohorts. We focused on miR-342, which has some ascribed roles in breast cancer but whose function has not been investigated in metastatic progression. MiR-342 displays a positive association with ER expression and has been found to increase tamoxifen sensitivity in breast cancer lines and tumours (Cittelly et al, 2010; He et al, 2013). Clinical datasets reveal downregulation of miR-342 in inflammatory breast cancer (Van der Auwera et al, 2010), in tumours with early recurrence (Perez**-**Rivas et al, 2014) and in several small cohorts of TNBC (Enerly et al, 2011), suggestive of a tumour-suppressive role. More recently, miR-342-3p (the major mature form) was shown to repress TNBC cell growth in vitro through repression of monocarboxylate transporter 1 (MCT1) expression and consequent metabolic reprogramming (Romero-Cordoba et al, 2018). Despite these findings, the functional role and downstream targets of miR-342 in breast cancer metastasis remain largely uncharacterised.

The development of cyclin-dependent kinase (CDK) 4/6 inhibitors has significantly improved outcomes for women with metastatic hormone receptor-positive breast cancer, and combined with endocrine therapy, is now considered a frontline treatment for this disease (Morrison et al, 2024). CDK4/6 inhibitors function by preventing phosphorylation of the retinoblastoma (RB) protein, thereby suppressing downstream E2F-driven signalling that drives breast cancer growth. The therapeutic effectiveness of CDK4/6 inhibitors is thought to be largely contingent upon the presence of functional RB, which is typically retained in ER+ breast cancers but is lost with higher frequency in TNBCs (Cancer Genome Atlas N 2012; Patel et al, 2020; Trere et al, 2009). Consequently, CDK4/6 inhibitors are currently not in routine clinical use for TNBC; however, there are indications that CDK4/6 inhibitors may be effective in subgroups of TNBC (Asghar et al, 2017; Christenson et al, 2021). Despite their widespread use, the impact of CDK4/6 inhibitors on metastatic progression remains an area not well understood.

In this study, we demonstrate the existence of a low miR-342/high E2F signalling TNBC subgroup that has increased metastatic competency and is amenable to CDK4/6 inhibitor therapy. Mechanistically, miR-342 directly represses multiple components of the E2F1 signalling network, with the cumulative effect of dampening E2F signalling and reducing metastatic outgrowth. We show that treatment of established TNBC metastases with miR-342 or the CDK4/6 inhibitor palbociclib reduces E2F signalling and specifically limits metastatic outgrowth. Our findings offer a potential strategy for preventing metastatic outgrowth and improving outcomes in low miR-342/high E2F signalling subgroups of TNBC.

## Results

### Low miR-342 expression is associated with increased metastatic potential and poor patient outcome in TNBC

**Figure 1:**
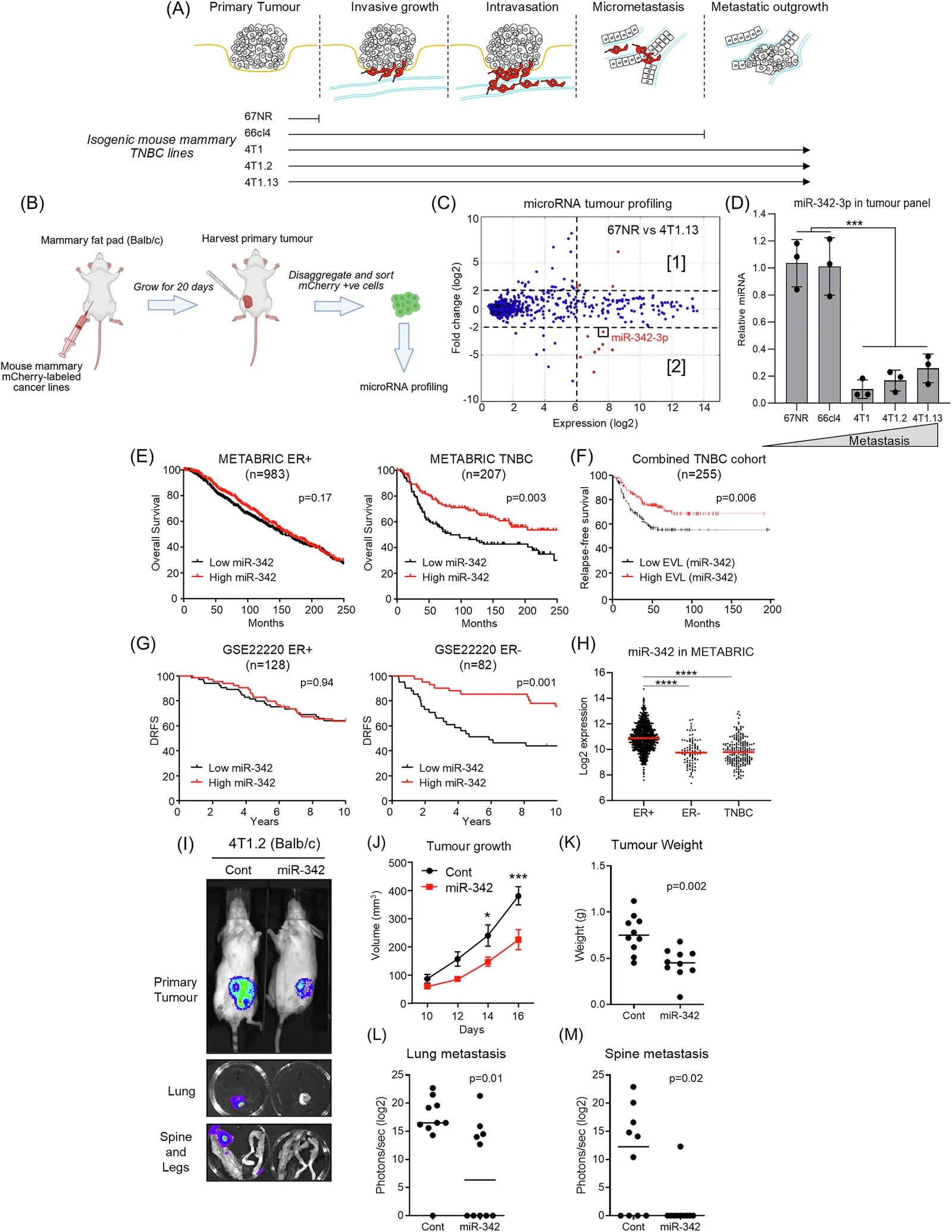
Low miR-342 expression is associated with TNBC metastatic progression, and its expression suppresses metastasis in a syngeneic mouse model. (**A**) Schematic of metastatic cascade showing the metastatic propensity of isogenic mouse mammary TNBC cell lines of the 4T1 panel. (**B**) Schematic showing orthotopic implantation of 4T1 panel cell lines labelled with mCherry followed by primary tumour harvest, isolation of mCherry tumour cells by FACS, and microRNA profiling. (**C**) Scatter MA plot of differential miRNA expression in weakly (67NR) vs highly metastatic (4T1.13) isogenic tumours. MiRNAs highlighted in red in quadrants 1 and 2 are in the top 10% of expressed genes with >fourfold change. (**D**) Quantitative PCR of miR-342-3p in tumours derived from the 4T1 model (*n* = 3). Significance was measured by one-way ANOVA with Tukey post hoc test. *P* values for 67NR vs 4T1 (*P* = 0.0001), 67NR vs 4T1.2 (*P* = 0.0001), 67NR vs 4T1.13 (*P* = 0.0003), 66cl4 vs 4T1 (*P* = 0.0001), 66cl4 vs 4T1.2 (*P* = 0.0002), and 66cl4 vs 4T1.13 (*P* = 0.0005) are indicated as ***P < 0.001. (**E**) Kaplan–Meier (KM) survival analysis on median split of miR-342 in ER+ (left) and TNBC (right) breast tumours from the METABRIC database. Significance was measured by the Log-rank (Mantel–Cox) test. (**F**) KM survival analysis of miR-342 host gene EVL in TNBC combined from 26 cohorts from the KM plotter database (Gyorffy et al, 2010) with significance measured using the Log-rank (Mantel–Cox) test. (**G**) KM survival analysis on median split of miR-342 in ER+ (left) and TNBC (right) TCGA breast cancer tumours. Significance was measured by the Log-rank (Mantel–Cox) test. (**H**) MiR-342 expression in METABRIC breast cancer ER+ (*n* = 983), ER− (*n* = 93), and TNBC (*n* = 207) subsets. Significance was calculated using one-way ANOVA with Tukey post hoc test ****P < 0.0001. (**I**) Bioluminescence imaging (BLI) of primary tumours, lungs and spine and femurs ex vivo of control (Cont) or miR-342 expressing 4T1.2 tumours 16 days after orthotopic injection. (**J**) Tumour growth measured by calliper volume measurements at the indicated times. Mean ± SEM are shown (*n* = 10/group). Two-way ANOVA with Bonferroni post hoc test. *P* values are Cont vs miR-342 d14 (*P* = 0.0407) and Cont vs miR-342 d16 (*P* = 0.0001) and are indicated as *P < 0.05, ***P < 0.001. (**K**) Tumour weight measured at harvest on day 16 (*n* = 10). (**L**, **M**) Lung metastasis and spine metastasis measured by ex vivo BLI at harvest and normalised to tumour weight (*n* = 10). Significance for (**K**–**M**) was calculated by a two-tailed Mann–Whitney test. Source data are available online for this figure.

The 4T1 panel comprises well-characterised isogenic TNBC cell lines with distinct metastatic capacities, ranging from non-metastatic (67NR), to weakly metastatic (66cl4), to highly metastatic (4T1, 4T1.2, 4T1.13) phenotypes (Aslakson and Miller, 1992; Eckhardt et al, 2005) (Fig. 1A). To identify miRNAs that differ in expression in TNBC tumour cells in an in vivo context, we profiled miRNA expression in 4T1 panel tumours by orthotopic implantation of cells into the mammary fat pad of Balb/c mice and subsequent flow cytometric isolation of mCherry-labelled tumour cells (Fig. 1B). MiRNA profiling identified twelve candidate miRNAs whose expression ranked in the top 10% of all detected miRNAs and differed by more than fourfold between the least (67NR) and most metastatic (4T1.13) tumours (Fig. 1C; Dataset EV1). Among these, we focused on miR-342, which has been implicated in breast cancer progression (Cittelly et al, 2010; Enerly et al, 2011; He et al, 2013; Perez**-**Rivas et al, 2014; Romero-Cordoba et al, 2018; Van der Auwera et al, 2010), although its role in TNBC remains poorly defined. Quantitative PCR confirmed that miR-342 expression in metastatic tumours was reduced to less than 25% of that in non-metastatic counterparts and correlated with increased metastatic outgrowth (Fig. 1D).

**Figure EV1:**
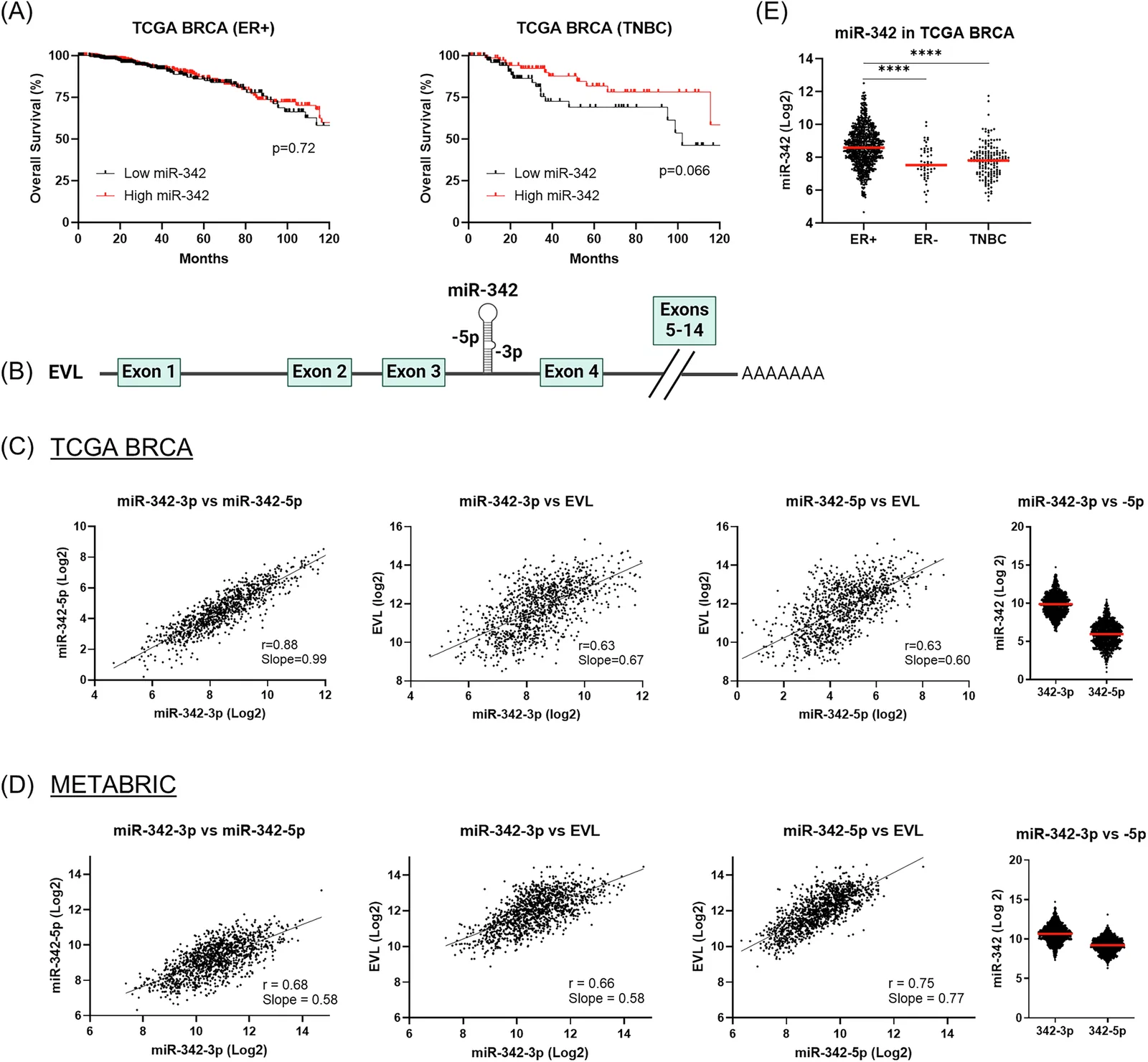
miR-342 and EVL expression in breast cancer datasets. (**A**) Kaplan–Meier (KM) overall survival analysis on median split of miR-342 in ER+ (left) and TNBC (right) breast tumours (BRCA) from the TCGA database. Significance was measured by the Log-rank (Mantel–Cox) test. (**B**) Schematic of *EVL* gene locus showing the location of intronic miR-342. (**C**, **D**) Scatter plots showing positive correlations of miR-342-3p, miR-342-5p and EVL in TCGA and METABRIC datasets. Pearson correlation coefficients and associated *P* values are indicated. Relative expression of miR-342-3p and miR-342-5p in TCGA and METABRIC cohorts is shown. (**E**) MiR-342 expression in TCGA breast cancer subsets. Significance was measured by one-way ANOVA with Tukey post hoc test ****P < 0.0001.

To determine whether miR-342 is associated with metastatic potential in clinical datasets, we analysed large patient cohorts with long-term outcome data. Analysis of these datasets demonstrated miR-342 as a consistent and independent predictor of patient outcomes in TNBC. In both the METABRIC and TCGA cohorts (Cancer Genome Atlas N, 2012; Curtis et al, 2012), low miR-342 expression was significantly associated with reduced overall survival among TNBC patients, but not oestrogen receptor (ER) positive patients (Figs. 1E and  EV1A). MiR-342 gives rise to two mature miRNAs, miR-342-3p and miR-342-5p, which are co-expressed from the same precursor located within intron 3 of its host gene, *EVL* (Fig. EV1B). Although miR-342-5p is expressed at markedly lower levels than miR-342-3p, both miRNAs exhibit a strong positive correlation with *EVL* expression (Fig. EV1C,D). A meta-analysis of *EVL* across 26 TNBC cohorts (Gyorffy et al, 2010) also demonstrated its prognostic value, with higher *EVL* levels associated with improved relapse-free survival (Fig. 1F). While miR-342 expression is positively associated with ER status across datasets and is notably downregulated in ER-negative and TNBC tumours (Figs. 1H and  EV1E), it was not predictive of outcome in ER+ tumours in either the METABRIC or TCGA cohorts (Figs. 1E and  EV1A). These findings are consistent with a prior study, which reported that low miR-342 levels were significantly associated with poorer distant relapse-free survival in ER-negative (hazard ratio [HR] = 0.48) and TNBC (*n* = 37, HR = 0.33) patients, but not in ER+ cases, even after adjusting for age, tumour size/grade, nodal status, and chemotherapy treatment (Buffa et al, 2011) (Fig. 1G). Together, our clinical and experimental data demonstrate that miR-342 is downregulated in highly metastatic breast cancer cell lines and that reduced miR-342 expression is a marker of poor prognosis in TNBC.

### MiR-342 suppresses metastasis in mouse and human TNBC models

**Figure EV2:**
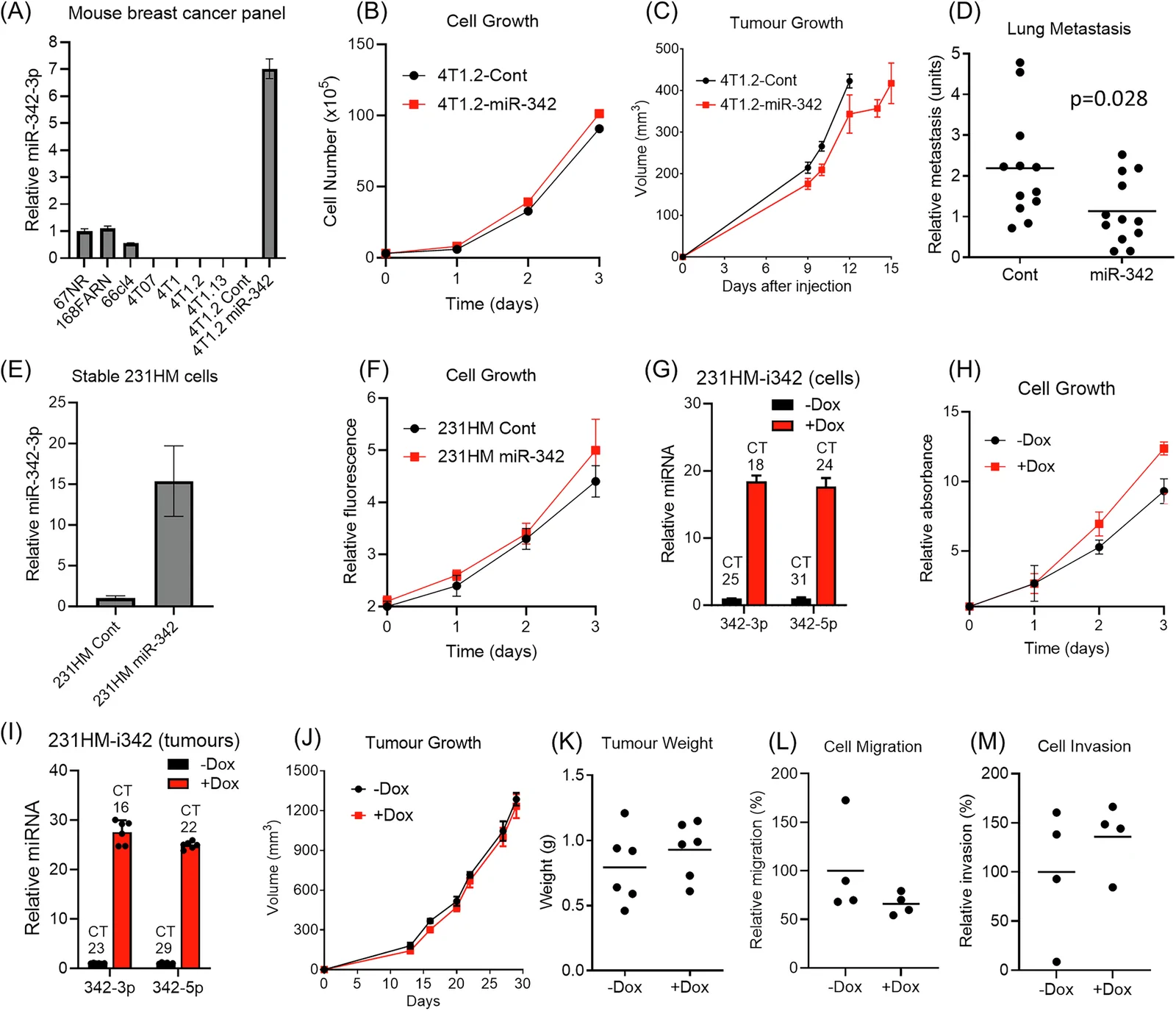
miR-342 expression and function in mouse and human breast cancer cell lines. (**A**) Relative expression of miR-342-3p in cells of the 4T1 panel measured by qPCR. The relative level of miR-342-3p in control (empty vector) or miR-342 overexpressing 4T1.2 cells is shown for comparison. Data are plotted as mean ± SD of three technical replicates. (**B**) Proliferation assay of 4T1.2 control and miR-342 overexpressing cell lines. (**C**) Growth of 4T1.2 control and miR-342 overexpressing tumours following mammary fat pad implantation. The experimental endpoint was determined by equivalent tumour volume. Data are plotted as mean ± SEM. *n* = 12 mice/group. (**D**) Lung metastasis measured at experimental endpoint (d12 for Cont; d15 for miR-342) by genomic DNA qPCR of mouse tumour cells (mCherry) relative to total mouse cells (mouse Vimentin), *n* = 12 mice/group. Significance was calculated by a two-tailed Mann–Whitney test. (**E**) Relative expression of miR-342-3p in control (empty vector) or miR-342 overexpressing 231HM cell lines. Data are plotted as mean ± SD of three technical replicates. (**F**) Proliferation assay of 231HM control and miR-342 overexpressing cell lines, as measured by the CyQUANT fluorescence assay. Data are plotted as mean ± SEM (*n* = 4). (**G**) Relative expression of miR-342-3p and -5p in 231HM-i342 cells following 4 days of doxycycline (Dox) induction, as measured by qPCR. Data are plotted as mean ± SD of three technical replicates. Average cycle thresholds (CT) are shown above each bar. (**H**) Growth assay of 231HM-i342 following 4 days of Dox induction (1 day pre-treatment). Mean ± SD (*n* = 4). (**I**) Relative expression of miR-342-3p and -5p in 231HM-i342 tumours at experimental endpoint measured by qPCR (day 30). Mean ± SD (*n* = 6). Average cycle thresholds (CT) are shown above each bar. (**J**) Volumes of 231HM-i342 tumours with or without Dox exposure. Mean ± SEM (*n* = 6). (**K**) Weight of 231HM-i342 tumours at experimental endpoint (day 30). The mean of six tumours per group is shown. Significance was calculated using a two-tailed Mann–Whitney test. (**L**, **M**) Relative cell migration (5 hr) and invasion (24 hr) of 231HM-i342 cells after 4 days of Dox induction. Mean of 4 biological replicates is shown. Significance was calculated by two-tailed Student’s *t* test.

To investigate the functional role of miR-342 in TNBC metastasis, we stably overexpressed miR-342 in highly metastatic 4T1.2 cells (Fig. EV2A). While increased miR-342 expression had no effect on in vitro proliferation (Fig. EV2B), it significantly suppressed tumour growth following orthotopic implantation into the mammary fat pad of Balb/c mice (Fig. 1I–K). Bioluminescence imaging revealed miR-342 expression markedly reduced metastatic spread to the lungs and spine, even after normalising for differences in primary tumour weight (Fig. 1L,M). To confirm these findings, we conducted a second experiment in which tumours were harvested at matched sizes, thereby minimising the potential confounding effect of primary tumour burden on metastatic outcome. Notably, even when allowed to grow for a longer duration, miR-342-expressing tumours displayed significantly reduced metastatic capacity (Fig. EV2C,D). In this case, metastasis was measured by quantitative PCR of genomic DNA, where the amount of tumour cells is quantitated in whole lungs by the ratio of tumour-specific mCherry gDNA to total gDNA levels (Chi et al, 2024; Eckhardt et al, 2005). This orthologous method has the advantage of being independent of the time and metabolism-dependent bioluminescent reaction, with the potential disadvantage of reduced sensitivity, and has been used in conjunction with bioluminescence imaging in several studies (Chang et al, 2023; Samuel et al, 2021; Shekhar et al, 2019). Employing these complementary measurements, a significant reduction in lung metastasis was observed in both experiments, implicating miR-342 as a potent suppressor of TNBC metastasis.

**Figure 2:**
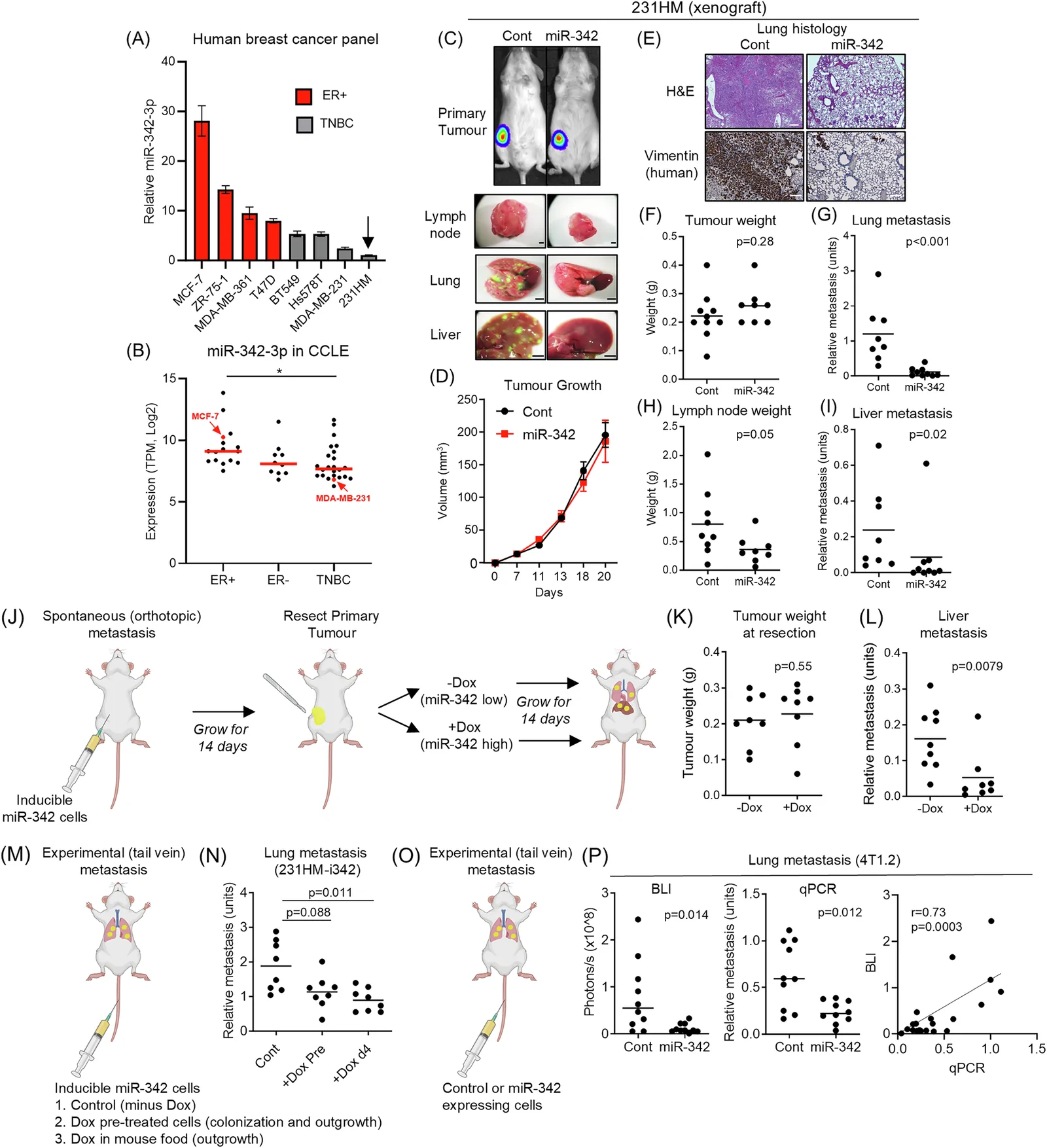
MiR-342 specifically suppresses metastatic outgrowth in mouse and human TNBC models. (**A**) Expression of miR-342-3p in a panel of human cell lines measured by qPCR. Data are plotted as mean ± SD of three technical replicates. The MDA-MB-231HM (231HM, highly metastatic) cell line used in xenograft models is indicated with an arrow. (**B**) Relative expression of miR-342-3p in the cancer cell line encyclopedia (CCLE) for breast cancer cell lines grouped according to their pathological subtype—ER+ (*n* = 16), ER− (*n* = 10), and TNBC (*n* = 24). Significance was measured by one-way ANOVA with Tukey post hoc test. *P* value for ER+ vs TNBC (*P* = 0.0205) is indicated as *P < 0.05. (**C**) Bioluminescence imaging (BLI) of primary tumours and images of axillary lymph nodes, lung and liver of control or miR-342 expressing 231HM cells at day 20 after orthotopic injection. Metastases on the lung and liver are shown by green pseudo-colour overlay. Scale bars represent 2 mm. (**D**) Primary tumour growth measured by calliper volume measurements at indicated times, mean ± SEM (*n* = 10/group). (**E**) Histology of lung sections to visualise metastases with haematoxylin & eosin or a human-specific vimentin antibody. Scale bars represent 100 µm. (**F**) Tumour weight measured at time of tumour resection (day 20, *n* = 10 for control, *n* = 8 for miR-342). Significance was calculated by a two-tailed Mann–Whitney test. (**G**) Lung metastasis measured at experimental endpoint (21 days after tumour resection) by genomic DNA qPCR of human tumour cells (mCherry) relative to mouse cells (mouse Vimentin). *n* = 8 for control, *n* = 9 for miR-342. Significance was calculated by a two-tailed Mann–Whitney test. (**H**) Lymph node weight measured at experimental endpoint (21 days after tumour resection). *n* = 10 for control, *n* = 8 for miR-342. Significance was calculated by a two-tailed Mann–Whitney test. (**I**) Liver metastasis measured at experimental endpoint (21 days after tumour resection) by genomic DNA qPCR of human tumour cells (mCherry) relative to mouse cells (mouse Vimentin). *n* = 8 for control, *n* = 9 for miR-342. Significance was calculated by a two-tailed Mann–Whitney test. (**J**) Schematic of orthotopic metastasis experiment with temporal doxycycline-mediated induction of miR-342 in 231HM-i342 cells. (**K**) Tumour weight measured at time of tumour resection (day 14, *n* = 8). Significance was calculated by a two-tailed Mann–Whitney test. (**L**) Liver metastasis measured at experimental endpoint (14 days after tumour resection) by genomic DNA qPCR of human tumour cells (GFP) relative to mouse cells (mouse Vimentin). Significance was calculated by a two-tailed Mann–Whitney test (*n* = 9 for control, *n* = 8 for miR-342). (**M**) Schematic of intravenous metastasis experiment with temporal doxycycline-mediated induction of miR-342 in 231HM-i342 cells. (**N**) Lung metastasis measured at experimental endpoint (day 22) by genomic DNA qPCR of human tumour cells (GFP) relative to mouse cells (mouse Vimentin). Significance was calculated by one-way ANOVA with Kruskal–Wallis tests (*n* = 8). (**O**) Schematic of intravenous metastasis experiment with control or miR-342 expressing 4T1.2 cells. (**P**) Lung metastasis measured by ex vivo bioluminescence imaging (BLI, left panel) or by genomic DNA qPCR of murine tumour cells (mCherry) relative to all mouse cells (mouse Vimentin) in lung tissue (qPCR, middle panel) at harvest at experimental endpoint (day 13). Significance was calculated by a two-tailed Mann–Whitney test (*n* = 10). Correlation of BLI and qPCR data is shown in the right-hand panel, with Pearson correlation coefficient and *P* value indicated. Source data are available online for this figure.

To evaluate whether miR-342 suppresses metastasis in human TNBC models, we employed a highly metastatic variant of MDA-MB-231 cells (231HM) that spontaneously metastasises to the lymph nodes, lungs and liver following orthotopic implantation into the mammary fat pad (Chang et al, 2008; Johnstone et al, 2018). Compared to parental MDA-MB-231 cells, 231HM cells exhibited lower miR-342 expression and had the lowest levels among a panel of human breast cancer cell lines, mirroring clinical observations where ER+ lines generally express higher levels of miR-342 than ER-negative and TNBC lines (Fig. 2A,B). We stably overexpressed miR-342 in 231HM cells to levels similar to those in ER+ breast cancer lines and found that this had minimal impact on in vitro proliferation (Figs. 2B and EV2E,F) and also did not alter primary tumour growth in NOD-SCIDγ (NSG) mice (Fig. 2C,D,F). However, miR-342 expression significantly reduced metastatic dissemination to the axillary lymph nodes as measured by weight (Fig. 2H), and to lung and liver as measured by reduced detection of human-specific vimentin by immunohistochemistry (Fig. 2E) or by quantitation of tumour-specific mCherry gDNA levels (Fig. 2G,I) in these immunodeficient mice. Collectively, these findings demonstrate that miR-342 exerts robust anti-metastatic activity in both immune-competent and immune-deficient models of TNBC.

### MiR-342 is required for the growth of breast cancer cells with high endogenous miR-342 expression

**Figure EV3:**
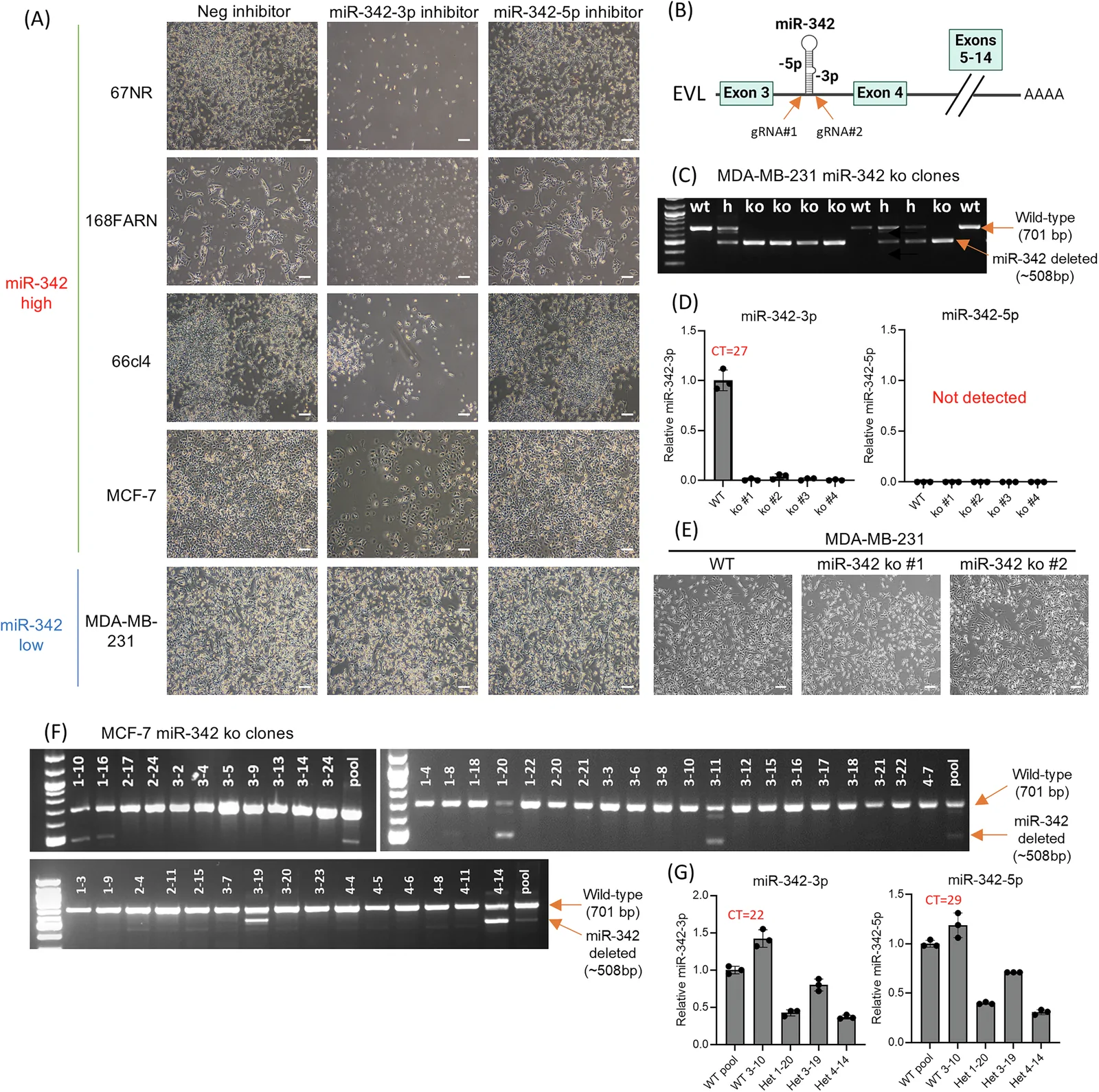
miR-342 inhibition in mouse and human breast cancer cell lines. (**A**) Phase contrast images of miR-342 high or miR-342 low expressing mouse and human breast cancer cell lines following 72 h transfection with control, miR-342-3p, or miR-342-5p Anti-miRs. Scale bars represent 50 µm. (**B**) Schematic of miR-342 knockout (ko) approach using dual guide RNAs to cut out pre-miR-342 from the *EVL* host gene. (**C**) Gel showing PCR screen of genomic DNA isolated from MDA-MB-231 miR-342 ko clones (wt wild-type, h heterozygous). The size of the wild-type and full deletion of miR-342 based on the gRNA cut sites is shown. (**D**) Quantitation of miR-342-3p and -5p levels in WT and 4 different miR-342 ko clones. Cycle threshold (CT) of miR-342-3p in WT is shown. (**E**) Phase contrast images of WT and representative miR-342 ko clones. Scale bars represent 50 µm. (**F**) Gels showing PCR screen of genomic DNA isolated from MDA-MB-231 miR-342 ko clones (clone numbers shown above; pool is cells collected after being transfected with gRNA#1 and #2). The size of the wild-type and full deletion of miR-342 based on the gRNA cut sites is shown. (**G**) Quantitation of miR-342-3p and -5p levels in WT pool (parental cells), a WT single clone, and 3 different miR-342 heterozygous clones. The Cycle Threshold (CT) of miR-342-3p and 5p expression in WT pool is shown in red.

To determine whether endogenous miR-342 suppresses metastatic potential in mouse and human breast cancer cell lines, we first examined the effects of transient miRNA inhibition in four cell lines that intrinsically express high levels of miR-342 and display low metastatic potential (Figs. 1A,D, 2A,B, and EV2A). Unexpectedly, inhibition of miR-342-3p markedly reduced the growth of all four cell lines, whereas cells transfected with a miR-342-5p inhibitor behaved similarly to negative control-transfected cells (Fig. EV3A). In contrast, inhibition of miR-342-3p had no effect on the growth of MDA-MB-231 cells, which express intrinsically low levels of miR-342 (Fig. 2A,B and EV3A). As miR-342-3p is the predominant mature product generated from the miR-342 precursor (Fig. EV1C,D), these findings suggest that cells with high endogenous miR-342-3p expression may depend on this miRNA for proliferation.

To validate this observation using an independent approach, we employed a paired-guide RNA CRISPR strategy to delete the miR-342 precursor sequence in MDA-MB-231 cells (Fig. EV3B). This approach generated many miR-342 knockout clones, which were confirmed by genomic PCR and quantitative PCR analysis of miR-342-3p and miR-342-5p expression (Fig. EV3C,D). Consistent with the transient inhibition experiments, miR-342 knockout clones exhibited normal growth and were indistinguishable from wild-type MDA-MB-231 cells (Fig. EV3E). We next applied the same strategy to MCF-7 cells, which express high endogenous levels of miR-342 (Fig. 2A,B). However, despite extensive screening, no complete miR-342 knockout clones were recovered, and only a small number of heterozygous clones were found (Fig. EV3F,G). Together, these findings indicate that miR-342 is important for the growth of breast cancer cells with low metastatic potential that express high endogenous levels of this miRNA. We hypothesise that highly metastatic TNBC cells, which express low levels of miR-342, have acquired alternative mechanisms to bypass this requirement, potentially through the activation of miR-342 target genes and downstream pathways.

### MiR-342 suppresses metastatic outgrowth of TNBC

The ability of miR-342 to suppress metastasis across distinct TNBC models indicates that it likely functions through tumour cell-intrinsic mechanisms. However, whether miR-342 influences specific steps within the metastatic cascade, such as tumour cell dissemination, colonisation, or secondary outgrowth, remains unclear. To investigate this, we developed a doxycycline (Dox)-inducible system to temporally control miR-342 expression in 231HM cells (231HM-i342). Consistent with results from the stable expression model, Dox-induced miR-342 expression had minimal impact on in vitro cell proliferation or primary tumour growth in vivo when mice were continuously fed Dox-containing chow (Fig. EV2G–K). To explore whether miR-342 affects early metastatic events, we first assessed its impact on cell migration and invasion. Induction of miR-342 did not significantly alter these behaviours in vitro, suggesting that miR-342 may instead act on later stages of metastasis (Fig. EV2L,M). To test this, we orthotopically implanted 231HM-i342 cells into the mammary fat pad, allowed tumours to grow for 14 days, then surgically resected them. Mice were subsequently divided into two groups: one receiving standard chow and the other Dox-containing chow (Fig. 2J). Fourteen days after resection, induction of miR-342 significantly reduced liver metastases, indicating its ability to suppress metastatic colonisation and/or outgrowth (Fig. 2K,L).

To distinguish between the impact on the initial colonisation or on the outgrowth of metastases, we performed tail vein injection assays that bypass the initial step of dissemination from the primary tumour. 231HM-i342 cells were either treated with Dox for two days prior to injection or the mice were administered Dox-containing food commencing four days after inoculation, before evaluation of lung metastasis on day 22 (Fig. 2M). In both scenarios, miR-342 induction reduced metastatic outgrowth, with a larger and significant effect observed when miR-342 was activated after tumour cells had colonised the lung (Fig. 2N). Similarly, stable expression of miR-342 in 4T1.2 cells markedly suppressed lung metastasis 13 days after tail vein injection, as assessed by bioluminescence imaging and confirmed by quantitative PCR of tumour-specific DNA in lung tissue, with the two methods yielding highly concordant results (Fig. 2O,P). Together, these findings demonstrate that miR-342 inhibits the later stages of metastasis and most prominently metastatic outgrowth in TNBC models.

### Identification of the global complement of miR-342 target genes

**Figure 3:**
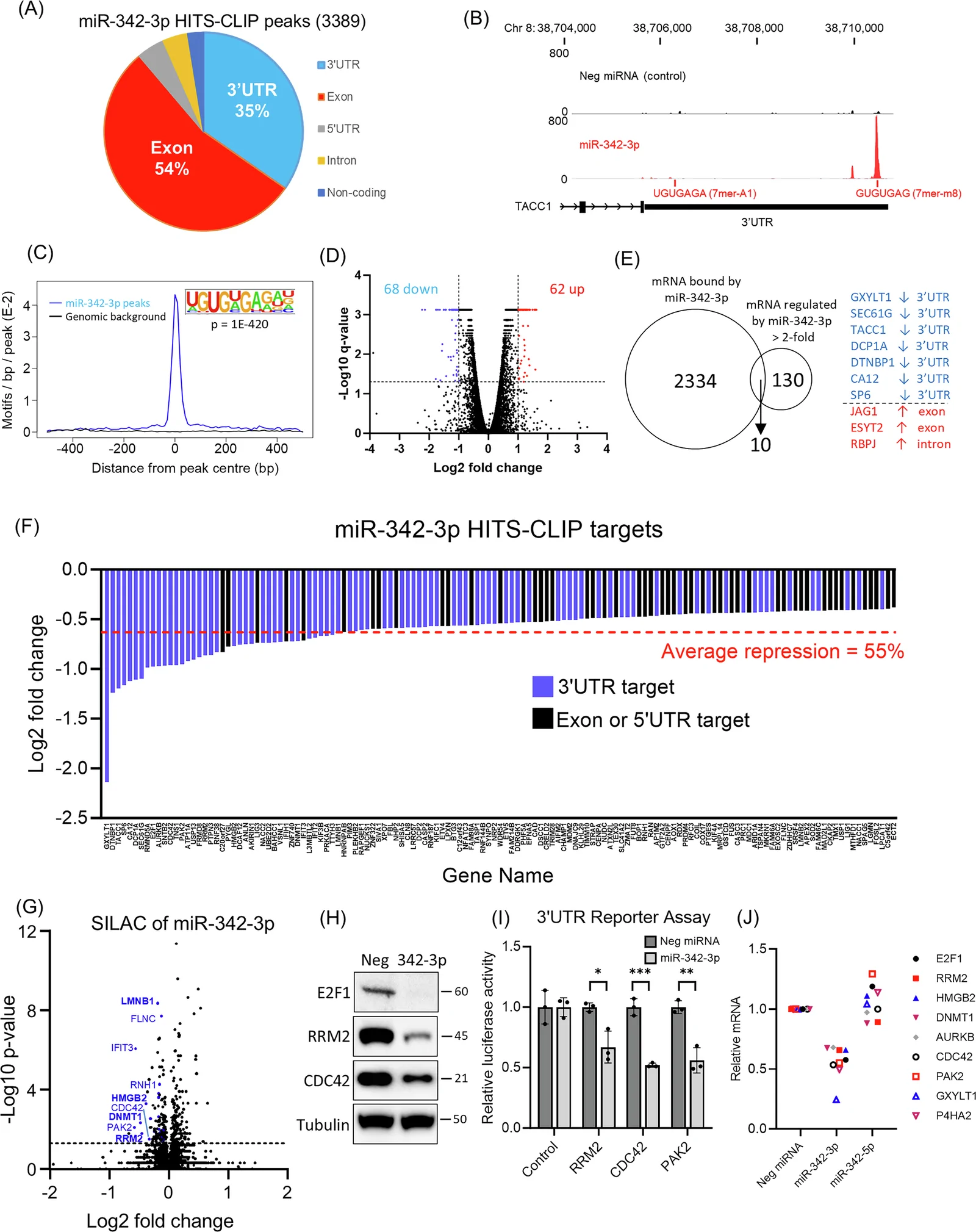
Identifying the global complement of miR-342 target genes in TNBC. (**A**) Pie chart showing the distribution of miR-342-3p Ago-HITS-CLIP peaks in 231HM cells by genomic location type. (**B**) Example of a miR-342-3p peak in the TACC1 gene. MiR-342-3p seed recognition sequences in the TACC1 3’UTR are shown, with Ago-loaded miR-342-3p binding only to the distal site. Genome tracks represent the average read density of all replicates for each condition. (**C**) Distribution of miR-342-3p recognition sequences within Ago-HITS-CLIP peaks. The most enriched motif, corresponding to miR-342-3p, and the associated *P* value is shown. Background represents the occurrence of the motif on the opposite strand of the peak. (**D**) Volcano plot showing expression of genes altered by miR-342-3p transfection in 231HM cells (*n* = 2 biological replicates). Blue and red dots indicate significantly downregulated and upregulated genes changing >twofold (*q* value < 0.05). (**E**) Venn diagram showing overlap of mRNAs significantly regulated by miR-342-3p by greater than twofold with those having a miR-342-3p HITS-CLIP peak. For the overlapping genes, the direction of regulation and location of the HITS-CLIP peaks are shown. (**F**) Ranked bar chart showing change in expression of high-confidence miR-342-3p target genes upon miR-342-3p transfection. The location of the miR-342-3p HITS-CLIP is indicated by colour, with the dotted line marking the average degree of repression across all mRNAs. (**G**) Volcano plot showing levels of proteins altered by miR-342-3p transfection in 231HM cells (*n* = 2 biological replicates). Blue dots indicate significantly downregulated proteins (*P* value < 0.05) that are also verified miR-342-3p target genes. Proteins in bold represent E2F pathway genes. Significance assessed with the Mann–Whitney *U* test. (**H**) Western blot of selected miR-342-3p target genes following miR-342-3p or control miRNA transfection in 231HM cells. (**I**) Luciferase reporter assay of control (no 3’UTR) or selected miR-342-3p target gene 3’UTRs transfected with miR-342-3p or control miRNA in 231HM cells. Data are plotted as mean ± SD (*n* = 3). Significance was calculated by two-tailed Student’s *t* test. *P* values for RRM2 with neg miRNA vs miR-342-3p (*P* = 0.0140), CDC42 with neg miRNA vs miR-342-3p (*P* = 0.0003), and PAK2 with neg miRNA vs miR-342-3p (*P* = 0.0029) are indicated as *P < 0.05, **P < 0.01, ***P < 0.001. (**J**) Quantitative PCR of selected miR-342-3p target genes following miR-342-3p, miR-342-5p or control miRNA transfection in 231HM cells. Source data are available online for this figure.

**Figure EV4:**
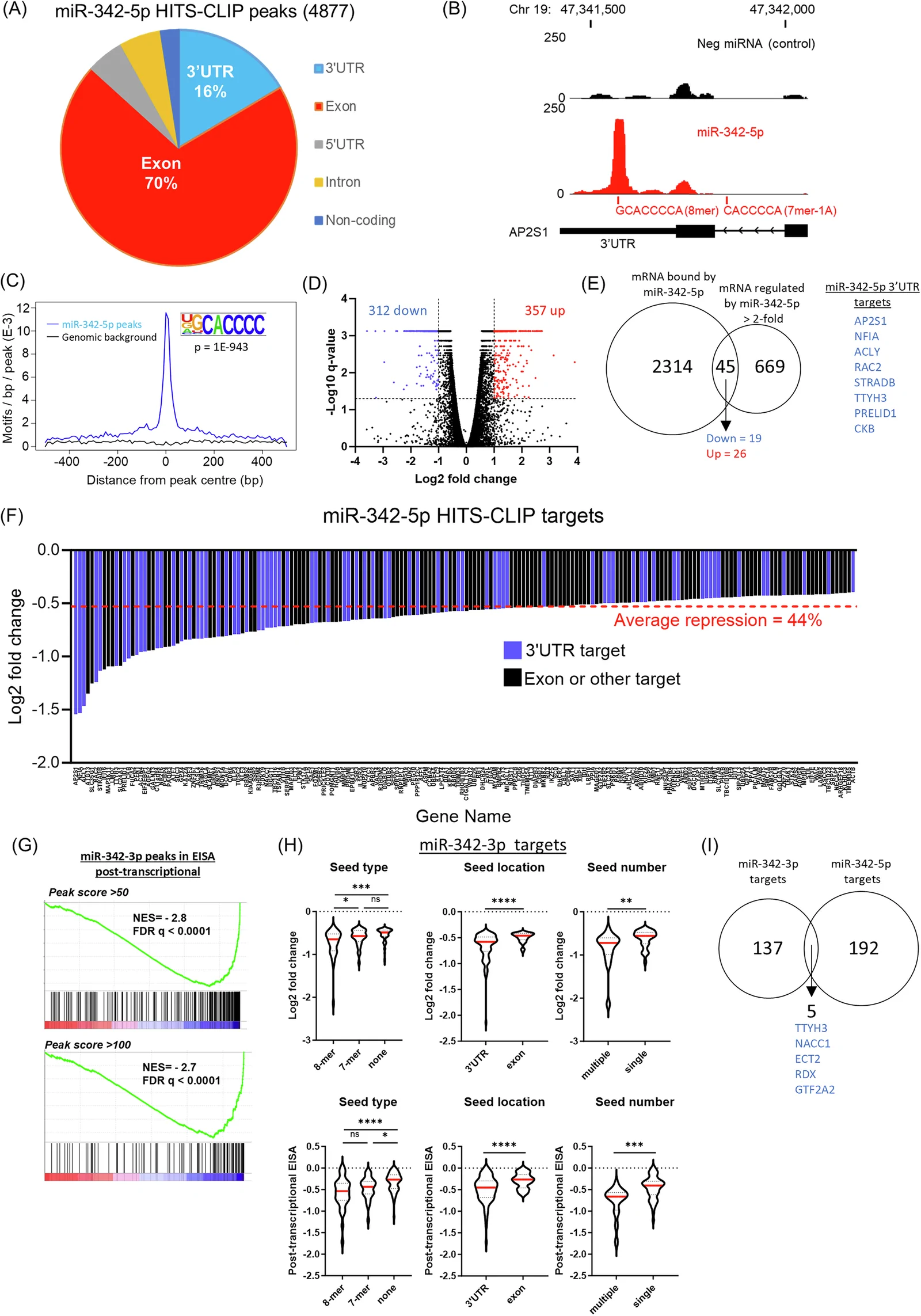
Identification of miR-342-5p target genes in TNBC. (**A**) Pie chart showing the distribution of miR-342-5p Ago-HITS-CLIP peaks in 231HM cells by genomic location type. (**B**) Example of an Ago-HITS-CLIP peak in the AP2S1 gene. MiR-342-5p seed recognition sequences in the AP2S1 3’UTR are shown, with Ago-loaded miR-342-5p binding only to the 3’UTR binding site. Genome tracks represent the average read density of all replicates for each condition. (**C**) Distribution of miR-342-5p recognition sequences within Ago-HITS-CLIP peaks. The most enriched motif, corresponding to miR-342-5p, and the associated *P* value are shown. Background represents the occurrence of the motif on the opposite strand of the peak. (**D**) Volcano plot showing expression of genes altered by miR-342-5p transfection in 231HM cells (*n* = 2 biological replicates). Blue and red dots indicate significantly downregulated and upregulated genes, respectively, changing >twofold (*q* value < 0.05). (**E**) Venn diagram showing overlap of mRNAs significantly regulated by miR-342-5p with those having a miR-342-5p HITS-CLIP peak. For the overlapping genes, those that are repressed and contain 3’UTR binding sites are indicated. (**F**) Ranked bar chart showing change in expression of high-confidence miR-342-5p target genes upon miR-342-5p transfection. The location of the miR-342-3p HITS-CLIP is indicated by colour, with the dotted line marking the average degree of repression across all mRNAs. (**G**) Gene set enrichment analysis (GSEA) demonstrating increasing repression of miR-342-5p HITS-CLIP targets with a peak score of >50 or >100. Normalised enrichment scores (NES) and FDR are indicated. (**H**) Violin plots showing the degree of total repression (upper) and post-transcriptional repression (lower) for mRNA with different miR-342-3p seed types, locations and numbers. Significance was measured by one-way ANOVA with Tukey post hoc test (3 groups) or unpaired *t* test (2 groups). *P* values for gene expression for seed type 8-mer vs 7-mer (*P* = 0.0383), seed type 8-mer vs none (*P* = 0.0001), seed number multiple vs single (*P* = 0.0033) and other indicated comparisons are shown as ns—not significant, *P < 0.05, **P < 0.01, ***P < 0.001, ****P < 0.0001. *P* values for post-transcriptional expression for seed type 7-mer vs none (*P* = 0.0456), seed number multiple vs single (*P* = 0.0003) and other indicated comparisons are shown as ns—not significant, *P < 0.05, ***P < 0.001, ****P < 0.0001. (**I**) Venn diagram showing overlap between miR-342-3p and -5p direct targets. The overlapping genes are indicated.

MiRNAs can theoretically regulate hundreds of genes and modulate multiple pathways to influence cellular behaviour. To uncover the molecular mechanisms by which miR-342 impacts TNBC metastasis, we employed a multi-omics strategy combining biochemical pulldown of direct miR-342 targets with integrative analysis of RNA sequencing (RNA-seq) and proteomic data. As a first step, we performed Argonaute high-throughput sequencing of RNA isolated by crosslinking immunoprecipitation (Ago-HITS-CLIP (Chi et al, 2009)) in 231HM cells transfected with control, miR-342-3p, or miR-342-5p mimics. Following immunoprecipitation of the Ago complex, miR-342-bound mRNAs were identified by high-throughput sequencing. Analysis of Ago binding peaks enriched in miR-342-transfected cells relative to controls revealed that most peaks were located within annotated genes, with a notably higher proportion of miR-342-5p peaks occurring within coding regions (Figs. 3A and  EV4A). Ago-HITS-CLIP also identified high-confidence binding events where multiple miR-342 seed sites were present (Figs. 3B and  EV4B), as well as non-canonical peaks lacking traditional seed sequences. In total, we identified 3393 miR-342-3p peaks across 2,334 genes and 4877 miR-342-5p peaks across 2314 genes (Datasets EV2 and EV3). De novo motif analysis confirmed that the most enriched sequences within these peaks corresponded to the seed regions of miR-342-3p and miR-342-5p, with these motifs predominantly located at the centre of the peaks, highlighting the specificity and robustness of this approach for identifying direct miR-342 targets (Figs. 3C and  EV4C).

To identify mRNAs directly repressed by miR-342-3p or miR-342-5p, we performed RNA-seq under the same conditions used for Ago-HITS-CLIP. Interestingly, miR-342-3p induced relatively modest changes in gene expression, with only 130 transcripts showing greater than twofold upregulation or downregulation. In contrast, miR-342-5p had a broader impact, modulating the expression of over 600 transcripts by more than twofold (Figs. 3D and  EV4D). Integration of the RNA-seq and Ago-HITS-CLIP datasets revealed limited overlap between mRNAs exhibiting >twofold changes in expression and those bound by Argonaute (Figs. 3E and EV4E; Datasets EV2 and EV3). Among the ten miR-342-3p target mRNAs identified, seven were downregulated and contained binding sites within their 3′ untranslated regions (3′UTRs). For miR-342-5p, 19 of 45 mRNAs with identified binding sites were downregulated, though only 8 of these had sites in their 3′UTR, suggesting that miR-342-5p may potentially act through non-canonical mechanisms, including binding within coding regions.

While miRNAs are often reported to strongly repress individual targets, large-scale studies indicate that their regulatory effects are generally modest (< 50%) and that they frequently act by coordinating repression across multiple genes within specific pathways (Bracken et al, 2016). To more comprehensively identify high-confidence miR-342 targets, we applied Exon-Intron Split Analysis (EISA), a computational approach that distinguishes transcriptional from post-transcriptional effects by comparing exonic to intronic read ratios (Gaidatzis et al, 2015). Messenger RNAs post-transcriptionally repressed by miR-342-3p were highly enriched for HITS-CLIP binding sites, validating the utility of EISA for identifying genuine miRNA targets (Fig. EV4G). EISA identified 137 transcripts as post-transcriptionally downregulated by miR-342-3p that also contained a corresponding HITS-CLIP peak (Fig. 3F; Dataset EV4). These mRNAs exhibited an average repression of 55%, with stronger repression observed for transcripts containing 3′UTR binding sites, higher-affinity seed matches (8-mer and 7-mer), and multiple binding sites, which are all hallmarks of canonical miRNA targeting (Figs. 3F and  EV4H). For miR-342-5p, we identified 192 transcripts that were post-transcriptionally repressed and contained a HITS-CLIP peak, with an average repression of 44% (Fig. EV4F; Dataset EV5). Similar to miR-342-3p, stronger repression by miR-342-5p was associated with 3′UTR binding, despite the higher overall frequency of coding region interactions. Comparative analysis of miR-342-3p and miR-342-5p targets revealed only five shared transcripts, indicating that each strand of miR-342 regulates largely distinct sets of genes (Fig. EV4I).

Since miRNAs can repress translation without necessarily reducing mRNA levels, we assessed protein changes using SILAC-based proteomics. Consistent with RNA-seq results, miR-342-3p induced modest changes in protein abundance, with several direct miR-342-3p targets noticeably downregulated at both the transcript and protein level (Fig. 3G,H). These included E2F1 and several E2F pathway members, including RRM2, LMNB1, HMGB2, and DNMT1, as well as CDC42 and its downstream effector PAK2. Repression of selected E2F pathway members and other miR-342-3p 3′UTR targets was further validated by luciferase assays and qPCR following miR-342-3p transfection (Fig. 3I,J). Together, these findings support a model in which miR-342 exerts modest repression across a broad network of target genes, with a potentially coordinated impact on the E2F pathway.

### Collective targeting by miR-342 strongly represses the E2F1 signalling pathway

**Figure 4:**
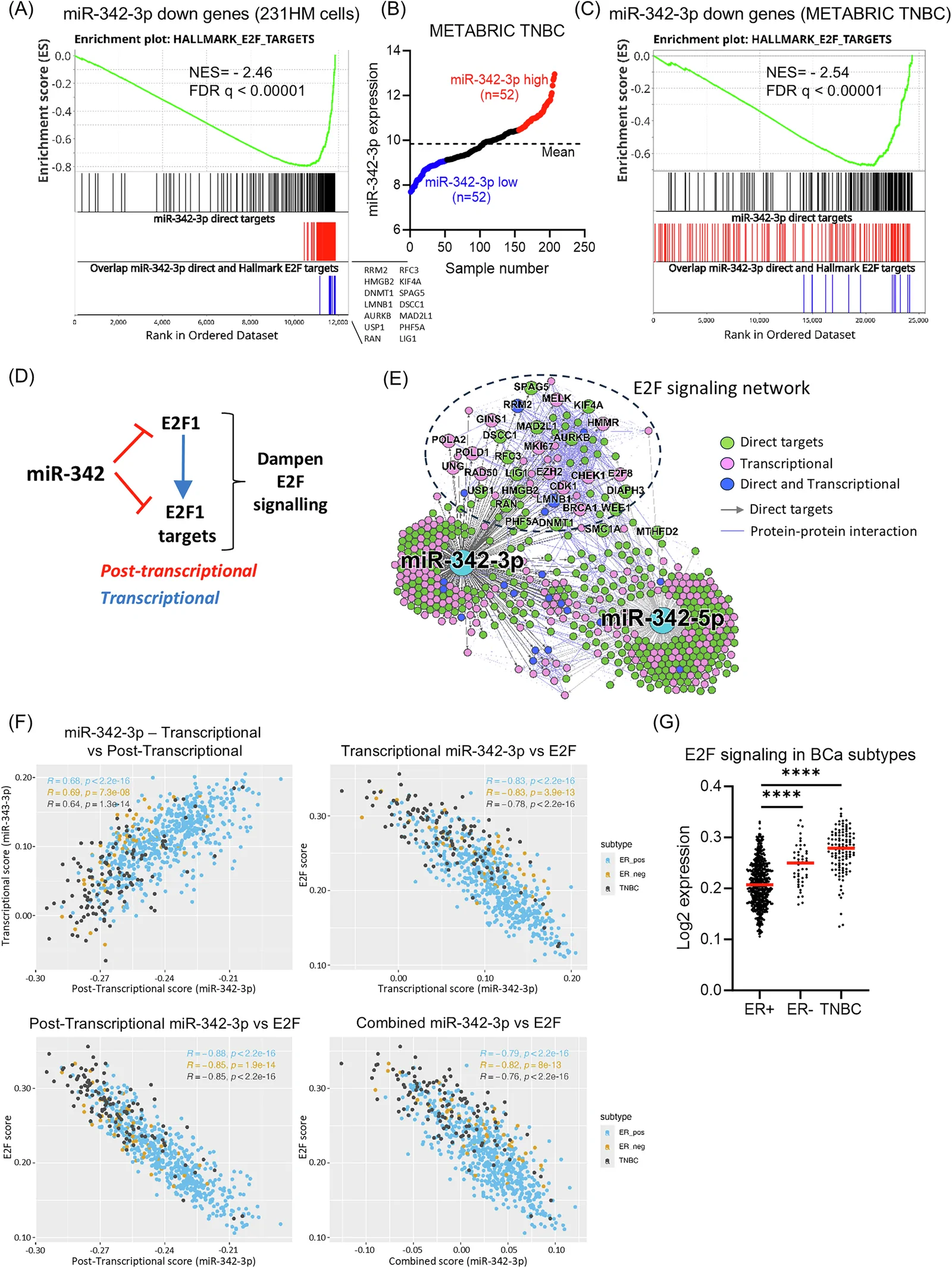
Collective targeting by miR-342 strongly represses the E2F signalling pathway in TNBC. (**A**) Gene set enrichment analysis (GSEA) plot showing negative association of the hallmark E2F signature (200 genes) with miR-342-3p repressed genes in 231HM cells. The rank of miR-342-3p direct targets (red barcode) and overlap between miR-342-3p direct targets and hallmark E2F signature genes (blue barcode) are shown with overlapping genes indicated. (**B**) Ranked plot of TNBC tumours from the METABRIC cohort (*n* = 208) shown from low to high miR-342-3p expression. The lowest and highest quartiles of miR-342-3p expressing tumours are shown in blue and red, respectively. (**C**) GSEA plot showing negative association of the hallmark E2F signature with genes differentially expressed between the highest and lowest quartiles of miR-342-3p expressing METABRIC TNBC tumours. The rank of miR-342-3p direct targets (red barcode) and overlap between miR-342-3p direct targets and hallmark E2F signature genes (blue barcode) are shown. For (**A**, **C**), normalised enrichment scores (NES) and FDR are indicated. (**D**) Schematic of the model where miR-342 dampens E2F signalling by targeting and repressing E2F1 and its target genes. (**E**) Regulon of miR-342-3p and -5p activity showing post-transcriptional (direct, in green) and transcriptional (indirect, in pink) effects of these miRNAs. Genes subject to both post-transcriptional and transcriptional regulation are shown in blue. Genes that are within the hallmark E2F signalling network are named. Grey lines indicate miR-342 regulation and purple lines indicate protein-protein interactions. (**F**) Scatter plots showing correlation of miR-342-3p transcriptional, post-transcriptional, and combined regulons with hallmark E2F score in TCGA breast cancer data, coloured according to ER + , ER-negative and TNBC subtypes. Significance was calculated using Pearson correlation analysis with *P* values shown on the graphs. (**G**) E2F signalling score in ER+ (*n* = 557), ER− (*n* = 48), and TNBC (*n* = 115) breast cancer subtypes in the TCGA data. Significance was calculated using one-way ANOVA with Tukey post hoc test ****P < 0.0001. Source data are available online for this figure.

**Figure EV5:**
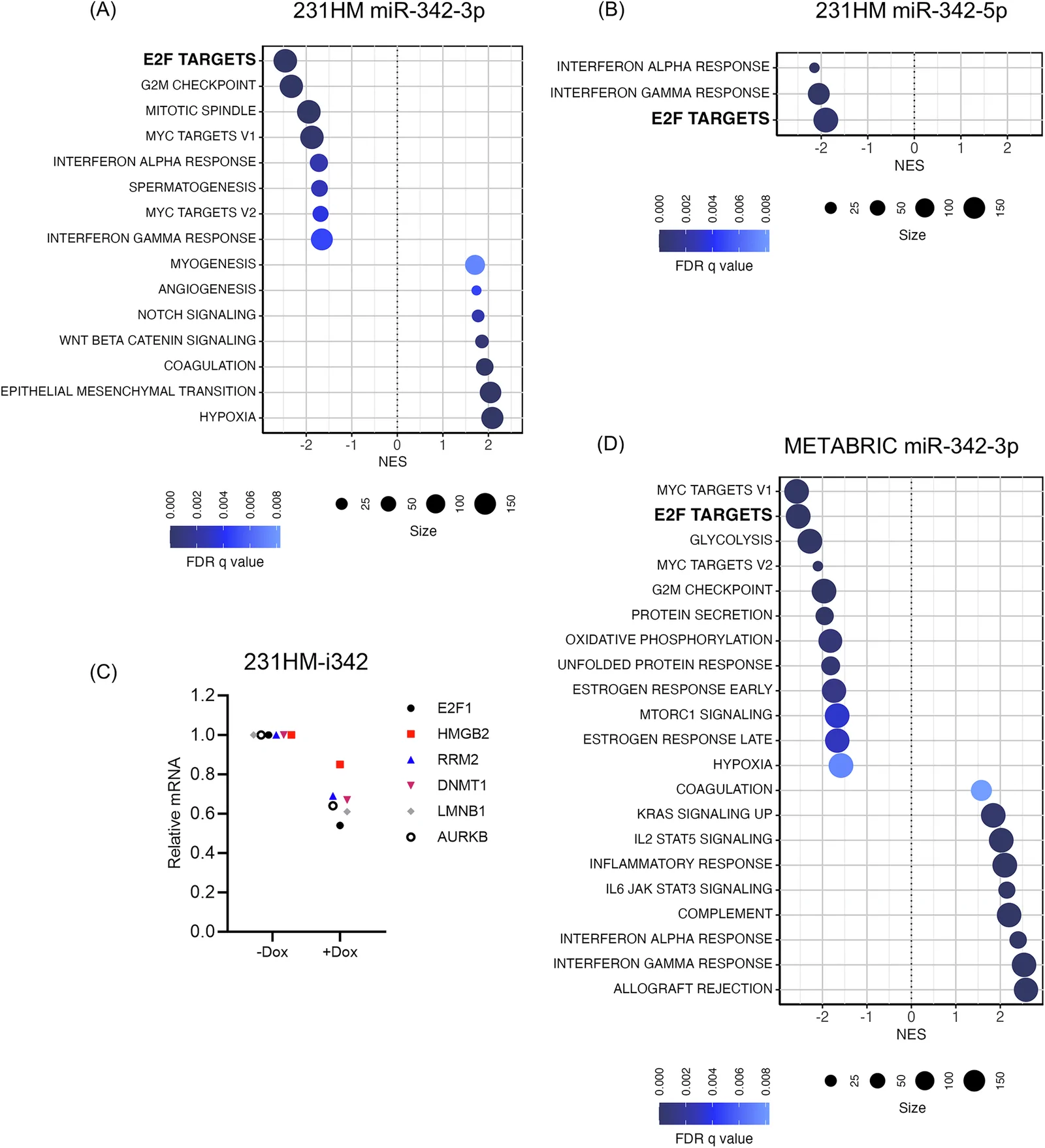
Enrichment of pathways regulated by miR-342-3p and -5p in TNBC. (**A**, **B**) Normalised enrichment scores (NES) and FDR q-values from gene set enrichment analysis (GSEA) of differentially expressed genes following miR-342-3p or miR-342-5p transfection into 231HM cells. Gene sets with FDR < 0.01 are shown. (**C**) Quantitative PCR of E2F pathway genes, which are also direct miR-342-3p target genes, following doxycycline (Dox) induction of 231HM-i342 cells. (**D**) NES and FDR q-values from GSEA of differentially expressed genes in METABRIC TNBC tumours from the top vs bottom quartiles of miR-342-3p expression. Gene sets with FDR < 0.01 are shown.

Although individual miRNA–target interactions often result in modest repression, miRNAs can exert stronger functional effects by coordinately targeting multiple genes within the same pathway (Bracken et al, 2016; Ebert and Sharp, 2012). To identify pathways regulated by miR-342 in an unbiased manner, we performed Gene Set Enrichment Analysis (GSEA) on RNA-seq data from miR-342-transfected cells. Strikingly, we identified very strong repression of the E2F signalling pathway in miR-342-3p-transfected cells (Figs. 4A and EV5A), consistent with our earlier observations (Fig. 3G–J). Interestingly, the E2F pathway was also downregulated following miR-342-5p transfection, suggesting that both mature miR-342 products may cooperate to repress its expression (Fig. EV5B). Multiple genes within the E2F hallmark signature were among the most significantly repressed mRNAs and were directly targeted by miR-342-3p, likely contributing to the robust suppression of this pathway (Fig. 4A). Consistent with these findings, E2F hallmark genes were also downregulated in 231HM-i342 cells following Dox-induced expression of miR-342-3p and -5p (Fig. EV5C).

To assess whether similar regulatory patterns occur in clinical samples, we analysed TNBC tumours from the METABRIC cohort by stratifying them into high and low quartiles of miR-342-3p expression (Fig. 4B), followed by GSEA on differentially expressed genes (Fig. 4C). Consistent with our in vitro findings, the E2F signalling pathway was among the most significantly repressed in tumours with high miR-342-3p expression (Figs. 4C and  EV5D), indicating that miR-342 may influence TNBC progression through suppression of this pathway. Given that miR-342-3p directly targets both *E2F1* and its downstream effectors, the strong repression of E2F signalling likely reflects a combination of transcriptional and post-transcriptional regulation (Fig. 4D).

To investigate this further, we used EISA-derived transcriptional and post-transcriptional scores to construct distinct regulons of miR-342 activity (Dataset EV6). As shown in Fig. 4E, the miR-342 regulon comprises both direct (post-transcriptional) and indirect (transcriptional) targets of miR-342-3p and -5p, including a central E2F signalling node. Applying the miR-342-3p regulon scores to breast cancer samples from TCGA revealed a strong positive correlation between transcriptional and post-transcriptional components, indicating coordinated regulation (Fig. 4F). As expected, regulon scores were lower in TNBC tumours and higher in ER+ tumours, reflecting known differences in miR-342 expression (Fig. EV1E). Correlation analysis between miR-342-3p regulon scores and the E2F signature showed significant and independent negative associations for the transcriptional, post-transcriptional, and the combined miR-342 scores (Fig. 4F). Moreover, E2F pathway activity was elevated in TNBC and ER-negative tumours compared to ER+ tumours, further supporting an inverse relationship between miR-342 levels and E2F pathway activation (Fig. 4G). Collectively, these results indicate that miR-342 orchestrates both transcriptional and post-transcriptional regulatory networks that converge to suppress E2F signalling in breast cancer.

### Pharmacological inhibition of E2F signalling represses metastatic outgrowth of TNBC

The E2F signalling pathway is a major downstream effector of CDK4/6 activity, with inhibitors such as palbociclib suppressing cell cycle progression by blocking CDK4/6-mediated phosphorylation of the retinoblastoma (RB) protein. CDK4/6 inhibitors have shown strong therapeutic benefit in high-risk and metastatic ER+ breast cancer and are now standard-of-care in this setting (Morrison et al, 2024). However, their efficacy in TNBC is limited, potentially due to the molecular heterogeneity of TNBC and the frequent loss of RB in some tumours. Given that miR-342 suppresses metastatic outgrowth in TNBC via repression of E2F signalling, we tested whether the CDK4/6 inhibitor palbociclib could mimic these effects.

**Figure 5:**
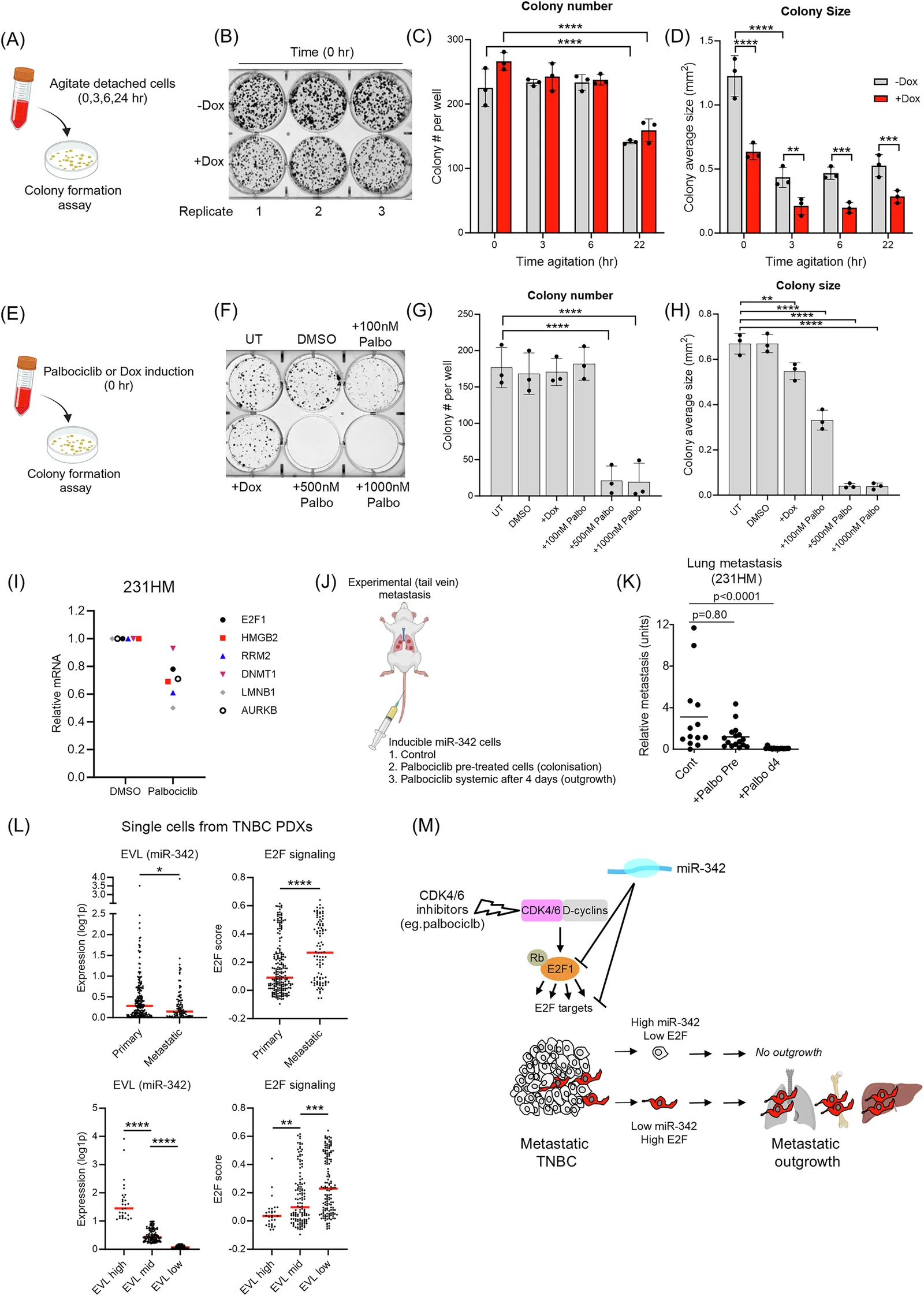
Pharmacological inhibition of the E2F signalling pathway represses metastatic outgrowth of TNBC. (**A**) Schematic of colony outgrowth assay involving cell detachment, agitation for increasing time to simulate anoikis, and limited dilution plating of cells. (**B**) Representative example of colony formation assay. (**C**, **D**) Colony number and size are quantitated with time of cell detachment, and without or with doxycycline treatment to induce miR-342 (harvest on d12). Data are plotted as mean ± SD (*n* = 3). Significance was measured with two-way ANOVA with Bonferroni post hoc tests. *P* values for colony size -Dox vs +Dox day 3 (*P* = 0.0013), -Dox vs +Dox day 6 (*P* = 0.0004), and -Dox vs +Dox day 22 (*P* = 0.0008) and other indicated comparisons are shown as **P < 0.01, ***P < 0.001, ****P < 0.0001. (**E**) Schematic of colony outgrowth assay following treatment with Dox or with increasing doses of palbociclib. (**F**) Representative example of colony formation assay. UT—untreated. (**G**, **H**) Colony number and size are quantitated following the indicated treatments (harvest on d11). Data are plotted as mean ± SD (*n* = 3). Significance was measured with two-way ANOVA with Bonferroni post hoc tests. *P* values for colony size UT vs +Dox (*P* = 0.0054) and other indicated comparisons are shown as **P < 0.01, ****P < 0.0001. (**I**) Quantitative PCR of E2F pathway genes following vehicle (DMSO) or 100 nM palbociclib treatment (cells from colony assay). (**J**) Schematic of intravenous metastasis experiment where 231HM-i342 cells were pre-treated with palbociclib (100 nM) for 2 days or systemically treated with palbociclib (120 mg/kg) daily commencing 4 days post IV injection. (**K**) Lung metastasis measured at experimental endpoint (day 19) by genomic DNA qPCR of human tumour cells (GFP) relative to mouse cells (mouse Vimentin). Significance was calculated by one-way ANOVA with Kruskal–Wallis tests (*n* = 13 for control, *n* = 16 for pre-treatment with palbociclib, and *n* = 15 for palbociclib treatment after tumour cell colonisation). (**L**) MiR-342 host gene EVL and E2F signalling score in single cells of seven matched primary and metastatic samples from TNBC patient-derived xenografts (Winkler et al, 2024). Comparisons are shown between grouped primary (*n* = 204) and metastatic (*n* = 94) cells (top), and of combined primary and metastatic cells binned into high (log1 *P* > 1.0, *n* = 29), mid (log1 *P* 0.2–1.0, *n* = 127) or low (log1 *P* < 0.2, *n* = 142) EVL expression to achieve optimal separation of groups (bottom). Significance was calculated by two-tailed Mann–Whitney test (top) or one-way ANOVA with Kruskal–Wallis tests (bottom). *P* values for EVL in primary vs metastatic (*P* = 0.0256), E2F signalling in EVL high vs EVL mid (*P* = 0.0065), E2F signalling in EVL mid vs EVL low (*P* = 0.0003) and other indicated comparisons are shown as *P < 0.05, **P < 0.01, ***P < 0.001, ****P < 0.0001. (**M**) Model showing the impact of miR-342-E2F network on metastatic outgrowth of TNBC and the ability to inhibit this process using CDK4/6 inhibitors. Source data are available online for this figure.

**Figure EV6:**
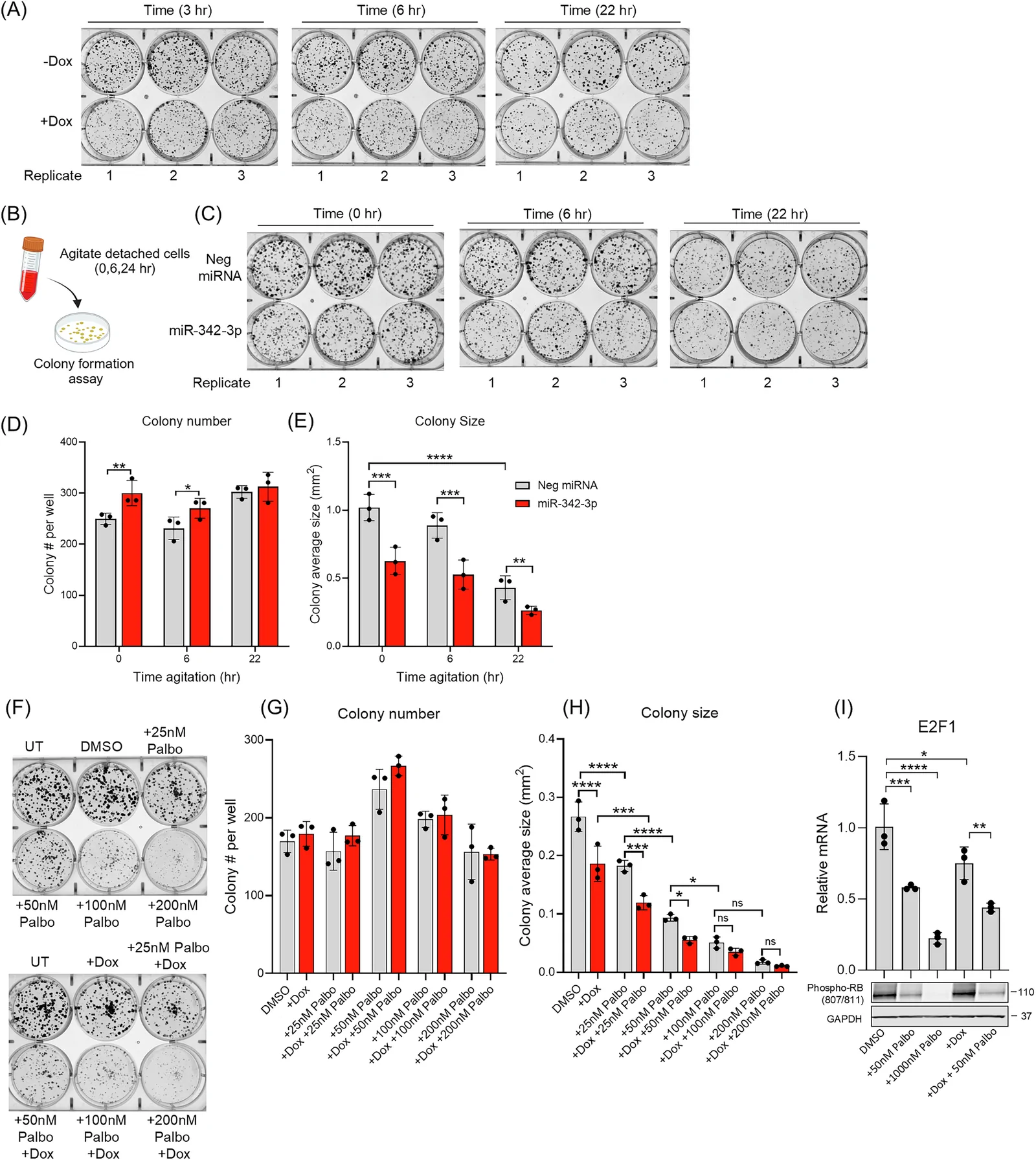
Impact of miR-342 and the CDK4/6 inhibitor palbociclib on colony outgrowth. (**A**) Colony formation assays following doxycycline (Dox) induction of miR-342. The time 0 hr plate is shown in Fig. 5B. Data are quantitated in Fig. 5C,D. (**B**) Schematic of colony outgrowth assay involving cell detachment, agitation for increasing time to simulate anoikis, and limited dilution plating of cells. (**C**) Colony formation assays following control (neg) miRNA or transiently transfected miR-342-3p mimic into 231HM cells. (**D**, **E**) Quantitation of colony number and size with time of cell detachment after transfection of either negative (Neg) control miRNA or the miR-342-3p mimic (analysis on day 11). Data are plotted as mean ± SD (*n* = 3). Significance was measured with two-way ANOVA with Bonferroni post hoc tests. *P* values for colony number for neg miRNA vs miR-342-3p day 0 (*P* = 0.0032), colony number for neg miRNA vs miR-342-3p day 6 (*P* = 0.0120), colony size for neg miRNA vs miR-342-3p day 0 (*P* = 0.0001), colony size for neg miRNA vs miR-342-3p day 6 (*P* = 0.0002), colony size for neg miRNA vs miR-342-3p day 22 (*P* = 0.0099) and other indicated comparisons are shown as *P < 0.05, **P < 0.01, ***P < 0.001, ****P < 0.0001. (**F**) Colony formation assays following individual or co-treatment of 231HM-i342 cells with Dox to induce miR-342 and different doses of palbociclib (Palbo, harvest on d12). UT untreated. (**G**, **H**) Quantitation of colony number and size with the indicated treatments (red bars are co-treatments). Data are plotted as mean ± SD (*n* = 3). Significance was measured with one-way ANOVA with Bonferroni post hoc tests. *P* values for colony size for +Dox vs +Dox +25 nM Palbo (*P* = 0.0001), +25 nM Palbo vs +Dox +25 nM Palbo (*P* = 0.0003), +50 nM Palbo vs +Dox +50 nM Palbo (*P* = 0.0397), +50 nM Palbo vs +100 nM Palbo (*P* = 0.0156) and other indicated comparisons are shown as ns —not significant, *P < 0.05, ***P < 0.001, ****P < 0.0001. (**I**) Quantitative PCR of E2F1 (upper panel) and western blot of phosphorylated RB (lower panel) following treatment of 231HM-i342 cells with palbociclib and/or Dox for 24 h to measure immediate responses to treatment. Data are plotted as mean ± SD (*n* = 3). Significance was measured with one-way ANOVA with Bonferroni post hoc tests. *P* values for E2F1 for DMSO vs +50 nM Palbo (*P* = 0.0010), DMSO vs +Dox (*P* = 0.0321), +Dox vs +Dox +50 nM Palbo (*P* = 0.0100) and other indicated comparisons are shown as *P < 0.05, **P < 0.01, ***P < 0.001, ****P < 0.0001.

To model metastatic outgrowth in vitro, we established a colony formation assay using 231HM-i342 cells subjected to forced suspension and agitation to simulate cell detachment and anoikis stress (Fig. 5A). While extended suspension reduced both colony number and size, indicative of anoikis (Figs. 5B–D and  EV6A), induction of miR-342 did not alter colony number but significantly impaired colony growth at all timepoints. This indicates that miR-342 limits the outgrowth capacity of surviving tumour cells, even those resistant to anoikis (Fig. 5B–D). Similar results were obtained following transient miR-342-3p mimic transfection, supporting its functional role in repressing E2F-driven outgrowth (Fig. EV6B–E).

We next assessed the impact of palbociclib on metastatic outgrowth. As has been reported previously in TNBC cells (Asghar et al, 2017; Chen et al, 2020), high doses of palbociclib (500–1000 nM) can potently inhibit colony formation (Fig. 5E–H). However, at a lower, sub-cytostatic dose (100 nM), palbociclib reduced colony size without affecting colony number, closely phenocopying the effects of miR-342 induction (Fig. 5E–H). As anticipated, palbociclib suppressed the expression of E2F hallmark genes, including several direct targets of miR-342-3p (Fig. 5I). To determine whether palbociclib and miR-342 work cooperatively or independently to influence colony outgrowth, we performed a palbociclib dose-response colony formation assay in the absence or presence of miR-342. As observed previously, neither palbociclib nor Dox treatment affected colony number, however both treatments reduced colony size (Fig. EV6F–H). Co-treatment of lower doses of palbociclib (25 and 50 nM) with Dox led to a significant and additive decrease in colony size relative to the individual treatments alone, while this effect was not as evident at higher doses (100 and 200 nM) due to their stronger impact on colony outgrowth (Fig. EV6F–H). Examination of E2F1 also showed dose-dependent and additive effects on its gene expression consistent with E2F signalling directly impacting colony outgrowth (Fig. EV6I). We also observed a dose-dependent effect of palbociclib in reducing RB phosphorylation, indicating it operates through the canonical RB pathway, while Dox treatment did not affect RB phosphorylation as expected (Fig. EV6I). Collectively, our data demonstrate that palbociclib and miR-342 work cooperatively to additively repress E2F signalling and colony outgrowth.

To examine the effects of palbociclib on metastatic colonisation and outgrowth in vivo, we either pre-treated 231HM cells with 100 nM palbociclib prior to tail vein injection or administered the drug systemically after lung colonisation was established (Fig. 5J). While pre-treatment modestly reduced metastatic outgrowth, systemic administration commencing 4 days after tumour cell inoculation significantly impaired metastatic outgrowth in the lungs over 17 days (Fig. 5K). These findings indicate that CDK4/6 inhibition can effectively limit the outgrowth of metastatic TNBC cells with low levels of miR-342 and elevated E2F signalling.

A recent study performing single-cell RNA-seq data from matched primary and metastatic TNBC patient-derived xenografts (PDX) demonstrated significant heterogeneity in metastatic cell populations, however metastatic tumour cells were generally enriched in E2F pathway expression relative to their primary tumour cell counterparts (Winkler et al, 2024). Analysis of this data revealed the miR-342 host gene EVL was significantly reduced in metastatic tumour cells compared with cells from corresponding primary tumours, and this correlated inversely with E2F pathway expression (Fig. 5L). Segregating all primary and metastatic TNBC cells into high, mid and low levels of EVL further demonstrated that, while there is significant heterogeneity in expression amongst single cells, a reciprocal increase in E2F pathway expression was observed in accordance with decreasing EVL expression (Fig. 5L). These observations in matched primary and metastatic tumours provide further evidence of miR-342-E2F network activity contributing to TNBC metastasis (Fig. 5L). Together, these findings uncover the existence of a miR-342-E2F network that impacts metastatic outgrowth of TNBC and is targetable by CDK4/6 inhibition, highlighting its potential therapeutic utility in TNBCs with low levels of miR-342 and high E2F signalling (Fig. 5M).

## Discussion

A major challenge for the effective treatment of breast cancer is our ability to limit the outgrowth of metastases with their associated morbidity and poor outcome. While hormone-sensitive and HER2-positive breast cancers are amenable to targeted treatments, options for TNBC remain limited and are complicated by its heterogeneous nature and higher metastatic potential. Here, we identify a subset of TNBC characterised by low miR-342/high E2F signalling that promotes metastatic outgrowth and is targetable by CDK4/6 inhibitors. Several miRNAs have been shown to play important regulatory roles in breast cancer metastasis through repression of specific oncogenic factors (Pencheva and Tavazoie, 2013). In general, miRNAs operate by modestly repressing many targets and often co-target multiple members of specific pathways (Bracken et al, 2016; Su et al, 2011), however, comparatively less is known about the spectrum of targets that miRNAs regulate to influence metastasis. We reasoned that identifying the actions of miRNAs that vary with metastatic potential and that correlate with clinical progression of TNBC may reveal pathways that could be targeted to enable more effective treatment of TNBC. In doing so, we uncovered miR-342 as a metastasis suppressor in TNBC and showed that miR-342 controls E2F signalling at transcriptional and post-transcriptional levels by targeting multiple E2F pathway members. Furthermore, we demonstrate that pharmacological inhibition of E2F signalling with the CDK4/6 inhibitor palbociclib effectively reduces metastatic outgrowth of TNBC and implicates a potential therapeutic strategy for TNBC subtypes characterised by low miR-342/high E2F signalling.

Several studies have implicated miR-342 as a favourable prognostic factor in breast cancer and have shown that it can influence tamoxifen sensitivity in ER+ breast cancers (Cittelly et al, 2010; He et al, 2013; Perez**-**Rivas et al, 2014; Young et al, 2017). Consistent with previous reports, we find miR-342 expression is reduced in ERneg and TNBC relative to ER+ breast cancers but is associated with poor prognosis in TNBC and not ER+ breast cancers (Buffa et al, 2011; Lowery et al, 2009; Romero-Cordoba et al, 2018). Despite these associations, the functions of miR-342 in TNBC and particularly in metastatic progression have remained unclear. Using both syngeneic and xenograft models, incorporating different timing of miRNA induction and routes of tumour cell administration, we show that miR-342 specifically suppresses metastatic outgrowth of TNBC cells. While miR-342 repressed primary tumour growth of 4T1.2 cells, it had no effect on the growth of 231HM cells or tumours, indicating cell line-specific or context-dependent activity. Notably, this growth-suppressive effect was not observed in cultured 4T1.2 cells, implying that the in vivo tumour-suppressive effect of miR-342 may depend on interactions with the immune system or tumour microenvironment. Nonetheless, when adjusted for tumour size or when tumours were analysed at equivalent volumes, miR-342 consistently suppressed metastasis across multiple organs. Consistent with this, recent studies in other cancers have implicated tumour and metastasis-suppressive roles for miR-342 involving interactions with tumour-associated macrophages (Bresesti et al, 2025; Gowda et al, 2018). However, as miR-342 suppresses metastasis across distinct immune-competent and deficient TNBC models, and limits colony growth in vitro, our data indicate that miR-342 functions primarily through tumour-cell intrinsic mechanisms to limit the outgrowth of TNBC cells in challenging microenvironments.

To determine whether endogenous miR-342 loss is sufficient to promote metastatic capacity, we inhibited each mature miR-342 species using transient anti-miRs or achieved sustained miR-342 loss through CRISPR-mediated deletion of the miR-342 precursor. Unexpectedly, loss of miR-342-3p markedly impaired the growth of several breast cancer cell lines with low metastatic potential and high endogenous miR-342 expression. Few studies have examined the consequences of miR-342 inhibition in cancer, with an early report finding that miR-342-3p inhibition restored the growth of tamoxifen-treated MCF-7 cells, although its effects in untreated cells were not assessed (Cittelly et al, 2010). MiR-342 knockout mice display context-dependent metabolic and immune-related phenotypes (Fu et al, 2021; Zhang et al, 2021), whereas miR-342 appears dispensable for pancreatic acinar cell tumorigenesis (Dooley et al, 2017), suggesting that its functions are highly cell-type dependent. Our findings indicate that miR-342 is important for the proliferation of non-metastatic breast cancer cells, whereas metastatic cells appear to overcome this growth-inhibitory effect of miR-342 loss through other mechanisms, potentially including activation of E2F signalling. This is consistent with the broad concept that the acquisition of metastatic potential requires multiple genetic and epigenetic alterations and suggests that loss of miR-342 alone is insufficient to confer metastatic potential. However, because miR-342 depletion impaired the growth of miR-342-high, non-metastatic cell lines, we were unable to directly assess whether miR-342 loss alone could enhance metastatic behaviour in these models, representing an important limitation of the study.

Given that miRNAs regulate many target genes, we employed a multi-omics approach to comprehensively define the miR-342 target network in TNBC. This analysis revealed that miR-342-3p directly repressed at least 14 members of the hallmark E2F signalling pathway, including E2F1 itself. Although the repression of individual targets was modest, the collective targeting of E2F signalling components at both the transcriptional and post-transcriptional levels manifested in strong suppression of this pathway. Similar coordination of transcriptional and post-transcriptional responses by a single miRNA has been shown to produce strong cumulative effects on signalling pathways in other biological contexts (Pillman et al, 2019). Furthermore, miR-342-5p, while not directly targeting E2F components, also represses E2F signalling, indicating that the two mature forms of miR-342 act cooperatively. The collective impact of this suppression was evident within breast cancer samples, where we observed inverse associations of both the miR-342 transcriptional and post-transcriptional regulons with E2F activity, but a stronger inverse correlation with the combined miR-342 regulon. While these correlations were strongest in TNBC, they were also evident in other breast cancer subtypes, indicating that miR-342 may act broadly to limit E2F signalling in breast cancer. We also observed that TNBC had elevated E2F signalling relative to the ER+ breast cancers. A similar finding has been reported recently, where increased E2F signalling was also found to correlate with poor prognosis across breast cancer subtypes and was elevated in metastases relative to primary tumours (Oshi et al, 2020). A pro-metastatic role of E2F signalling in breast cancer was also demonstrated in MMTV-PyMT and MMTV-Neu transgenic mice, with metastasis being greatly attenuated following E2F1 deletion (Andrechek, 2015; Hollern et al, 2014; Swiatnicki and Andrechek, 2021). In addition, other studies have shown that miR-342 targeting of E2F1 or the E2F target gene DNMT1 can regulate cancer cell growth in vitro (Lai et al, 2018; Tai et al, 2015; Wang et al, 2011; Weng et al, 2016), indicating a potentially more widespread role of the miR-342-E2F signalling axis in metastatic progression.

While miR-342 exerts strong repression of the E2F signalling pathway, it also targets a range of additional genes that may contribute to its metastasis-suppressing activity. For example, miR-342-3p directly targets the Rho GTPase CDC42 and its downstream effector PAK2, both of which are implicated in TNBC metastasis, and whose pharmacological inhibition has been shown to reduce metastatic spread (Cruz**-**Collazo et al, 2021; Lyu et al, 2024). CDC42 and effector PAK family kinases, including PAK2, have functions in regulating the actin cytoskeleton, cell migration, cell polarity and cell division (Radu et al, 2014). Although miR-342 did not overtly alter cell morphology or migration in our assays, the CDC42–PAK signalling axis plays a broad role in cancer progression and is frequently upregulated in poor-prognosis cancers, including breast cancer (Radu et al, 2014; Xiao et al, 2018). GXYLT1, a glucoside xylosyltransferase, was the most repressed of all miR-342-3p target genes and was recently shown to induce Notch and MAPK signalling to promote colorectal cancer metastasis (Peng et al, 2022). Thus, the additive effect of miR-342 on many target genes, in addition to its strong impact on the E2F pathway, likely contributes to its ability to suppress metastasis. The impact of these other miR-342 targets, as well as the relative contribution of miR-342-3p and miR-342-5p to the metastasis-suppressing activity of miR-342, remains to be determined.

The E2F pathway is the major downstream effector of the cyclin-dependent kinases, CDK4 and CDK6, that phosphorylate the RB protein to release E2F transcription factors and promote cell proliferation. CDK4/6 activity is frequently upregulated in breast cancer through oestrogen receptor signalling and cyclin D1 amplification (Gillett et al, 1994; Kenny et al, 1999). As a result, CDK4/6 inhibitors have demonstrated significant efficacy in ER+ breast cancers and are now widely used for the treatment of hormone receptor-positive, HER2-negative metastatic disease (Morrison et al, 2024). CDK4/6 inhibitors are generally regarded as less effective in TNBCs, due to the higher frequency of RB mutations in TNBC compared to ER+ breast cancers. Despite this, approximately 35-50% of primary TNBC tumours are positive for RB and may theoretically respond to CDK4/6 inhibition (Patel et al, 2020; Trere et al, 2009). However, RB status alone has not been a reliable indicator of metastatic progression in TNBC, with a recent clinical trial so far showing no benefit of CDK4/6 inhibitor monotherapy (Goel et al, 2025). Although combination therapies are being explored in TNBC (Asghar et al, 2017; Fassl et al, 2020; Ge et al, 2020; Nehme et al, 2025; Shu et al, 2020; Teo et al, 2017; Yang et al, 2025), few studies have specifically examined the effects of CDK4/6 inhibitors on metastasis.

Using a highly metastatic RB + TNBC model, we demonstrate that the CDK4/6 inhibitor palbociclib selectively suppresses metastatic outgrowth. These findings are consistent with prior reports (Liu et al, 2017; Saleh et al, 2023), including a study by Liu and colleagues (Liu et al, 2017) that showed that palbociclib inhibited metastasis of MDA-MB-231 tumours and a TNBC patient-derived xenograft, without altering primary tumour growth, an effect linked to reduced Snail expression and impaired cell migration. In our study, both miR-342 induction and palbociclib treatment suppressed the growth of established colonies in vitro and metastatic outgrowth of pre-established lung metastases. While miR-342 did not affect migration or invasion of highly metastatic MDA-MB-231HM cells, both miR-342 upregulation and palbociclib repressed E2F1 and downstream E2F target genes, underscoring the role of E2F signalling in supporting the outgrowth of disseminated tumour cells. Furthermore, co-treatment with both miR-342 and palbociclib additively reduced E2F1 and colony outgrowth, indicating that they may work cooperatively to suppress metastatic outgrowth. However, it remains to be determined whether miR-342 and palbociclib impact cell cycle dynamics or cell survival at metastatic sites. Collectively, our data align with the limited impact of CDK4/6 inhibitors on TNBC cell and primary tumour growth (Nehme et al, 2025; Yang et al, 2025) and indicate that outgrowth of TNBC in metastatic environments may be particularly sensitive to E2F pathway inhibition.

Our analysis of a recent single-cell dataset of matched primary tumour and metastatic TNBC samples (Winkler et al, 2024) revealed that metastatic cells generally have increased E2F signalling and lower miR-342 host gene EVL expression than their primary tumour cell counterparts. Despite the general elevation in E2F signalling in metastatic cells, there was no clear increase in active proliferation or changes in the cell cycle of metastatic vs primary tumour cells (Winkler et al, 2024). This may be due to the inherent cell heterogeneity both within and across tumour and metastatic samples, which is increasingly being recognised and considered a hallmark trait of TNBC (Jovanovic et al, 2023; Lehmann et al, 2011; Lehmann et al, 2021). However, these findings importantly demonstrate the significant transcriptional differences between primary tumour and metastatic cells that could be exploited to target metastatic cell populations. We suggest that identifying tumour populations with low miR-342/high E2F signalling could aid in distinguishing patients who are more likely to develop metastatic disease and where CDK4/6 inhibition may be more therapeutically effective. However, it is important to reiterate that the effectiveness of CDK4/6 inhibitors in TNBC will inevitably be influenced by multiple factors, including RB activity and the levels of upstream pathways (eg. CDK2-Cyclin E) that also impinge on E2F signalling (Asghar et al, 2017). As metastasis is the major cause of TNBC morbidity and mortality, the use of CDK4/6 inhibitors to combat metastatic outgrowth of low miR-342/high E2F signalling TNBC subtypes warrants further exploration. Furthermore, co-treatment with CDK4/6 inhibitors with miR-342 mimics may augment their impacts on E2F signalling and metastatic outgrowth. With the increasing development of resistance to CDK4/6 inhibitors, therapeutic delivery of miR-342 could also potentially suppress pro-metastatic E2F signalling in contexts where CDK4/6 inhibition is no longer effective.

## Methods

**Table:** Reagents and tools table

Reagent/resource	Reference or source	Identifier or catalogue number
**Experimental models**		
Balb/c (*M. musculus*)	WEHI Australia	
NOD.Cg-Prkdcscid Il2rgtm1Wjl/SzJ (NSG) (*M. musculus*)	Animal Resources Centre, Australia	
**Recombinant DNA**		
pLMP-puro-mCherry	Gift from Ross Dickins (Monash)	
pInducer11	Addgene	#44363
psiCheck2	Promega	C8021
**Antibodies**		
Rabbit-anti-E2F1	Bethyl	A300-766A
Rabbit-anti-CDC42	Abcam	ab187643
Mouse-anti-RRM2	Santa Cruz Biotechnology	sc-398294 (A-5)
Rabbit-anti- Phospho-Rb (Ser807/811)	Cell Signaling Technology	#8516 (D20B12)
Mouse-anti-Vimentin	Thermo Fisher Scientific	14-9897 (V9)
Mouse-anti-eIF2C2 (Ago2)	Santa Cruz Biotechnology	sc-53521 (4F9)
Rabbit-anti-GAPDH	Cell Signaling Technology	#5174 (D16H11)
Mouse-anti-Tubulin	Abcam	ab7291
**Oligonucleotides and other sequence-based reagents**		
PCR primers	This study	Table EV1
mirVana miRNA mimic	Ambion	Table EV1
TaqMan MicroRNA assay	Thermo Fisher Scientific	Table EV1
**Chemicals, enzymes and other reagents**		
Doxycycline hyclate	Sigma	D5207
Palbociclib isethionate	MedChemExpress	HY-A0065
Palbociclib	Sigma	PZ0383
D-luciferin	Cayman chemicals	#14681
TRIzol	Thermo Fisher Scientific	15596026
QuantiTect RT kit	Qiagen	205311
QuantiTect SYBR Green reagent	Qiagen	204145
RIPA buffer	Abcam	ab156034
cOmplete™, Mini, EDTA-free Protease Inhibitor Cocktail	Roche	11836170001
PhosSTOP	Roche	4906845001
Bolt™ Bis-Tris Plus Mini Protein Gels	Thermo Fisher Scientific	NW04120
Bolt LDS sample buffer	Thermo Fisher Scientific	B0007
Clarity Western ECL Substrate	Bio-Rad	1705061
Lipofectamine 2000	Thermo Fisher Scientific	11668019
Lipofectamine RNAiMAX	Thermo Fisher Scientific	13778150
Dual Luciferase Reporter Assay System	Promega	E1960
CyQUANT Cell Proliferation Assays	Thermo Fisher Scientific	C7026
Transwell (8.0um pores)	Corning	3422
BioCoat Matrigel Invasion Chambers	Corning	354480
ProLong™ Gold Antifade Mountant	Thermo Fisher Scientific	P36935
L-Lysine-13C6,15N2 hydrochloride	Thermo Fisher Scientific	88209
L-Arginine-HCl, 13C6, 15N4	Thermo Fisher Scientific	89990
SILAC grade DMEM medium	Thermo Fisher Scientific	88364
DMEM medium	Thermo Fisher Scientific	10564011
RPMI 1640 medium	Thermo Fisher Scientific	11875093
AlphaMEM medium	Thermo Fisher Scientific	32561037
**Software**		
FIJI (Image J) software		
Rotor-Gene software	Qiagen	
GraphPad Prism		
**Other**		
Illumina NextSeq 500	Illumina	

### Cell culture, transfection and generation of cell lines

Mouse 4T1 panel cell lines (67NR, 66cl4, 4T1, 4T1.2 and 4T1.13) were a gift from Dr. F Miller at the Karmanos Cancer Institute in Detroit, USA (Aslakson and Miller, 1992). The 4T1.2 and 4T1.13 lines were derived from the 4T1 cells as described previously (Eckhardt et al, 2005). These 4T1 panel mouse lines were each cultured in alpha MEM with 10% FCS and were not externally authenticated. For tumour cell miRNA profiling, 4T1 panel cells were transduced with pMSCV-mCherry and sorted by FACS to generate mCherry-expressing derivatives for tumour cell isolation. To generate 4T1.2 cells with constitutive expression of miR-342, a luciferase-expressing derivative was transduced with retrovirus from the pLMP-miR-342-mCherry vector or the empty vector control and selected with puromycin and enriched by mCherry sorting.

Human breast cancer cell lines were obtained from the American Type Culture Collection (ATCC). MCF7, ZR-75-1, MDA-MB-361, T-47D, Hs578T and MDA-MB-231 cell lines were cultured in DMEM with 10% FCS. BT-549 were cultured in RPMI with 10% FCS. Highly metastatic MDA-MB-231 cells (231HM) were a gift from Prof. ZM Shao at the Fudan University Cancer Institute, Shanghai, China (Chang et al, 2008), were authenticated by short tandem repeat (STR) profiling at the University of Queensland, Australia and cultured in DMEM with 10% FCS. All cell lines were confirmed to be mycoplasma negative prior to use. To generate 231HM cells with constitutive expression of miR-342, cells were transduced using retrovirus from the pLMP-miR-342-mCherry vector or the empty vector control and selected with puromycin and enriched by mCherry sorting. For inducible expression of miR-342, 231HM cells were transduced with lentivirus from pInducer11-miR-342, sorted for GFP expression, and then an individual clone with tightly controlled physiological expression of miR-342 following doxycycline induction was propagated for experiments. Lentivirus and retroviruses were produced in HEK293T cells as previously described (Li et al, 2014; Pillman et al, 2018). For transfection experiments, cells were transfected with 5 nM of miRNA precursors (miRVana miRNA mimics, Ambion) or 100 nM of miRNA inhibitors (miRCURY LNA miRNA inhibitors, Qiagen) using Lipofectamine RNAiMAX (ThermoFisher) prior to downstream functional assays unless otherwise stated. Details of miRNA precursors are provided in Table EV1.

### Cloning of expression and 3’UTR luciferase constructs

The constitutive miR-342 expressing vector was generated by directionally cloning a PCR amplified fragment of the mouse miR-342 pri-miR sequence into the *BglII*/*EcoRI* sites of pLMP-puro-mCherry (Dickins et al, 2005) (a kind gift from A/Prof Ross Dickins, Monash University). The inducible miR-342 expression vector was generated by directionally cloning a PCR amplified fragment of the human miR-342 pri-miR sequence into the XhoI/EcoRI sites of pInducer11 (Meerbrey et al, 2011). The 3’UTR reporter constructs were generated by directionally cloning a PCR amplified fragment of human RRM2 (~1.8 kb), CDC42 (~1.0 kb) or PAK2 (~1.5 kb) 3’UTRs into the *XhoI*/*NotI* sites of psiCheck2 (Promega). Primer sequences used for cloning are provided in Table EV1.

### Mouse experiments and analysis of tumour growth

All mouse experiments were approved by the Austin Health Animal Ethics Committee (A2018/05579) or by SA Pathology Ethics Committee (99c-12), prior to experimentation. Female mice 6–8 weeks of age were purchased from Walter and Eliza Hall Institute, Melbourne, Australia (Balb/c) or from Animal Resources Centre, Perth, Australia (NSG mice - NOD.Cg-Prkdcscid Il2rgtm1Wjl/SzJ). Animals were housed in a clean, temperature-regulated facility and provided food and water *ad libitum*. Experiments commenced when the mice were 7–8 weeks of age. We did not conduct specific randomisation or blinding for mouse studies, although several investigators were involved across different laboratories to minimise potential biases. Tumour cells (1 × 10^5^) of the 4T1 series were injected into the inguinal mammary gland of Balb/c mice, while 1 × 10^6^ MDA-MB-231HM (231HM) cells were injected into the inguinal gland of NSG mice. Tumour volumes were measured using electronic callipers, calculated as 0.5×(length × width^2^). In some experiments, primary tumours were surgically resected at a predetermined volume, and mice continued to be closely monitored for signs of ill-health due to the onset of metastatic disease. In some experiments, lung colonisation and outgrowth were assessed following injection of tumour cells (1 × 10^5^) into the tail vein. Ethical endpoints for euthanasia were as follows: A primary tumour volume of 1500 mm^3^ or less if the tumour was ulcerated, body weight loss of 15% (adjusted for the weight of the tumour), or any signs of ill-health, often due to the development of metastatic lesions in vital organs. At the endpoint, primary tumours, lungs, liver, lymph nodes, spine and femurs were recovered for subsequent analyses.

For miRNA profiling of tumours, 4T1 panel cell lines (67NR, 66cl4, 4T1, 4T1.2 and 4T1.13) were orthotopically implanted into Balb/c mice, and primary tumours were grown for 20 days before disaggregation using collagenase 1, sieve filtration and flow cytometry sorting for mCherry positive cells.

For doxycycline (Dox) treatment of mouse models, Dox (Doxycycline hyclate, Sigma, D5207) was either added to cells for 2 days before administration to mice (1 µg/mL) or provided within the food (600 mg/kg) ad libitum. For palbociclib experiments, palbociclib (palbociclib isethionate, MedChemExpress, HY-A0065) was resuspended in water and either added to cells for 2 days before administration to mice (100 nM) or provided systemically by daily oral gavage at 120 mg/kg/day.

### Measurement of metastatic outgrowth

Metastatic outgrowth was measured either by quantitative PCR of tumour genomic DNA or bioluminescence imaging. For qPCR measurement, whole organs were homogenised and DNA isolated as described previously (Chi et al, 2024). Metastatic outgrowth in various organs recovered at endpoint was measured by multiplexed genomic PCR as described previously by calculating the ratio of mCherry or turboGFP DNA to vimentin DNA (present in all cells) (Chi et al, 2024). For bioluminescence measurement, primary tumours were visualised in situ using the IVIS Spectrum imaging system (PerkinElmer) following intraperitoneal injection of 3 mg per mouse luciferin (D-luciferin, Cayman chemicals, #14681) in PBS and imaging the mouse 15 min later. At the endpoint, primary tumours, lungs, liver, lymph nodes, spine and femurs were recovered and metastasis measured ex vivo by bioluminescence within 30 min of injection of luciferin. Visualisation of metastasis on lungs fixed in 4% paraformaldehyde was completed by hematoxylin and eosin staining or immunohistochemistry using a human-specific Vimentin antibody (V9, 14-9897, Thermo Fisher Scientific).

### CRISPR knockout of miR-342 in cell lines

Human breast cancer cell lines with knockout of the miR-342 precursor sequence were generated using the Alt-R CRISPR-Cas9 system (Integrated DNA Technologies). Guide RNA (gRNA) sequences flanking the miR-342 precursor within intron 3 of the *EVL* gene were designed using the custom Alt-R CRISPR-Cas9 guide RNA design tool. Individual gRNAs were complexed with the Alt-R tracrRNA in duplex buffer, and these complexes were then co-incubated with Cas9 enzyme to generate the Cas9 ribonucleoprotein (RNP) complex according to the manufacturer’s protocol (Integrated DNA Technologies). These Cas9-RNPs were transfected into MDA-MB-231 and MCF-7 cells using Lipofectamine RNAiMAX (ThermoFisher). The efficiency of DNA cleavage and excision of the intervening DNA sequence containing pre-miR-342 was verified in pools of transfected cells by genomic DNA PCR using primers that bind outside the gRNA binding sites. Following verification of successful deletion in cell pools, single clones were generated by cell sorting into 96-well plates with cells grown in a 50:50 mix of fresh and conditioned media. Genomic DNA was extracted from a fraction of single-cell clones using the QuickExtract™ DNA Extraction Solution (Epicentre) and screened by genomic DNA PCR, with PCR products run on 2% agarose gels. MiR-342-3p and -5p levels in wild-type and miR-342 deleted clones were verified by TaqMan microRNA qPCR. The gRNA and genomic DNA PCR primer sequences are provided in Table EV1.

### RNA extraction and quantitative PCR

Total RNA was extracted using TRIzol using the standard manufacturer’s protocol (Thermo Fisher Scientific). For mRNA quantitative PCR, reverse transcription was carried out using the QuantiTect RT kit (Qiagen). Quantitative PCRs were performed in triplicate using the QuantiTect SYBR Green reagent (Qiagen) on a Rotor-Gene 6000 series thermocycler (Qiagen). Analysis was completed using the comparative quantitation feature of the Rotor-Gene software with data normalised to GAPDH expression. MicroRNA qPCR was completed with 5.0 ng of RNA using TaqMan microRNA assay kits according to the manufacturer’s instructions (Applied Biosystems), with data normalised to U6 small nuclear RNA. Primer sequences used for qPCR are provided in Table EV1.

### Protein extraction and western blot

Protein was extracted by lysis of cells in 1× RIPA buffer (Abcam) containing protease (cOmplete Mini, EDTA-free Protease inhibitor Cocktail, Roche) and phosphatase (PhosSTOP EASYpack, Roche) inhibitors. Lysates were fractionated on Bolt Bis-Tris Plus gels (Thermo Fisher Scientific) and transferred to nitrocellulose membranes. Membranes were blocked in 5% skim milk and probed at 4 °C overnight with antibody dilutions indicated in Table EV1. Following incubation with secondary HRP antibodies, membranes were exposed using Clarity western ECL substrate (Bio-Rad) and imaged on a ChemiDoc MP system (Bio-Rad).

### MicroRNA expression profiling

MicroRNA profiling was completed with mCherry sorted cells derived from tumours of the 4T1 panel (67NR, 66cl4, 4T1, 4T1.2 and 4T1.13) using Exiqon miRCURY LNA microarrays (Qiagen, Australia) as described previously (Chi et al, 2022). Briefly, total cell RNA was labelled with Cy3 or Cy5 and co-hybridised to the array in dye-swapping reactions with 67NR tumour cells as a reference. Slides were analysed with a GenePix 4000B Scanner (Molecular Devices).

### Luciferase 3’UTR reporter assays

Reporter assays were completed by co-transfection of 200 ng of psiCheck2 reporter vectors (Promega) with 5 nM of miRVana miRNA mimics (Ambion) into MDA-MB-231HM (231HM) cells using Lipofectamine 2000 (Thermo Fisher Scientific). After 48 h, cells were harvested in 1× passive lysis buffer, and luciferase assays were carried out using the Dual Luciferase Reporter Assay system (Promega) according to the manufacturer’s protocol on a GloMax multi detection system (Promega). Data are expressed as the ratio of *Renilla* luciferase (3’UTR reporter) to Firefly luciferase (constitutive control) and normalised to empty control psiCheck2 vectors.

### Proliferation assays

Proliferation of cells was measured either by direct cell counting with Trypan blue exclusion or using the CyQUANT cell proliferation assay (Thermo Fisher Scientific) according to the manufacturer’s protocol.

### Migration and invasion assays

Migration assays were completed using Transwells (6.5 mm with 8.0-μm pores, Corning). Cells (2 × 10^5^) were plated into Transwells in serum-free medium. For invasion assays, cells (8 × 10^4^) were plated into BioCoat Matrigel invasion chambers (6.5 mm with 8.0-μm pores, Corning) in serum-free medium. Serum-containing medium (10% FCS) was added to the wells beneath each chamber to induce chemotactic migration and invasion. After 4 h for migration or 24 h for invasion, the chamber membranes were fixed by washing in 10% buffered formalin, excised with a scalpel, permeabilised with 0.1% TritonX and mounted onto slides using ProLong Gold Antifade Reagent containing DAPI (Molecular Probes). Cells were imaged on an LSM-700 confocal microscope and counted using FIJI software.

### Colony formation and anoikis assay

To measure colony formation ability following different time periods of cell detachment and agitation (anoikis simulation), cells were resuspended at a concentration of 250 cells/mL in a 15 ml conical tube and placed on a roller at 37 °C for up to 22 h. At different timepoints, cells were added to 6-well plates and incubated for up to 12 days to allow colonies to form. For experiments where miR-342 was induced by doxycycline (Dox) treatment, cells were pre-treated with 1 µg/mL of Dox for 3 days, and Dox was replenished in the medium every 3 days. For miRNA transfection experiments, cells were transiently transfected with 2.5 nM of negative control Pre-miR or miR-342-3p Pre-miR (Ambion) one day prior to cell detachment and agitation. For palbociclib treatment, cells were plated with different doses of palbociclib (Sigma-Aldrich, PZ0383) or DMSO vehicle. At the endpoint, plates containing colonies were fixed with 10% neutral buffered formalin for 30 min, stained with 1% crystal violet for 10 min, and imaged on the Chemidoc MP (Bio-Rad). Colony number and size were enumerated using FIJI software.

### Stable isotope labelling by amino acids in cell culture (SILAC) based proteomics

SILAC was completed on 231HM cells transfected with negative control or miR-342-3p miRVana miRNA mimics (Ambion). 231HM cells were labelled with either light or heavy isotopes of lysine (L-Lysine-^13^C_6_,^15^N_2_ hydrochloride, 52 µg/mL) and arginine (L-Arginine-^13^C_6_,^15^N_4_ hydrochloride, 100 µg/mL) with additional proline (200 µg/mL) in SILAC grade DMEM medium (all reagents from Thermo Fisher Scientific) for four passages prior to transfection. Following labelling, light and heavy labelled cells were each transfected with 20 nM of miRNA mimic using Lipofectamine RNAiMAX (Thermo Fisher Scientific) for 72 h prior to protein harvest. Total protein was harvested by lysing cells in 100 mM Tris pH 7.5, 1% SDS prior to mixing negative control and miR-342-3p-transfected samples, where opposing amino acid isotopes had been incorporated. Protein samples were digested with trypsin and analysed by data-dependent acquisition using high-resolution liquid chromatography-tandem mass spectrometry (LC-MS/MS) on a nanoAcquity system (Waters) at the Walter and Eliza Hall Institute (WEHI) proteomics facility. SILAC ratios were calculated using the MaxQuant algorithm (Cox and Mann, 2008; Cox et al, 2011), and significance was assessed with the Mann–Whitney *U* test.

### RNA sequencing

RNA sequencing (RNA-seq) was completed on 231HM cells transfected with 20 nM miRVana mimic (negative control, miR-342-3p or miR-342-5p, Ambion) using Lipofectamine RNAiMAX (Thermo Fisher). After 72 h, cells were lysed in Trizol (Thermo Fisher) and total RNA extracted according to the manufacturer’s protocol. Total RNA-seq was completed on two biological replicates by first conducting rRNA depletion using Ribo Zero Gold (Illumina) followed by library construction using the KAPA stranded RNA Library Prep kit (Roche). RNA-seq libraries were multiplexed and sequenced on the Illumina NextSeq 500 platform using the stranded 1 ×75 bp protocol by the Australian Cancer Research Foundation (ACRF) Cancer Genomics Facility.

Analysis of RNA-seq data was completed essentially as previously described (Pillman et al, 2018). Briefly, raw reads were adaptor-trimmed, filtered and mapped against the human reference genome (hg19) using TopHat (Kim et al, 2013), generating ~100 million mapped reads per sample. Differential gene expression analysis was completed using Cufflinks (Trapnell et al, 2013) with a *q* value of ≤0.05 considered significant. To segregate transcriptional and post-transcriptional effects of miR-342-3p or miR-342-5p on gene expression, exon intron split analysis (EISA) was employed as described previously (Pillman et al, 2019).

### Argonaute high-throughput sequencing of RNA isolated by crosslinking immunoprecipitation (Ago-HITS-CLIP)

Ago-HITS-CLIP was completed as described in detail previously (Fernandes et al, 2021). Briefly, 231HM cells were transfected with 20 nM miRVana mimic (negative control, miR-342-3p or miR-342-5p) 3 replicates of each; Ambion) using Lipofectamine RNAiMAX (Thermo Fisher). After 48 h, cells were cross-linked using a UV Stratalinker-1800 (Agilent) and lysed in 500 mL of 1× PXL (1× PBS, 0.1% SDS, 0.5% deoxycholate, 0.5% Igepal) + EDTA-free Complete protease inhibitor cocktail (Roche). Following DNA and partial RNA digestion, AGO-RNA complexes were immunoprecipitated using mouse IgA2 monoclonal anti-Ago2 antibody 4F9 (Ikeda et al, 2006) conjugated to protein L Dynabeads (Thermo Fisher Scientific) with a mouse IgA antibody (GeneTex S107) used as a control. AGO-RNA complexes were ligated with RNA linkers on-bead, labelled with ^32^P-ATP, eluted in 1× Bolt LDS sample buffer (Thermo Fisher Scientific) + 1% β-mercaptoethanol, and separated on Bolt 8% Bis-tris Plus gels (Thermo Fisher Scientific). AGO-RNA complexes were then transferred to nitrocellulose, and 115–160 kDa regions (corresponding to RNA tags >30 nt) were excised, followed by proteinase K digestion and RNA extraction using acid phenol (Thermo Fisher Scientific) with 1:1 isopropanol:ethanol precipitation. AGO-bound RNA was pelleted by centrifugation, then separated on a 15% denaturing polyacrylamide gel, where gel slices corresponding to the expected size of the cross-linked RNA were cut out and eluted by the “crush and soak” method as previously described (Jensen and Darnell, 2008).

Sequencing libraries were prepared by reverse transcription and amplification as described in detail previously (Fernandes et al, 2021). Library quality and quantity were assessed by Bioanalyzer (Agilent) and qPCR, pooled and sequenced on an Illumina NextSeq 500 (1×75 bp) at the Australian Cancer Research Foundation (ACRF) Cancer Genomics Facility. Filtered reads were mapped against the human reference genome (hg19) using the TopHat (Kim et al, 2013). Enriched regions of the genome were identified from Samtools quality-filtered alignments (Li et al, 2009) with the MACS2 peak caller (Zhang et al, 2008). The enrichment of motifs within peaks was analysed using Homer (Heinz et al, 2010). Transcripts with a peak score of greater than or equal to 5 (i.e., 5 reads or more above background) were considered as potential miR-342 target genes. Direct targets of miR-342-3p and miR-342-5p were defined as transcripts that were significantly repressed (q < 0.05), showed post-transcriptional repression by EISA, and also contained an Ago-HITS-CLIP peak for the respective miRNA.

### Clinical and cell line data analysis

Expression data for mRNA and miRNA, along with clinical annotations for breast cancer (BRCA) samples from the Cancer Genome Atlas (TCGA) (Cancer Genome Atlas N 2012) and the Molecular Taxonomy of Breast Cancer International Consortium (METABRIC) (Curtis et al, 2012; Dvinge et al, 2013) were retrieved from the GDC data portal or European Genome-Phenome Archive (EGA), respectively. MiRNA expression and clinical data for GSE22220 were obtained from the Gene Expression Omnibus. Kaplan–Meier (KM) curves were plotted based on a median split in ER+ or TNBC breast cancers, with the most significant of miR-342-3p and miR-342-5p presented. For meta-analysis of *EVL* across 26 TNBC cohorts (Gyorffy et al, 2010) the online KM plotter tool was used. Samples were divided into ER + , ER-negative and TNBC subsets based on immunohistochemical (IHC) expression of these markers, with HER2 expression determined by combining HER2 in situ hybridisation data with IHC data to resolve cases where HER2 IHC was not completed for the TCGA data. MiRNA expression in human breast cancer cell lines from the Cancer Cell Line Encyclopedia (CCLE) was downloaded from the DepMap portal. Cell lines were classified into ER + , ER−, and TNBC subsets based on a published data compilation (Dai et al, 2017).

### Gene set enrichment analysis (GSEA)

Gene set enrichment analysis (GSEA) was completed on differential gene analysis data from miR-342-3p or miR-342-5p-transfected 231HM cells or from METABRIC tumours divided into the highest and lowest quartiles of miR-342-3p expression. GSEA utilised the Broad Institute’s online GenePattern platform with the Hallmark signatures collected in the Molecular Signatures Database (MSigDB) (Subramanian et al, 2005).

### Single-sample and single-cell gene set scoring

Single-sample enrichment scores were calculated with custom gene sets comprising core miR-342-3p targets, both post-transcriptionally and transcriptionally regulated, for each TCGA BRCA tumour using the singscore R package (Foroutan et al, 2018) (v1.26.0) with default parameters on centred and scaled gene ranks. The hallmark E2F targets set was obtained from MSigDB (Subramanian et al, 2005) (v7.5). The post-transcriptional miR-342-3p score was composed of the direct miR-342-3p target genes as described above (137 genes). The transcriptional miR-342-3p score was composed of genes that fit the following criteria: a minimum expression in the negative control sample of a FPKM of 3, significant repression by miR-342-3p (q < 0.05), and EISA-defined transcriptional repression (intron < −0.4; yielding 139 genes). Samples were stratified into ER + , ER−, and TNBC subsets as described above. For single cells in seven matched primary and metastatic samples from TNBC patient-derived xenograft models (Winkler et al, 2024), the hallmark E2F targets set was scored from processed data using scanpy.tl.score_genes with default parameters (Wolf et al, 2018). Only cells with non-zero EVL (miR-342 host gene) expression were used in downstream analysis.

### Network visualisation and graphics

Graph visualisation of miR-342 regulatory networks was completed in Gephi using the Force Atlas 2 layout algorithm (Bastian et al, 2009). Custom miR-342-3p and miR-342-5p-regulated gene sets were utilised, comprising the miR-342-3p and miR-342-5p direct target genes (post-transcriptional, 137 and 192 genes, respectively) and EISA-defined transcriptionally repressed gene lists of similar sizes. The criteria for inclusion within the transcriptional gene set were as follows: a minimum expression in the negative control sample of a FPKM of 3, significant repression by miR-342-3p or miR-342-5p (*q* < 0.05), and EISA-defined transcriptional repression (intron for miR-342-3p < -0.4; yielding 139 genes, or for miR-342-5p < -0.65; yielding 121 genes). Schematics in Fig. 1B, 2J, 2M, 2O, 5A, 5E, 5J, and EV6B were created with BioRender.com.

### Statistical analysis

Statistical analyses were performed using GraphPad Prism (v10.0.2) and R software. Individual data points are presented for all graphs with the number of samples, statistical testing and significance values specified in the figure legends. For mouse experiments, a minimum of eight mice were used per group. All other experiments had a minimum of three biological replicates, with all data included for each experiment. *P* values of less than or equal to 0.05 were considered significant.

## Supplementary information

Table EV1

Peer Review File

Dataset EV1

Dataset EV2

Dataset EV3

Dataset EV4

Dataset EV5

Dataset EV6

Source data Fig. 1

Source data Fig. 2

Source data Fig. 3

Source data Fig. 4

Source data Fig. 5

Expanded View Figures

## Data Availability

The RNA-seq and miR-342 HITS-CLIP data have been deposited into the Gene Expression Omnibus with GEO accession numbers GSE307964 and GSE307720, respectively. The source data of this paper are collected in the following database record: biostudies:S-SCDT-10_1038-S44321-026-00496-4.
