# Supplementary material for: Metastasis of triple negative breast cancer is regulated by a targetable miR-342-E2F network
Source: EMBO Mol Med. 2026 Aug 21;18(9):3619–48. doi: 10.1038/s44321-026-00496-4 (PMC13562599; doi:10.1038/s44321-026-00496-4)
Supplement: Supplementary file 1 [file 44321_2026_496_MOESM14_ESM.pdf]

Expanded View Figures

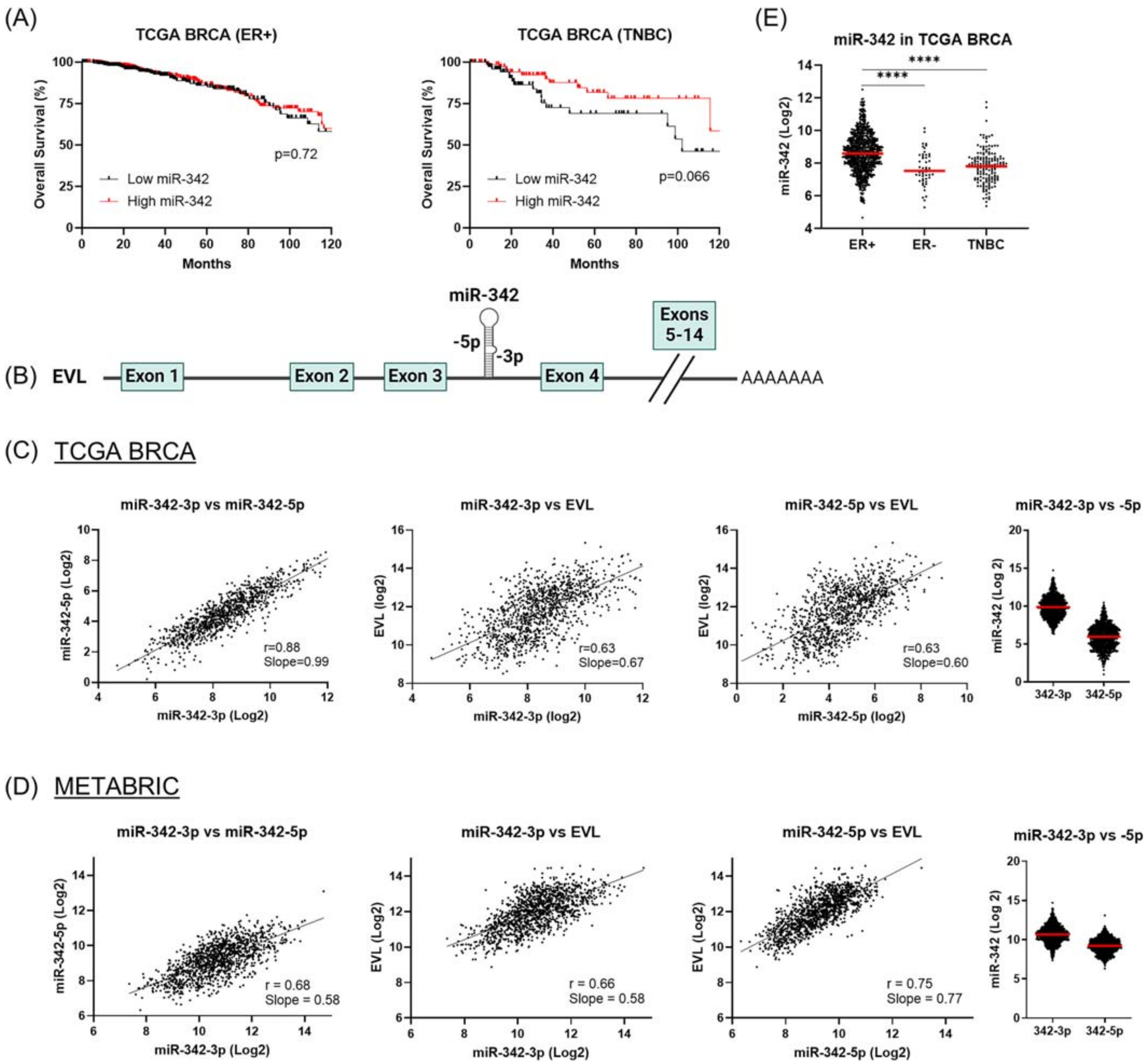

Figure EV1. miR-342 and EVL expression in breast cancer datasets.

(**A**) Kaplan–Meier (KM) overall survival analysis on median split of miR-342 in ER+ (left) and TNBC (right) breast tumours (BRCA) from the TCGA database. Significance was measured by the Log-rank (Mantel–Cox) test. (**B**) Schematic of *EVL* gene locus showing the location of intronic miR-342. (**C**, **D**) Scatter plots showing positive correlations of miR-342-3p, miR-342-5p and EVL in TCGA and METABRIC datasets. Pearson correlation coefficients and associated *P* values are indicated. Relative expression of miR-342-3p and miR-342-5p in TCGA and METABRIC cohorts is shown. (**E**) MiR-342 expression in TCGA breast cancer subsets. Significance was measured by one-way ANOVA with Tukey post hoc test ****$P < 0.0001$.

© The Author(s)

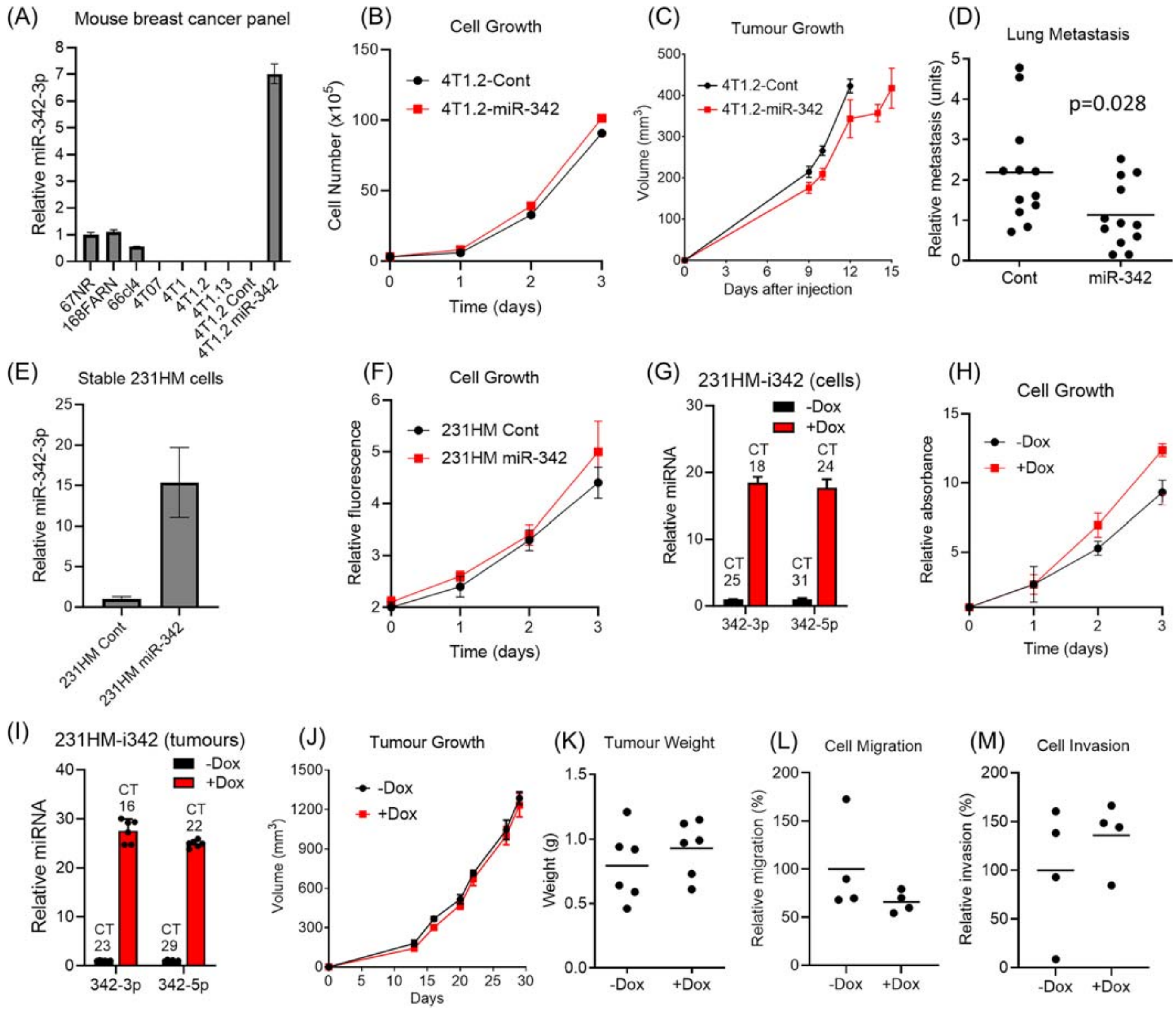

Figure EV2. miR-342 expression and function in mouse and human breast cancer cell lines.

(**A**) Relative expression of miR-342-3p in cells of the 4T1 panel measured by qPCR. The relative level of miR-342-3p in control (empty vector) or miR-342 overexpressing 4T1.2 cells is shown for comparison. Data are plotted as mean ± SD of three technical replicates. (**B**) Proliferation assay of 4T1.2 control and miR-342 overexpressing cell lines. (**C**) Growth of 4T1.2 control and miR-342 overexpressing tumours following mammary fat pad implantation. The experimental endpoint was determined by equivalent tumour volume. Data are plotted as mean ± SEM. $n = 12$ mice/group. (**D**) Lung metastasis measured at experimental endpoint (d12 for Cont; d15 for miR-342) by genomic DNA qPCR of mouse tumour cells (mCherry) relative to total mouse cells (mouse Vimentin), $n = 12$ mice/group. Significance was calculated by a two-tailed Mann–Whitney test. (**E**) Relative expression of miR-342-3p in control (empty vector) or miR-342 overexpressing 231HM cell lines. Data are plotted as mean ± SD of three technical replicates. (**F**) Proliferation assay of 231HM control and miR-342 overexpressing cell lines, as measured by the CyQUANT fluorescence assay. Data are plotted as mean ± SEM ($n = 4$). (**G**) Relative expression of miR-342-3p and -5p in 231HM-i342 cells following 4 days of doxycycline (Dox) induction, as measured by qPCR. Data are plotted as mean ± SD of three technical replicates. Average cycle thresholds (CT) are shown above each bar. (**H**) Growth assay of 231HM-i342 following 4 days of Dox induction (1 day pre-treatment). Mean ± SD ($n = 4$). (**I**) Relative expression of miR-342-3p and -5p in 231HM-i342 tumours at experimental endpoint measured by qPCR (day 30). Mean ± SD ($n = 6$). Average cycle thresholds (CT) are shown above each bar. (**J**) Volumes of 231HM-i342 tumours with or without Dox exposure. Mean ± SEM ($n = 6$). (**K**) Weight of 231HM-i342 tumours at experimental endpoint (day 30). The mean of six tumours per group is shown. Significance was calculated using a two-tailed Mann–Whitney test. (**L**, **M**) Relative cell migration (5 hr) and invasion (24 hr) of 231HM-i342 cells after 4 days of Dox induction. Mean of 4 biological replicates is shown. Significance was calculated by two-tailed Student's *t* test.

© The Author(s)

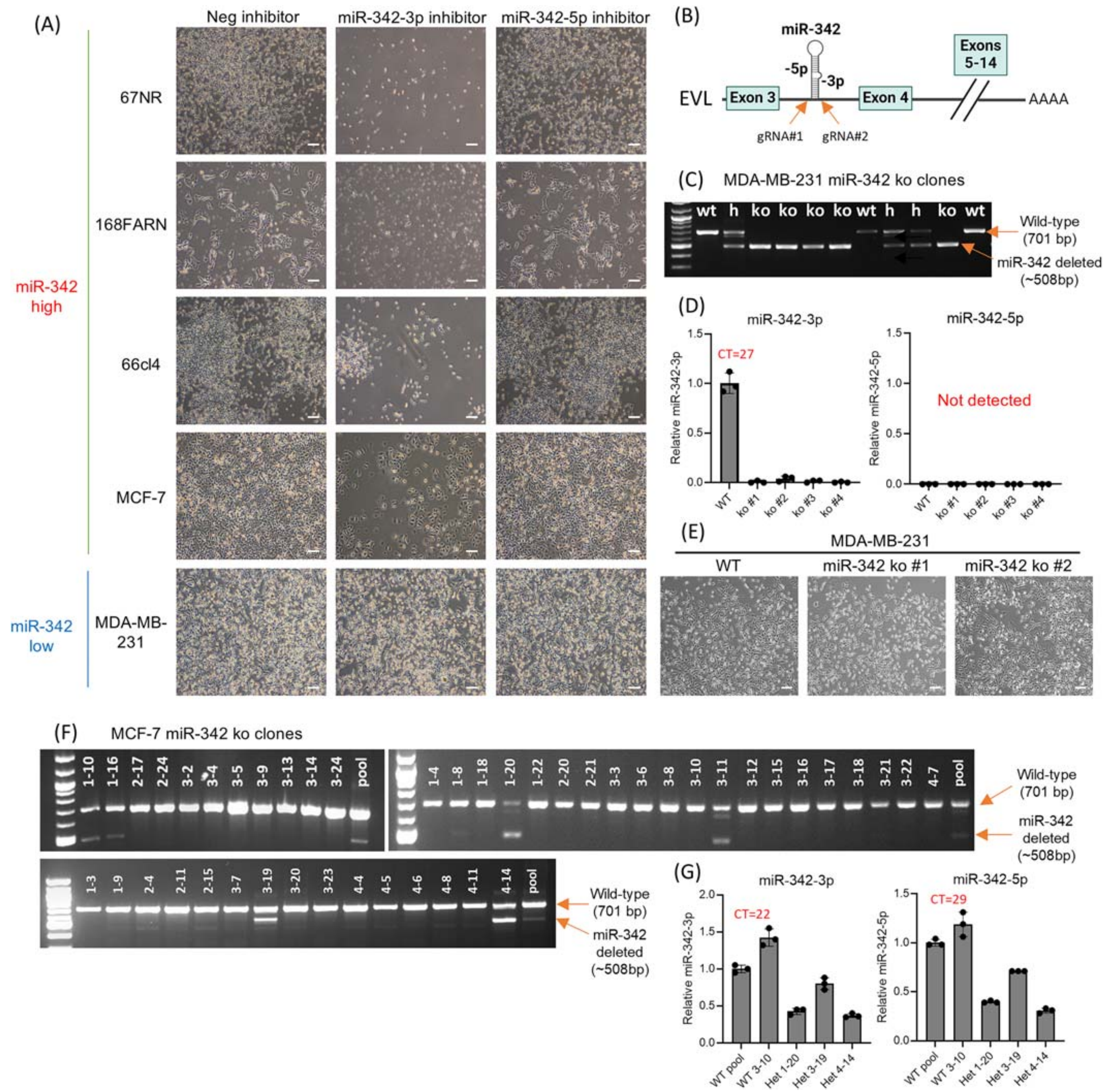

Figure EV3. miR-342 inhibition in mouse and human breast cancer cell lines.

(**A**) Phase contrast images of miR-342 high or miR-342 low expressing mouse and human breast cancer cell lines following 72 h transfection with control, miR-342-3p, or miR-342-5p Anti-miRs. Scale bars represent 50 µm. (**B**) Schematic of miR-342 knockout (ko) approach using dual guide RNAs to cut out pre-miR-342 from the *EVL* host gene. (**C**) Gel showing PCR screen of genomic DNA isolated from MDA-MB-231 miR-342 ko clones (wt wild-type, h heterozygous). The size of the wild-type and full deletion of miR-342 based on the gRNA cut sites is shown. (**D**) Quantitation of miR-342-3p and -5p levels in WT and 4 different miR-342 ko clones. Cycle threshold (CT) of miR-342-3p in WT is shown. (**E**) Phase contrast images of WT and representative miR-342 ko clones. Scale bars represent 50 µm. (**F**) Gels showing PCR screen of genomic DNA isolated from MDA-MB-231 miR-342 ko clones (clone numbers shown above; pool is cells collected after being transfected with gRNA#1 and #2). The size of the wild-type and full deletion of miR-342 based on the gRNA cut sites is shown. (**G**) Quantitation of miR-342-3p and -5p levels in WT pool (parental cells), a WT single clone, and 3 different miR-342 heterozygous clones. The Cycle Threshold (CT) of miR-342-3p and 5p expression in WT pool is shown in red.

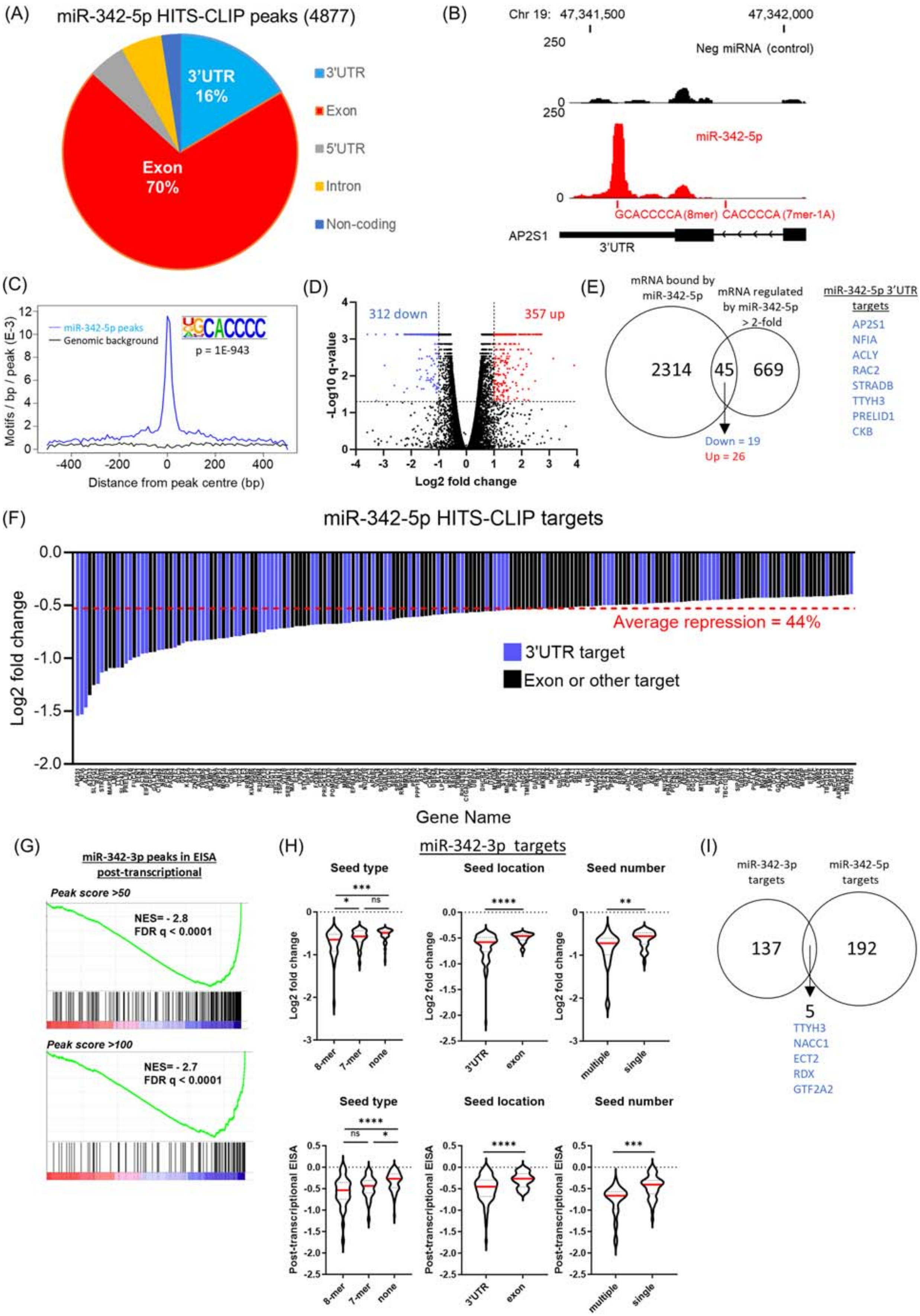
(A)
miR-342-5p HITS-CLIP peaks (4877)
3'UTR 16%
Exon 70%
3'UTR
Exon
5'UTR
Intron
Non-coding
(B)
Chr 19: 47,341,500
47,342,000
250
Neg miRNA (control)
0
250
miR-342-5p
0
GCACCCCA(8mer) CACCCCA(7mer-1A)
AP2S1
3'UTR
(C)
miR-342-5p peaks
Genomic background
p = 1E-943
Motifs / bp / peak (E-3)
Distance from peak centre (bp)
(D)
312 down
357 up
-Log10 q-value
Log2 fold change
(E)
mRNA bound by miR-342-5p
mRNA regulated by miR-342-5p > 2-fold
2314
45
669
Down = 19
Up = 26
miR-342-5p 3'UTR targets
AP2S1
NFIA
ACLY
RAC2
STRADB
TTYH3
PRELID1
CKB
(F)
miR-342-5p HITS-CLIP targets
Log2 fold change
Average repression = 44%
3'UTR target
Exon or other target
Gene Name
(G)
miR-342-3p peaks in EISA post-transcriptional
Peak score >50
NES= - 2.8
FDR q < 0.0001
Peak score >100
NES= - 2.7
FDR q < 0.0001
(H)
miR-342-3p targets
Seed type
Seed location
Seed number
Log2 fold change
8-mer
7-mer
none
3'UTR
exon
multiple
single
Post-transcriptional EISA
(I)
miR-342-3p targets
miR-342-5p targets
137
192
5
TTYH3
NACC1
ECT2
RDX
GTF2A2

© The Author(s)

Figure EV4. Identification of miR-342-5p target genes in TNBC.

(**A**) Pie chart showing the distribution of miR-342-5p Ago-HITS-CLIP peaks in 231HM cells by genomic location type. (**B**) Example of an Ago-HITS-CLIP peak in the AP2S1 gene. MiR-342-5p seed recognition sequences in the AP2S1 3′UTR are shown, with Ago-loaded miR-342-5p binding only to the 3′UTR binding site. Genome tracks represent the average read density of all replicates for each condition. (**C**) Distribution of miR-342-5p recognition sequences within Ago-HITS-CLIP peaks. The most enriched motif, corresponding to miR-342-5p, and the associated *P* value are shown. Background represents the occurrence of the motif on the opposite strand of the peak. (**D**) Volcano plot showing expression of genes altered by miR-342-5p transfection in 231HM cells ($n = 2$ biological replicates). Blue and red dots indicate significantly downregulated and upregulated genes, respectively, changing >twofold (*q* value < 0.05). (**E**) Venn diagram showing overlap of mRNAs significantly regulated by miR-342-5p with those having a miR-342-5p HITS-CLIP peak. For the overlapping genes, those that are repressed and contain 3′UTR binding sites are indicated. (**F**) Ranked bar chart showing change in expression of high-confidence miR-342-5p target genes upon miR-342-5p transfection. The location of the miR-342-3p HITS-CLIP is indicated by colour, with the dotted line marking the average degree of repression across all mRNAs. (**G**) Gene set enrichment analysis (GSEA) demonstrating increasing repression of miR-342-5p HITS-CLIP targets with a peak score of >50 or >100. Normalised enrichment scores (NES) and FDR are indicated. (**H**) Violin plots showing the degree of total repression (upper) and post-transcriptional repression (lower) for mRNA with different miR-342-3p seed types, locations and numbers. Significance was measured by one-way ANOVA with Tukey post hoc test (3 groups) or unpaired *t* test (2 groups). *P* values for gene expression for seed type 8-mer vs 7-mer ($P = 0.0383$), seed type 8-mer vs none ($P = 0.0001$), seed number multiple vs single ($P = 0.0033$) and other indicated comparisons are shown as ns—not significant, *$P < 0.05$, **$P < 0.01$, ***$P < 0.001$, ****$P < 0.0001$. *P* values for post-transcriptional expression for seed type 7-mer vs none ($P = 0.0456$), seed number multiple vs single ($P = 0.0003$) and other indicated comparisons are shown as ns—not significant, *$P < 0.05$, ***$P < 0.001$, ****$P < 0.0001$. (**I**) Venn diagram showing overlap between miR-342-3p and -5p direct targets. The overlapping genes are indicated.

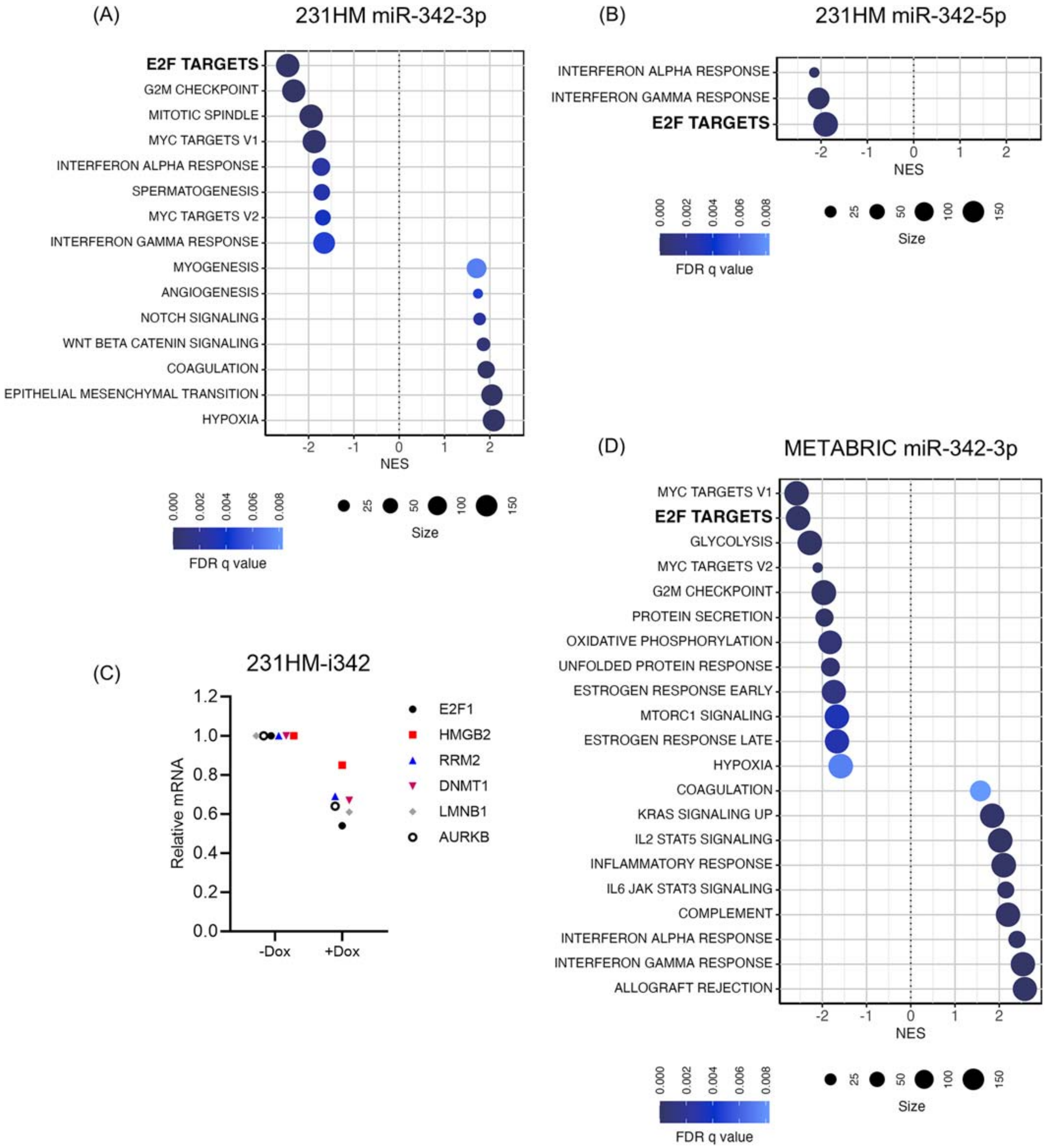

Figure EV5. Enrichment of pathways regulated by miR-342-3p and -5p in TNBC.

(**A**, **B**) Normalised enrichment scores (NES) and FDR q-values from gene set enrichment analysis (GSEA) of differentially expressed genes following miR-342-3p or miR-342-5p transfection into 231HM cells. Gene sets with FDR < 0.01 are shown. (**C**) Quantitative PCR of E2F pathway genes, which are also direct miR-342-3p target genes, following doxycycline (Dox) induction of 231HM-i342 cells. (**D**) NES and FDR q-values from GSEA of differentially expressed genes in METABRIC TNBC tumours from the top vs bottom quartiles of miR-342-3p expression. Gene sets with FDR < 0.01 are shown.

© The Author(s)

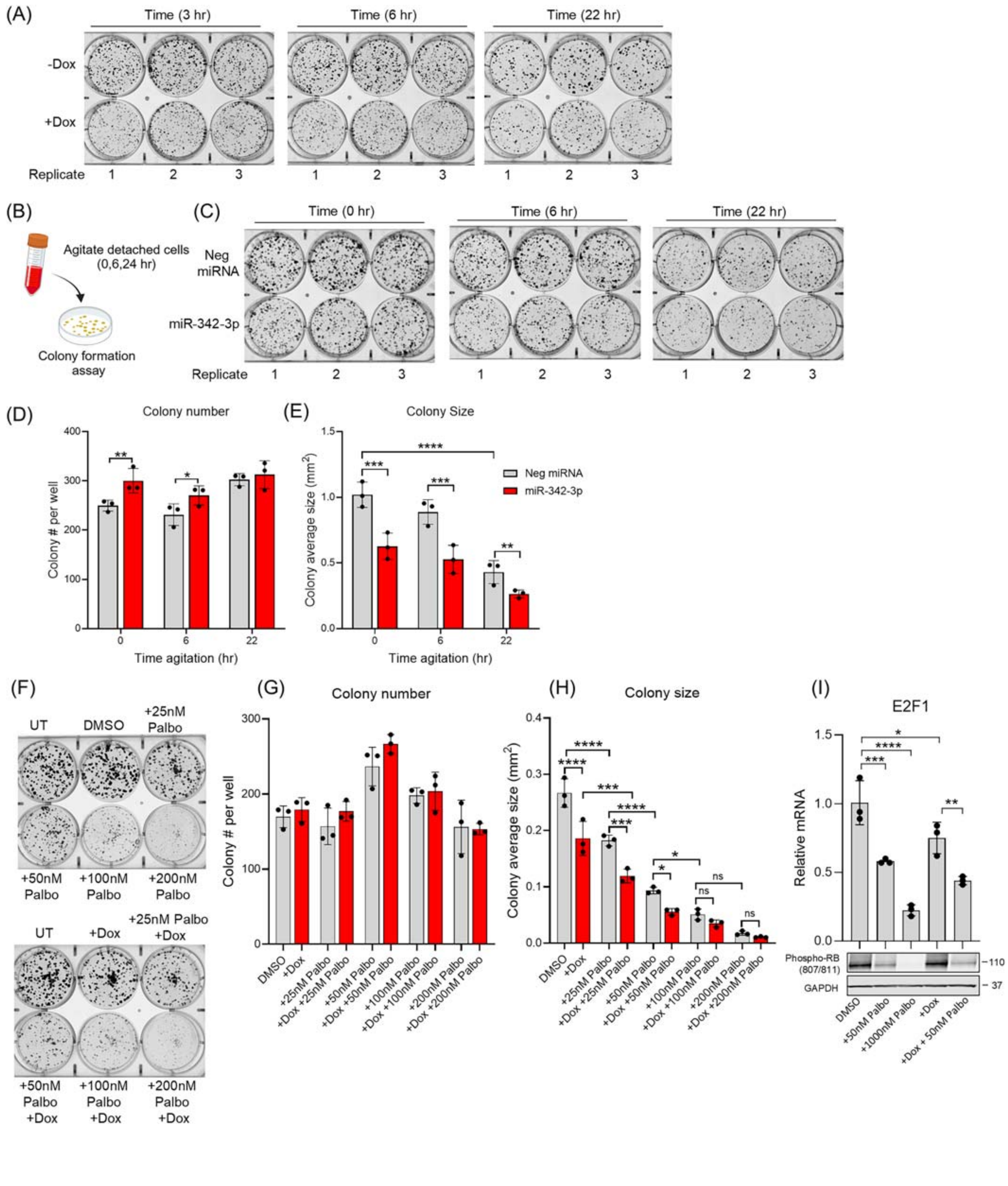

(A)
Time (3 hr)
Time (6 hr)
Time (22 hr)
-Dox
+Dox
Replicate 1 2 3
(B)
Agitate detached cells (0,6,24 hr)
Colony formation assay
(C)
Time (0 hr)
Time (6 hr)
Time (22 hr)
Neg miRNA
miR-342-3p
Replicate 1 2 3
(D)
Colony number
Colony # per well
Time agitation (hr)
(E)
Colony Size
Colony average size (mm2)
Neg miRNA
miR-342-3p
Time agitation (hr)
(F)
UT
DMSO
+25nM Palbo
+50nM Palbo
+100nM Palbo
+200nM Palbo
+Dox
+25nM Palbo +Dox
+50nM Palbo +Dox
+100nM Palbo +Dox
+200nM Palbo +Dox
(G)
Colony number
Colony # per well
(H)
Colony size
Colony average size (mm2)
(I)
E2F1
Relative mRNA
Phospho-RB (807/811)
GAPDH
-110
-37
DMSO
+50nM Palbo
+1000nM Palbo
+Dox
+Dox + 50nM Palbo

Figure EV6. Impact of miR-342 and the CDK4/6 inhibitor palbociclib on colony outgrowth.

(**A**) Colony formation assays following doxycycline (Dox) induction of miR-342. The time 0 hr plate is shown in Fig. 5B. Data are quantitated in Fig. 5C,D. (**B**) Schematic of colony outgrowth assay involving cell detachment, agitation for increasing time to simulate anoikis, and limited dilution plating of cells. (**C**) Colony formation assays following control (neg) miRNA or transiently transfected miR-342-3p mimic into 231HM cells. (**D**, **E**) Quantitation of colony number and size with time of cell detachment after transfection of either negative (Neg) control miRNA or the miR-342-3p mimic (analysis on day 11). Data are plotted as mean ± SD ($n = 3$). Significance was measured with two-way ANOVA with Bonferroni post hoc tests. P values for colony number for neg miRNA vs miR-342-3p day 0 ($P = 0.0032$), colony number for neg miRNA vs miR-342-3p day 6 ($P = 0.0120$), colony size for neg miRNA vs miR-342-3p day 0 ($P = 0.0001$), colony size for neg miRNA vs miR-342-3p day 6 ($P = 0.0002$), colony size for neg miRNA vs miR-342-3p day 22 ($P = 0.0099$) and other indicated comparisons are shown as $^*P < 0.05$, $^{**}P < 0.01$, $^{***}P < 0.001$, $^{****}P < 0.0001$. (**F**) Colony formation assays following individual or co-treatment of 231HM-i342 cells with Dox to induce miR-342 and different doses of palbociclib (Palbo, harvest on d12). UT untreated. (**G**, **H**) Quantitation of colony number and size with the indicated treatments (red bars are co-treatments). Data are plotted as mean ± SD ($n = 3$). Significance was measured with one-way ANOVA with Bonferroni post hoc tests. P values for colony size for +Dox vs +Dox +25 nM Palbo ($P = 0.0001$), +25 nM Palbo vs +Dox +25 nM Palbo ($P = 0.0003$), +50 nM Palbo vs +Dox +50 nM Palbo ($P = 0.0397$), +50 nM Palbo vs +100 nM Palbo ($P = 0.0156$) and other indicated comparisons are shown as ns —not significant, $^*P < 0.05$, $^{***}P < 0.001$, $^{****}P < 0.0001$. (**I**) Quantitative PCR of E2F1 (upper panel) and western blot of phosphorylated RB (lower panel) following treatment of 231HM-i342 cells with palbociclib and/or Dox for 24 h to measure immediate responses to treatment. Data are plotted as mean ± SD ($n = 3$). Significance was measured with one-way ANOVA with Bonferroni post hoc tests. P values for E2F1 for DMSO vs +50 nM Palbo ($P = 0.0010$), DMSO vs +Dox ($P = 0.0321$), +Dox vs +Dox +50 nM Palbo ($P = 0.0100$) and other indicated comparisons are shown as $^*P < 0.05$, $^{**}P < 0.01$, $^{***}P < 0.001$, $^{****}P < 0.0001$.

 © The Author(s)
