# Supplementary material for: Metastasis of triple negative breast cancer is regulated by a targetable miR-342-E2F network
Source: EMBO Mol Med. 2026 Aug 21;18(9):3619–48. doi: 10.1038/s44321-026-00496-4 (PMC13562599; doi:10.1038/s44321-026-00496-4)
Supplement: Supplementary file 2 [file 44321_2026_496_MOESM2_ESM.pdf]

EMBO Molecular Medicine

Metastasis of triple negative breast cancer is regulated by a targetable miR-342-E2F network

Victoria Arnet, Cameron Johnstone, Richard Redvers, Caroline Chambers, Katherine Pillman, John Toubia, Rachael Lumb, B Dredge, Andrew Bert, Emily Hackett-Jones, Pannapa Pinweha, Julie Bracken, Shruti Deshpande, Zoe Price, Michael Ortiz, Kaitlin Scheer, Jasleen Rajpal, Ashleigh Geiger, Suraya Roslan, Xiaochun Li, Sandra O'Toole, Traude Beilharz, Cameron Bracken, Yeesim Khew-Goodall, Gregory Goodall, Robin Anderson, and Philip Gregory

Corresponding authors: Philip Gregory (philip.gregory@adelaide.edu.au) , Robin Anderson (Robin.anderson@onjcri.org.au)

Review Timeline:

Submission Date:	20th Nov 25
Editorial Decision:	19th Dec 25
Revision Received:	26th Mar 26
Editorial Decision:	27th Apr 26
Appeal:	12th May 26
Editor's Correspondence:	27th May 26
Revision Received:	24th Jun 26
Editorial Decision:	9th Jul 26
Revision Received:	14th Jul 26
Accepted:	20th Jul 26

Editor: Lise Roth

Transaction Report:

(Note: With the exception of the correction of typographical or spelling errors that could be a source of ambiguity, letters and reports are not edited. Depending on transfer agreements, referee reports obtained elsewhere may or may not be included in this compilation. Referee reports are anonymous unless the Referee chooses to sign their reports.)

19th Dec 2025

Dear Dr. Gregory,

Thank you for submitting your manuscript to EMBO Molecular Medicine. We have now received two reports. Referee #3 has not yet responded, but as both referees #1 and #2 make similar recommendations, we would like to make a decision now to avoid further delay in the process. If referee #3 provides a report, we will send it to you on the understanding that we will not ask you to carry out any extensive experiments beyond those requested in the attached reports from referees #1 and #2. As you will see from the reports below, the referees recognize the interest of the study and generally support the publication of your work, provided appropriate revisions.

Addressing the reviewers' concerns in full will be necessary for further considering the manuscript in our journal, and acceptance of the manuscript will entail a second round of review. EMBO Molecular Medicine encourages a single round of revision only and therefore, acceptance or rejection of the manuscript will depend on the completeness of your responses included in the next, final version of the manuscript. For this reason, and to save you frustration at the end, I would strongly discourage you from returning an incomplete revision.

If you would like to discuss further the points raised by the referees, I am available to do so via email or video. Let me know if you are interested in this option.

We are expecting your revised manuscript within three to four months, if you anticipate any delay, please contact us.

When preparing your revised manuscript, please refer to our guidelines: https://link.springer.com/journal/44321/submission-guidelines#cms-Revised-submissions. We perform an initial quality control of all revised manuscripts before re-review; failure to include requested items will delay the evaluation of your revision.

We require:

1) A .docx formatted version of the manuscript text (including legends for main figures, EV figures and tables). Please make sure that the changes are highlighted to be clearly visible.

2) Individual production quality figure files as .eps, .tif, .jpg (one file per figure). For guidance, download the 'Figure Guide PDF': https://media.springernature.com/original/springer-cms/rest/v1/content/27825798/data/v1.

3) A .docx formatted letter INCLUDING the reviewers' reports and your detailed point-by-point responses to their comments. As part of the EMBO Press transparent editorial process, the point-by-point response is part of the Review Process File (RPF), which will be published alongside your paper.

4) A complete author checklist, which you can download from our author guidelines. Please insert information in the checklist that is also reflected in the manuscript. The completed author checklist will also be part of the RPF.

5) Please note that all corresponding authors are required to supply an ORCID ID for their name upon submission of a revised manuscript.

6) It is mandatory to include a 'Data Availability' section after the Materials and Methods. Before submitting your revision, primary datasets produced in this study need to be deposited in an appropriate public database, and the accession numbers and database listed under 'Data Availability'. Please remember to provide a reviewer password if the datasets are not yet public.

In case you have no data that requires deposition in a public database, please state so in this section. Note that the Data Availability Section is restricted to new primary data that are part of this study.

7) For data quantification: please specify the name of the statistical test used to generate error bars and P values, the number (n) of independent experiments (specify technical or biological replicates) underlying each data point and the test used to calculate p-values in each figure legend. The figure legends should contain a basic description of n, P and the test applied. Graphs must include a description of the bars and the error bars (s.d., s.e.m.).

8) At EMBO Press we ask authors to provide source data for the main manuscript figures. You will receive a separate email with instructions for providing source data with your revised manuscript, including how to upload and organize the files.

9) Our journal encourages inclusion of *data citations in the reference list* to directly cite datasets that were re-used and

obtained from public databases. Data citations in the article text are distinct from normal bibliographical citations and should directly link to the database records from which the data can be accessed. In the main text, data citations are formatted as follows: "Data ref: Smith et al, 2001" or "Data ref: NCBI Sequence Read Archive PRJNA342805, 2017". In the Reference list, data citations must be labeled with "[DATASET]". A data reference must provide the database name, accession number/identifiers and a resolvable link to the landing page from which the data can be accessed at the end of the reference.

10) We replaced Supplementary Information with Expanded View (EV) Figures and Tables that are collapsible/expandable online. A maximum of 5 EV Figures can be typeset. EV Figures should be cited as 'Figure EV1, Figure EV2" etc... in the text and their respective legends should be included in the main text after the legends of regular figures.

- For the figures that you do NOT wish to display as Expanded View figures, they should be bundled together with their legends in a single PDF file called *Appendix*, which should start with a short Table of Content. Appendix figures should be referred to in the main text as: "Appendix Figure S1, Appendix Figure S2" etc.

- Additional Tables/Datasets should be labeled and referred to as Table EV1, Dataset EV1, etc. Legends have to be provided in a separate tab in case of .xls files. Alternatively, the legend can be supplied as a separate text file (README) and zipped together with the Table/Dataset file.

11) The paper explained: EMBO Molecular Medicine articles are accompanied by a summary of the articles to emphasize the major findings in the paper and their medical implications for the non-specialist reader. Please provide a draft summary of your article highlighting
- the medical issue you are addressing,
- the results obtained and
- their clinical impact.

This may be edited to ensure that readers understand the significance and context of the research. Please refer to any of our published articles for an example.

12) Author contributions: You will be asked to provide CRediT (Contributor Role Taxonomy) terms in the submission system. These replace a narrative author contribution section in the manuscript.

13) A Conflict of Interest statement should be provided in the main text.

14) Every published paper includes a 'Synopsis' to further enhance discoverability. Synopses are displayed on the journal webpage and are freely accessible to all readers. They include a short stand first (maximum of 300 characters, including space) as well as 2-5 one-sentences bullet points that summarizes the paper. Please write the bullet points to summarize the key NEW findings. They should be designed to be complementary to the abstract - i.e. not repeat the same text. We encourage inclusion of key acronyms and quantitative information (maximum of 30 words / bullet point). Please use the passive voice. Please attach these in a separate file or send them by email, we will incorporate them accordingly.

Please also suggest a striking image or visual abstract to illustrate your article as a PNG file 550 px wide x 300-600 px high.

15) Include a Reagents and Tools Table as part of the Methods section, which can be downloaded from our author guidelines.

16) As part of the EMBO Publications transparent editorial process initiative (see our Editorial at http://embomolmed.embopress.org/content/2/9/329), EMBO Molecular Medicine will publish online a Review Process File (RPF) to accompany accepted manuscripts.
In the event of acceptance, this file will be published in conjunction with your paper and will include the anonymous referee reports, your point-by-point response and all pertinent correspondence relating to the manuscript. Let us know whether you agree with the publication of the RPF and as here, if you want to remove or not any figures from it prior to publication.
Please note that the Authors checklist will be published at the end of the RPF.

EMBO Molecular Medicine has a "scooping protection" policy, whereby similar findings that are published by others during review or revision are not a criterion for rejection. Should you decide to submit a revised version, I do ask that you get in touch after three months if you have not completed it, to update us on the status.

I look forward to receiving your revised manuscript.

Yours sincerely,

Lise Roth

Lise Roth, PhD
Senior Editor
EMBO Molecular Medicine

***** Reviewer's comments *****

Referee #1 (Comments on Novelty/Model System for Author):

The technical quality of the presented findings is high, however, there are some potential issues with the experimental setup. The authors should downregulate the miRNA in some cell line system (e.g., 67NR) to verify that the miRNA and not its non-physiological levels induce the observed phenotypes (see my comments to the authors).
The medical impact might be high, if the findings can be confirmed in a downregulation setting.
The 67NR cell line could be a more adequate model system.

Referee #1 (Remarks for Author):

Arnet and co-workers analyzed the role of miR-343 in the context of metastasis in triple negative breast cancer. In a panel of TNBC tumor cell lines differing also in their potential to metastasize, they had identified miR-342 (among others) as strongly repressed in the metastasizing cell lines. To identify targets and phenotypes of this miRNA, they overexpressed miR-342 in the highly metastatic 4T1.2 cell line, sequenced the mRNA, performed some phenotypic assays and established in vivo metastasis models. Correlation of miR-342-expression with survival was established using sequencing (TCGA, METABRIC) and microarray datasets. Collectively, this led to the identification of a transcriptional network centering on E2F signaling and cell cycle regulation at G1/S transition. In vivo data in their syngeneic model suggested that overexpression of miR-342 could reduce formation of metastases, while no dramatic effects were observed on tumor growth. Consequently, they blocked CDK4/6 and observed that inhibition of these kinases abrogated metastasis potential of the cells similar as overexpression of miR-342 did. Furthermore, they observe in publicly available single cell RNA-seq data that E2F signaling and expression of the host gene of miR-342 is inversely correlated with metastasis formation. The authors conclude that they have identified a novel axis involving miR-342 and E2F as driver of metastasis formation in TNBC, potentially opening new perspectives for treatment of a subgroup of TNBCs that are wildtype for RB1, and to potentially prevent formation of metastases. This study touches a highly relevant clinical problem and brings up an interesting mechanism that might be targeted for the benefit of a subset of breast cancer patients. Some more validation of the proposed mechanisms should be warranted.
In the current study, the authors describe a panel of TNBC cell lines displaying very different abilities to metastasize and having highly different expression of miR-342. While 67NR and 66cl4 cells have high-level expression of miR-342-3p, these cell lines hardly metastasize when implanted in tumors. In Supplementary Figure 2A, the authors present relative miR-342-3p levels in the TNBC cell line panel they used and in the 4T1.2 cell lines having been transfected with miR-342. The data is normalized to the expression of miR-342 in 67NR cells. No expression of the miRNA is visible in the 4T1-derivatives, while the miR-342-transfected 4T1.2 cells show very strong 'overexpression' of this miRNA, even compared to 67NR cells. Unfortunately, this graph does not show expression levels of miR-342 in the 231HM control and 231HM miR-342 cells. In supplementary Figure 3, doxycyclin-induced overexpression of miR-342 in 231HM cells seems to be by a factor of 20-30 and this does not seem to have strong effects on tumor growth, tumor cell migration or invasion for these cells. The extent of overexpression in the 4T1.2 cells seems to be much higher compared to the 231HM cells. The same group recently published two highly interesting studies (PMID:40984977; 40251190) where expression levels and association of miRNAs with argonaute proteins were discussed in the context of true and likely artificial miRNAs. There, the authors very rightfully lay out that even untrue miRNAs may establish phenotypes when overexpressed at high levels. In PMID:40251190 they conclude that "establishing endogenous functionality as a first principal, achieved through the early implementation of miRNA inhibition, would vastly improve the cancer field which is plagued by overstated claims of miRNA function."
Having reported artificial effects of non-miRNAs when present in high levels, non-physiological levels also of true miRNAs might induce similarly non-physiological phenotypes. The authors should thus bring down endogenous levels of this miRNA in some cell line showing expression of this miRNA, like 67NR. Such experiments should validate the findings by repressing miR-342 in some weakly or non-metastatic cell line, also by testing the metastatic potential in absence and presence of a CDK4/6-inhibitor. This experimental system should be superior to the artificial overexpression system the authors used thus far. The fact that some observations made in the 4T1.2 cell line system could not be reproduced in the 231HM cell line might imply that expression levels of the miRNA could indeed matter.
Other:
The authors present in Supplementary Tables 2 and 3 3,393 miR-342-3p peaks across 2,334 genes and 4,877 miR-342-5p peaks across 2,314 genes having been identified via Ago-HITS-CLIP as high confidence binding events as well as non-canonical peaks lacking traditional seed sequences. Those numbers suggest that almost one quarter (up to 4648) out of

~20,000 human protein-coding genes are regulated by miR-342. Might these numbers be related to the very high levels of miR-342 in the experimental system? Integrating data from RNA-sequencing, these numbers are boiled down to 130 transcripts showing greater than 2-fold up-or downregulation for miR-342-3p and over 600 transcripts for miR-342-5p. Is endogenous expression of miR-342 strong enough to regulate the expression of all these genes? What were expression levels of miR-342 in this experiment? Are those levels similar to physiological levels in other systems? Later, the authors find much fewer transcripts that are regulated by miR-342. Am I correct to assume that the delta are indirect 'targets'? The authors observed that miR-342-5p might act primarily through 'non-canonical' mechanisms, including binding within coding regions. Are such 'non-canonical' mechanisms common or might these observations support the idea of potentially non-physiological binding of miR-342?
In the first paragraph of chapter 'Pharmacological inhibition of E2F...' the authors cite their reference 14 when they state that CDK4/6 inhibition would now be standard of care in the treatment of ER+ breast cancer. Similar as they do later in the manuscript, the authors should indicate also here that this is true mostly for the high-risk and advanced/metastatic setting. Early ER+ breast cancer is still treated mostly with endocrine therapies (+/- radiotherapy), as it is not clear if patients with early disease would benefit and because CDK4/6 inhibitors may induce severe adverse side effects that may preclude their application in a mostly curative setting.
In PMID:40251190, the authors questioned the utility of 3'UTR reporter assays for validation of miRNA-targets as even non-miRs would show regulation of some 'targets'. A very similar assay was used also in the current study. The authors should at least consider revisiting their conclusions as very high and non-physiological levels even of some bona fide miRNA might induce effects that are not necessarily physiological either (e.g., regarding competition of physiological and, potentially, non-physiological targets for miRNA-molecules).

Referee #2 (Comments on Novelty/Model System for Author):

It is confusing to me at this stage why the authors go from bioluminiscence to qPCR human vs mouse to score metastasis outgrowth depending on the panel while using the same model. See below the comments to authors

Referee #2 (Remarks for Author):

In this manuscript Arnet et al describe the role of miR-342-EDF network regulating triple negative breast cancer metastasis. Comparing different parental, poorly and highly metastatic mouse 4T1 BCa cell populations combined with clinical data, the authors uncover miR342 as a potential suppressor of TNBC metastasis. They extend this finding to a human BCa cell line. Subsequently, they explore several omic approaches to associate miR-342 expression with reduced E2F signaling. Further the authors suggest that in these circumstance, CDK4/6inhibitors may prevent cell growth. Overall, this is a manuscript, building on an interesting observation. However, some scarce in depth causal mechanistic analysis and some technical concerns limit the enthusiasm.

Conceptually, the observation that cells with different miR-342 levels grow differently in the primary site suggest that the miR effect goes beyond metastasis (Fig 1I and J). Subsequently the size match tumors suggest also a true metastasis component, yet uncoupling both mechanisms is of relevance. Previous work has suggested that miR-342 repress TNBC cell growth through MCT1, which is not accounted herein and may be relevant to separate both effects.

Further, The effect on tumor growth is not captured in vitro, is it stroma dependent? Is it factor stimuli dependent?

In figure 1 L and M and Fig 2P, the use of log transformed values may be helpful to interpret the bioluminescence data, as using millions and broken axis does not seem adequate. In line, why the metastases analyses in figure 2 using the human 231 xenograft that is labeled with luciferase (Fig 2C) are not quantified by bioluminescence as in figure 1? This is puzzling. In the latter a ratio of human versus mouse mRNA (qPCR) is used but this seems insufficient in this context particularly when the differences are marginal. Indeed, in supplementary figure 2D, the metastasis scoring using 4T1 BCa cells size-match primary tumors does not use bioluminescence to score metastasis (Supp Fig 2D). Again, the difference being marginal. Quantification using IHC or bioluminescence should be provided.

It is unclear why the more aggressive the 4T1 metastasis clone is, the higher the miR expression? (figure 1D). Please address.

231H cells are selected as those that express less miR342 (Figure 2A). In Figure 5, these cells, upon miR-342 are claimed to gain CDK4/6i response. How do the latter compared in terms of expression to the panel of BCa cells used in 2A? In those BCa cells expressing high levels of miR 342, what happens to CDK4/6i response upon the miR downregulation? Further, what happens when 231H miR342-expressing are treated with CDk4/6i? is there an additive effect? A synergistic effect? or both are mutually exclusive?

In page 7 figure 3N and 3O-P are mislabeled and should read figure 2N and 3O-P.

Through figure 3 and 4, the authors do a remarkable effort to comprehensively identify how miR342 drives the observed effect. However, how the data is integrated is confusing. The analyses at the reporter, mRNA and protein render different candidates,

some confronted to clinical data yet there is not a clear line that can be followed by the reader and drives to the logical conclusion that E2F network is central.
Of note, E2F is tested in 3H among other, but not in 3I, whereas the other are. Please address the inconsistencies and the flow.

What is the p53 and Rb mutational and expression status of 231H cells? The doses used for the in vitro analyses are very high on the microM range and not nano molar. MCF7 BCa cells (CDK4/6i responders) show a very strong inhibition by CDK4/6i in vitro at 10-50 nM. The use of higher doses in the herein described model seems inconsequential and out of range.

In figure 5, several approaches, it is unclear how much of the CDK4/6i effect is miR342 dependent. What is the mechanistic and causal relationship? the link with E2F reads an association rather than causal

Referee #1 (Comments on Novelty/Model System for Author):

The technical quality of the presented findings is high, however, there are some potential issues with the experimental setup. The authors should downregulate the miRNA in some cell line system (e.g., 67NR) to verify that the miRNA and not its non-physiological levels induce the observed phenotypes (see my comments to the authors).
The medical impact might be high, if the findings can be confirmed in a downregulation setting. The 67NR cell line could be a more adequate model system.

Referee #1 (Remarks for Author):

Arnet and co-workers analyzed the role of miR-343 in the context of metastasis in triple negative breast cancer. In a panel of TNBC tumor cell lines differing also in their potential to metastasize, they had identified miR-342 (among others) as strongly repressed in the metastasizing cell lines. To identify targets and phenotypes of this miRNA, they overexpressed miR-342 in the highly metastatic 4T1.2 cell line, sequenced the mRNA, performed some phenotypic assays and established in vivo metastasis models. Correlation of miR-342-expression with survival was established using sequencing (TCGA, METABRIC) and microarray datasets. Collectively, this led to the identification of a transcriptional network centering on E2F signaling and cell cycle regulation at G1/S transition. In vivo data in their syngeneic model suggested that overexpression of miR-342 could reduce formation of metastases, while no dramatic effects were observed on tumor growth. Consequently, they blocked CDK4/6 and observed that inhibition of these kinases abrogated metastasis potential of the cells similar as overexpression of miR-342 did. Furthermore, they observe in publicly available single cell RNA-seq data that E2F signaling and expression of the host gene of miR-342 is inversely correlated with metastasis formation. The authors conclude that they have identified a novel axis involving miR-342 and E2F as driver of metastasis formation in TNBC, potentially opening new perspectives for treatment of a subgroup of TNBCs that are wildtype for RB1, and to potentially prevent formation of metastases. This study touches a highly relevant clinical problem and brings up an interesting mechanism that might be targeted for the benefit of a subset of breast cancer patients. Some more validation of the proposed mechanisms should be warranted.
In the current study, the authors describe a panel of TNBC cell lines displaying very different abilities to metastasize and having highly different expression of miR-342. While 67NR and 66cl4 cells have high-level expression of miR-342-3p, these cell lines hardly metastasize when implanted in tumors. In Supplementary Figure 2A, the authors present relative miR-342-3p levels in the TNBC cell line panel they used and in the 4T1.2 cell lines having been transfected with miR-342. The data is normalized to the expression of miR-342 in 67NR cells. No expression of the miRNA is visible in the 4T1-derivatives, while the miR-342-transfected 4T1.2 cells show very strong 'overexpression' of this miRNA, even compared to 67NR cells. Unfortunately, this graph does not show expression levels of miR-342 in the 231HM control and 231HM miR-342 cells. In supplementary Figure 3, doxycyclin-induced overexpression of miR-342 in 231HM cells seems to be by a factor of 20-30 and this does not seem to have strong effects on tumor growth, tumor cell migration or invasion for these cells. The extent of overexpression in the 4T1.2 cells seems to be much higher compared to the 231HM cells. The same group recently published two highly interesting studies (PMID:40984977; 40251190) where expression levels and association of miRNAs with argonaute proteins were discussed in the context of true and likely artificial miRNAs. There, the authors very rightfully lay out that even untrue miRNAs may establish phenotypes when overexpressed at high levels. In PMID:40251190 they conclude that "establishing endogenous

functionality as a first principal, achieved through the early implementation of miRNA inhibition, would vastly improve the cancer field which is plagued by overstated claims of miRNA function."
Having reported artificial effects of non-miRNAs when present in high levels, non-physiological levels also of true miRNAs might induce similarly non-physiological phenotypes. The authors should thus bring down endogenous levels of this miRNA in some cell line showing expression of this miRNA, like 67NR. Such experiments should validate the findings by repressing miR-342 in some weakly or non-metastatic cell line, also by testing the metastatic potential in absence and presence of a CDK4/6-inhibitor. This experimental system should be superior to the artificial overexpression system the authors used thus far. The fact that some observations made in the 4T1.2 cell line system could not be reproduced in the 231HM cell line might imply that expression levels of the miRNA could indeed matter.
Other:
The authors present in Supplementary Tables 2 and 3 3,393 miR-342-3p peaks across 2,334 genes and 4,877 miR-342-5p peaks across 2,314 genes having been identified via Ago-HITS-CLIP as high confidence binding events as well as non-canonical peaks lacking traditional seed sequences. Those numbers suggest that almost one quarter (up to 4648) out of ~20,000 human protein-coding genes are regulated by miR-342. Might these numbers be related to the very high levels of miR-342 in the experimental system? Integrating data from RNA-sequencing, these numbers are boiled down to 130 transcripts showing greater than 2-fold up-or downregulation for miR-342-3p and over 600 transcripts for miR-342-5p. Is endogenous expression of miR-342 strong enough to regulate the expression of all these genes? What were expression levels of miR-342 in this experiment? Are those levels similar to physiological levels in other systems? Later, the authors find much fewer transcripts that are regulated by miR-342. Am I correct to assume that the delta are indirect 'targets'? The authors observed that miR-342-5p might act primarily through 'non-canonical' mechanisms, including binding within coding regions. Are such 'non-canonical' mechanisms common or might these observations support the idea of potentially non-physiological binding of miR-342?
In the first paragraph of chapter 'Pharmacological inhibition of E2F...' the authors cite their reference 14 when they state that CDK4/6 inhibition would now be standard of care in the treatment of ER+ breast cancer. Similar as they do later in the manuscript, the authors should indicate also here that this is true mostly for the high-risk and advanced/metastatic setting. Early ER+ breast cancer is still treated mostly with endocrine therapies (+/- radiotherapy), as it is not clear if patients with early disease would benefit and because CDK4/6 inhibitors may induce severe adverse side effects that may preclude their application in a mostly curative setting.
In PMID:40251190, the authors questioned the utility of 3'UTR reporter assays for validation of miRNA-targets as even non-miRs would show regulation of some 'targets'. A very similar assay was used also in the current study. The authors should at least consider revisiting their conclusions as very high and non-physiological levels even of some bona fide miRNA might induce effects that are not necessarily physiological either (e.g., regarding competition of physiological and, potentially, non-physiological targets for miRNA-molecules).

Referee #2 (Comments on Novelty/Model System for Author):

It is confusing to me at this stage why the authors go from bioluminiscence to qPCR human vs

mouse to score metastasis outgrowth depending on the panel while using the same model. See below the comments to authors

Referee #2 (Remarks for Author):

In this manuscript Arnet et al describe the role of miR-342-EDF network regulating triple negative breast cancer metastasis. Comparing different parental, poorly and highly metastatic mouse 4T1 BCa cell populations combined with clinical data, the authors uncover miR342 as a potential suppressor of TNBC metastasis. They extend this finding to a human BCa cell line. Subsequently, they explore several omic approaches to associate miR-342 expression with reduced E2F signaling. Further the authors suggest that in these circumstance, CDK4/6inhibitors may prevent cell growth. Overall, this is a manuscript, building on an interesting observation. However, some scarce in depth causal mechanistic analysis and some technical concerns limit the enthusiasm.

Conceptually, the observation that cells with different miR-342 levels grow differently in the primary site suggest that the miR effect goes beyond metastasis (Fig 1I and J). Subsequently the size match tumors suggest also a true metastasis component, yet uncoupling both mechanisms is of relevance. Previous work has suggested that miR-342 repress TNBC cell growth through MCT1, which is not accounted herein and may be relevant to separate both effects.

Further, The effect on tumor growth is not captured in vitro, is it stroma dependent? Is it factor stimuli dependent?

In figure 1 L and M and Fig 2P, the use of log transformed values may be helpful to interpret the bioluminescence data, as using millions and broken axis does not seem adequate. In line, why the metastases analyses in figure 2 using the human 231 xenograft that is labeled with luciferase (Fig 2C) are not quantified by bioluminescence as in figure 1? This is puzzling. In the latter a ratio of human versus mouse mRNA (qPCR) is used but this seems insufficient in this context particularly when the differences are marginal. Indeed, in supplementary figure 2D, the metastasis scoring using 4T1 BCa cells size-match primary tumors does not use bioluminescence to score metastasis (Supp Fig 2D). Again, the difference being marginal. Quantification using IHC or bioluminescence should be provided.

It is unclear why the more aggressive the 4T1 metastasis clone is, the higher the miR expression? (figure 1D). Please address.

231H cells are selected as those that express less miR342 (Figure 2A). In Figure 5, these cells, upon miR-342 are claimed to gain CDK4/6i response. How do the latter compared in terms of expression to the panel of BCa cells used in 2A? In those BCa cells expressing high levels of miR 342, what happens to CDK4/6i response upon the miR downregulation? Further, what happens when 231H miR342-expressing are treated with CDk4/6i? is there an additive effect? A synergistic effect? or both are mutually exclusive?

In page 7 figure 3N and 3O-P are mislabeled and should read figure 2N and 3O-P.

Through figure 3 and 4, the authors do a remarkable effort to comprehensively identify how miR342 drives the observed effect. However, how the data is integrated is confusing. The analyses at the reporter, mRNA and protein render different candidates, some confronted to

clinical data yet there is not a clear line that can be followed by the reader and drives to the logical conclusion that E2F network is central.
Of note, E2F is tested in 3H among other, but not in 3I, whereas the other are. Please address the inconsistencies and the flow.

What is the p53 and Rb mutational and expression status of 231H cells? The doses used for the in vitro analyses are very high on the microM range and not nano molar. MCF7 BCa cells (CDK4/6i responders) show a very strong inhibition by CDK4/6i in vitro at 10-50 nM. The use of higher doses in the herein described model seems inconsequential and out of range.

In figure 5, several approaches, it is unclear how much of the CDK4/6i effect is miR342 dependent. What is the mechanistic and causal relationship? the link with E2F reads an association rather than causal.

Referee #3 (Remarks for Author):

The study shows a strong association between miR-342 expression, reduced E2F signaling, and diminished metastatic outgrowth, but does not definitively establish E2F repression as the primary driver of the anti-metastatic phenotype.

The conclusions regarding CDK4/6 inhibition and E2F signaling do not fully account for RB pathway heterogeneity across TNBC models, leaving uncertainty about pathway dependence of the observed effects.

Reduced metastatic burden is interpreted as impaired metastatic outgrowth, yet the data do not clearly distinguish effects on proliferation, survival, or cell-cycle dynamics in metastatic sites.

MiR-342 regulates multiple pathways beyond E2F signaling, and the relative contribution of these additional targets to metastasis suppression remains unclear.

Although both miRNA strands affect E2F signaling, their individual and combined roles in regulating metastatic progression are not clearly resolved.

The anti-metastatic effects of palbociclib are attributed to E2F inhibition, but broader CDK4/6 inhibitor effects complicate interpretation of pathway-specific mechanisms.

The proposed TNBC subgroup is central to the study's translational claims, yet its definition and robustness across datasets are not fully established.

Clarify biological replicate numbers for all in vivo experiments in figure legends.

Specify whether randomization and blinding were used for mouse studies.

Indicate whether sex-specific effects were considered (all female mice are used).

For Ago-HITS-CLIP, clarify how non-canonical binding events were filtered or weighted.

Provide clearer rationale for EISA cutoffs and their robustness to threshold changes.

Ensure consistent terminology between "metastatic outgrowth," "colonization," and "burden."

Reviewer #1

Downregulation of miR-342 and examination of endogenous effects

We thank for the reviewer for their comments and as they correctly point out, based our recently published work, we are very aware that there are issues of misinterpreting biological effects of miRNAs with overexpression experiments. From the outset, we sought to test whether we could manipulate endogenous miR-342 activity and measure changes in gene expression and biological functions. To this end, we repressed endogenous miR-342 expression using miR-342 Anti-miRs across a range of mouse and human cells. Surprisingly, using the miR-342-3p Anti-miR we observed a broad inhibitory effect on cell health including in 67NR, 168FARN, 66cl4, and MCF-7 cells while these cells tolerated the miR-342-5p Anti-miR (see Reviewer Figure 1). In contrast, MDA-MB-231 cells which express low levels of miR-342 grew well in the presence of the miR-342-3p Anti-miR. Thus, the inhibitory effect of miR-342-3p Anti-miR appears to be specific and dependent on the amount of endogenous miR-342. We also trialled a different approach and deleted miR-342 from the intron of the EVL host gene using CRISPR in MDA-MB-231s cells. As expected, single clones of these cells survived, but displayed a large increase in EVL expression, suggesting that CRISPR deletion of miR-342 (even within the middle of a large intron) has unintended effects on host gene expression.

At this stage, we do not understand the mechanisms contributing to the dependence of breast cancer cell lines on miR-342-3p for their growth. However, we hypothesise that cells with lower levels of miR-342 have adjusted to loss of miR-342, at least in part, by increasing E2F pathway expression. Unfortunately, due to the strong impact of inhibiting miR-342-3p on cell health we were unable to gain data about the impact of miR-342 inhibition on gene expression and biological functions. As we are highly aware of the importance of assessing miRNA function at physiological levels, we instead utilised a range of constitutive and inducible systems where miR-342 was expressed within physiological range as detailed below.

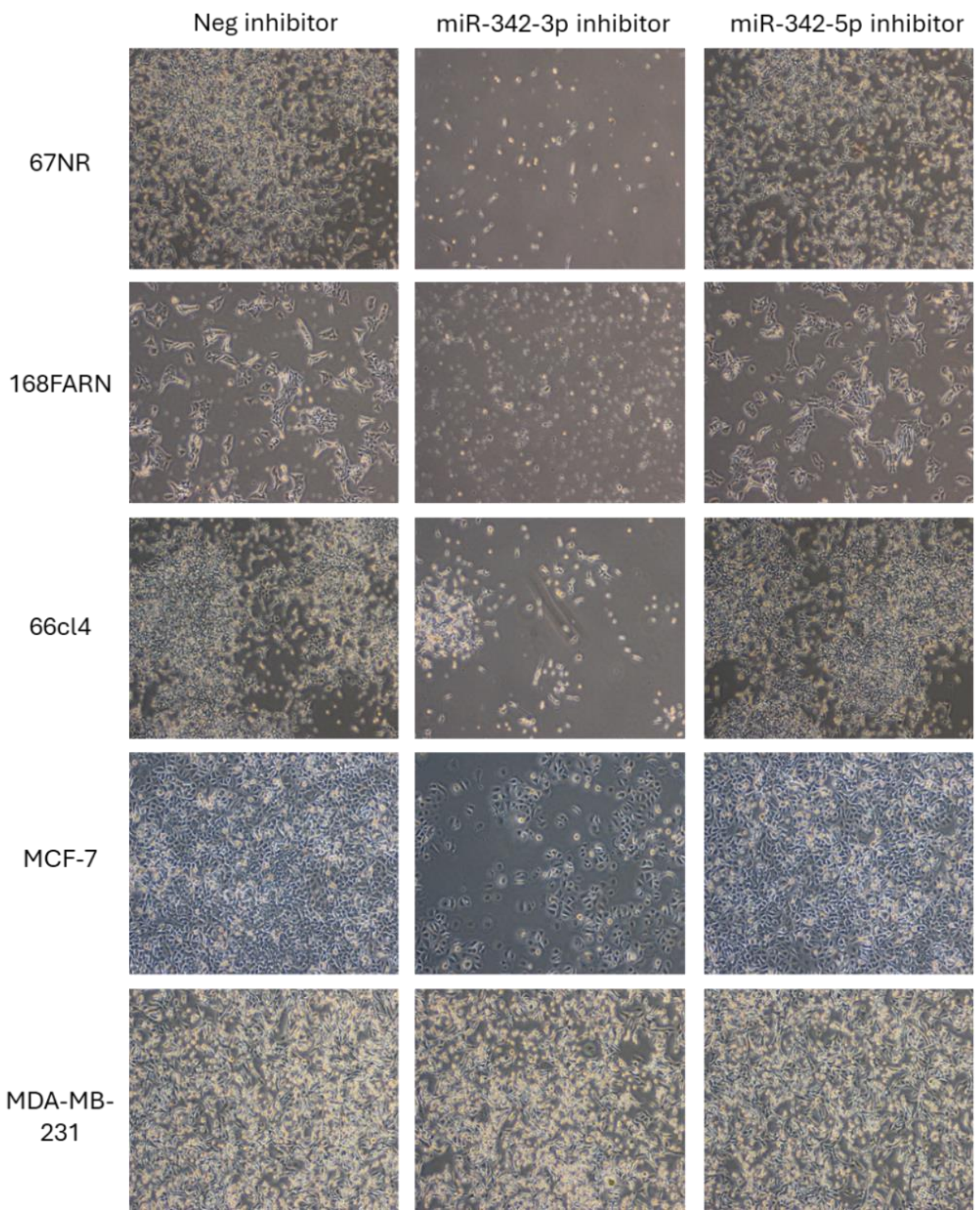

Reviewer Figure 1: Phase images of the indicated breast cancer cell lines following 72hr transfection with control, miR-342-3p, or miR-342-5p Anti-miRs (100nM, miRCURY LNA miRNA Inhibitors, Qiagen).

Questions about degree of miR-342 overexpression. Concerns about level being non-physiological.

The degree of miR-342 overexpression within the 4T1.2 and 231HM cells was measured at different times and therefore could not be accurately combined. However, general trends can be drawn in terms of examining cycle threshold. The cycle threshold (CT) of miR-342 overexpression in 4T1.2 cells reached a CT of 20 (Reviewer Figure 2). For 231HM cells the CTs of constitutive and inducible overexpression cells reached up to a value of 18 (as shown in Expanded View Figure 2G). The breast cancer cell line with the highest expression of miR-342 that we tested were MCF-7 cells with a CT=22 (see Review Figure 1 below). Within the CCLE data (Figure 2B), there are 7 cell breast cancer cell lines that have up to 8-fold (3 log) higher expression than MCF-7 (Reviewer Figure 2). Thus, the level at which we have overexpressed miR-342 is within physiological range. We have included additional commentary within the Results to make it clear

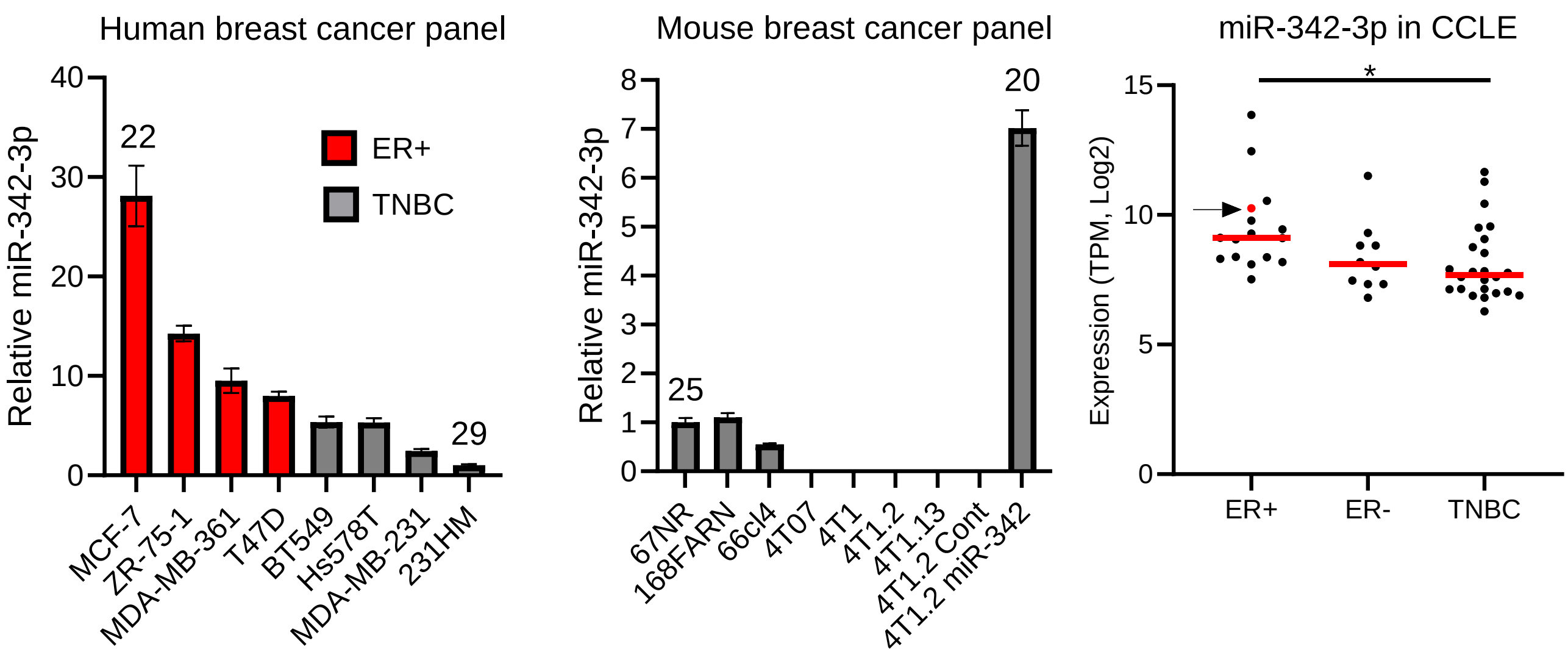

that we have expressed miR-342 within a physiological range

Reviewer Figure 2: Annotation of Figure 2A and Expanded View Figure 2A with CTs for cell lines with the highest and lowest miR-342 expression. For the CCLE data, MCF-7 cells are highlighted in red and with an arrow. Note that the middle panel had an incorrect value for 168FARN caused by a transposition error (originally 3.1 instead of 1.1). This has been corrected in the revised version.

Questions about Ago-HITS-CLIP data - degree of miR-342 overexpression, number of peaks, reporter assays, and non-canonical mechanisms

Using Ago-HITS-CLIP, we aimed to identify the direct targets of both miR-342-3p and miR-342-5p and therefore transfected these synthetic miRNAs independently into cells. This procedure is commonly used to identify direct miRNA targets (eg. PMID: 21389147, 25069772, 33300035, 33406413) although the resulting high levels of miRNA expression (typically 100x or more above background) can produce non-physiological interactions and a large number of identified peaks (as we observed in Supplementary Tables 2 and 3). Therefore, it is important to note that this is just <u>one step</u> within a series of experiments that leads to the identification of progressively more convincing miRNA target genes. Following Ago-HITS-CLIP, we filtered these candidates for downregulation in gene expression, and importantly post-transcriptional downregulation using the EISA technique. The post-transcriptional impact of a miRNA is calculated as the delta of exon read

changes (post-transcriptional and transcriptional) minus intron read changes (transcriptional only). Supporting the strength of this pipeline to identify genuine miRNA targets, we found that mRNAs that were post-transcriptionally regulated by miR-342 were enriched for HITS-CLIP binding sites with the greatest degree of downregulation correlating with 3’UTR binding, higher affinity seed matches and multiple binding sites (Expanded View Figures 3G and H) – factors well established to be hallmarks of canonical miRNA binding. Importantly, we also observed downregulation of these targets upon more physiological miR-342 overexpression (Expanded View Figure 4C).

As noted by the reviewer and described in our published work, reporter assays can be subject to misinterpretation where even non-miRNAs can repress an artificial target site when expressed at non-physiological levels. We include reporter assays only to assist the validation of targeting in Figure 3I to demonstrate that near full length 3’UTRs of identified target genes (through HITS-CLIP and EISA) are subject to miR-342 regulation.

For miR-342-5p, we observed greater changes in gene expression with potentially some of these resulting from non-physiological overexpression given the basal levels of miR-342-5p are on average ~16-fold lower than miR-342-3p (Reviewer Figure 3). However, the strongest direct targets more often had binding sites within the 3’UTR indicative of a genuine target and a canonical mechanism of miRNA action (Expanded View Figure 3F). The more modestly regulated targets tended to display coding region binding which suggests miRNA activity through non-canonical mechanisms. MiRNA binding to coding regions is frequently observed in Ago-CLIP sequencing datasets although examples of functional miRNA activity through these sites are much less common (eg. PMID: 23335364, 32821933, 33215742). With this in mind, we have moderated our description of this in the Results page 8 “For miR-342-5p, 19 of 45 mRNAs with identified binding sites were downregulated, though only 8 of these had sites in their 3′UTR suggesting that miR-342-5p may ~~act primarily~~ potentially act through non-canonical mechanisms, including binding within coding regions”.

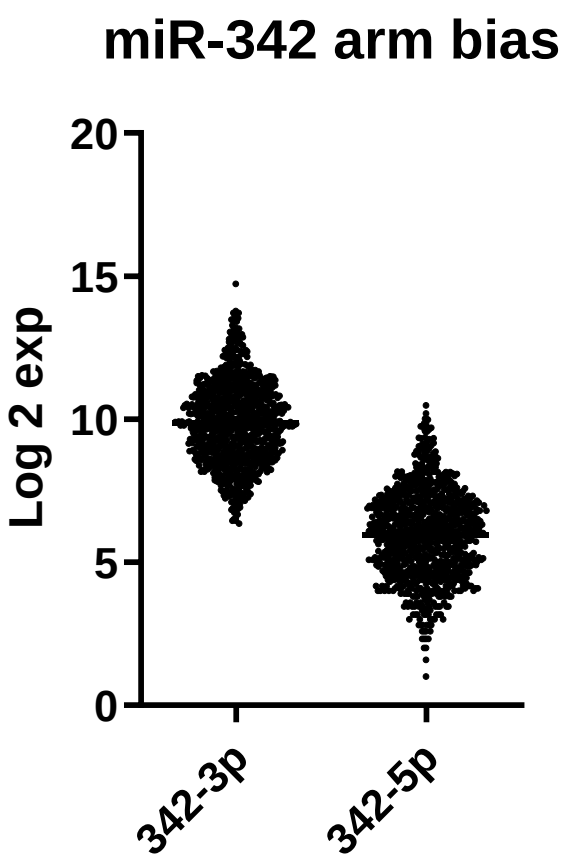

Reviewer Figure 3: Relative miR-342-3p and -5p expression in TCGA breast cancer data

A note on miRNA function: As pointed out by the reviewer, a widely recognised problem in the miRNA field is the assignment of a particularly important function of a miRNA based squarely on overexpression studies to one particular target. While of course sometimes that is accurate, it is often overstated because it relies on that miRNA very strongly regulating that gene and as noted in seminal studies, most miRNAs are “sculptors” rather than “sledgehammers” to gene expression (as reviewed by Bartel in Cell, 2018). For this reason, we approached this investigation from the perspective that miRNA function emerges from examining network-level regulation. Put another way, a biological function can emerge from the sum of more modest regulations to multiple connected genes, as much as it could from the strong regulation of a single gene. Although the miR-342–E2F regulatory network we identify here is novel, the concept of pathway-level targeting is well established (as we have reviewed in PMID: 27795564).

Comments regarding use of CDK4/6 inhibitors in early vs advanced breast cancer

The reviewer makes an important point, and we have modified the Results section to describe that CDK4/6 inhibitors are primarily used in high-risk and/or metastatic ER+ settings.

Reviewer #2

Previous work has suggested that miR-342 repress TNBC cell growth through MCT1, which is not accounted herein and may be relevant to separate both effects. Further, The effect on tumor growth is not captured in vitro, is it stroma dependent? Is it factor stimuli dependent?

We examined our Ago-HITS-CLIP and RNA-sequencing data and there was no significant miR-342 binding to MCT1 (*SLC16A1*) or change in its levels with miR-342 expression in 231HM cells. We also performed qPCR of *Slc16a1* in the 4T1.2 model and observed a modest increase in its levels in miR-342 OE cells (Reviewer Figure 4). Therefore, within these models, there is not strong evidence to implicate MCT1 as a relevant miR-342 target gene. The growth of tumour cells on a plastic substratum does not reveal the complexity and inter-cellular communication that occurs within a tumour. It is thus evident, as the reviewer suggests, that paracrine signalling between stromal cell such as fibroblasts or immune cells, is influencing to a small extent, primary tumour growth in the syngeneic tumour model, but not in the xenograft model where immune cell influence is greatly diminished.

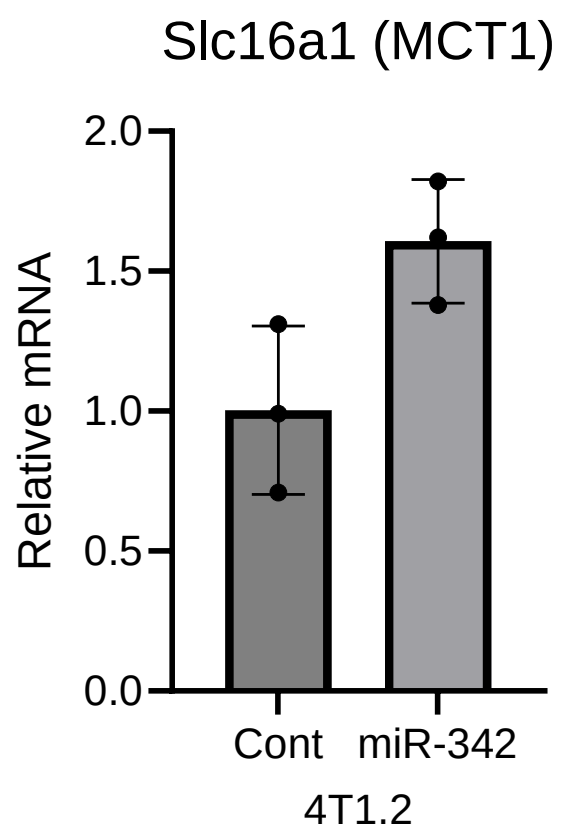

Reviewer Figure 4: Quantitative PCR of Slc16a1 levels in 4T1.2 control and miR-342 overexpressing cell lines

In figure 1 L and M and Fig 2P, the use of log transformed values may be helpful to interpret the bioluminescence data, as using millions and broken axis does not seem adequate. In line, why the metastases analyses in figure 2 using the human 231 xenograft that is labeled with luciferase (Fig 2C) are not quantified by bioluminescence as in figure 1? This is puzzling. In the latter a ratio of human versus mouse mRNA (qPCR) is used but this seems insufficient in this context particularly when the differences are marginal. Indeed, in supplementary figure 2D, the metastasis scoring using 4T1 BCa cells size-match primary tumors does not use bioluminescence to score metastasis (Supp Fig 2D). Again, the difference being marginal. Quantification using IHC or bioluminescence should be provided.

The metastasis experiments were performed in two different labs where different methods of quantitation were used to assess metastatic burden. For Figure 2, the experiment was performed in the Anderson lab where they routinely use genomic DNA qPCR of whole organs to quantitate metastasis (as described in their paper where 4T1 variants were first isolated, PMID: 15671244, and used widely), with luciferase only used to monitor tumour growth and the development of metastases *in vivo*. As whole organs are desiccated in this process, they are not available for IHC. For Figure 1, *ex vivo* bioluminescence of organs was used to measure metastasis as routine in the Gregory lab. We believe the convergence of findings demonstrating that miR-342 acts as a metastasis suppressor across different cell lines, mouse models and methods of metastatic burden assessment is a strength of our study. As suggested by the reviewer, we have modified the axes of the data in Figure 1 L and M to be in log scale with unbroken axes.

It is unclear why the more aggressive the 4T1 metastasis clone is, the higher the miR expression? (figure 1D). Please address.

While there appears to be a higher expression of miR-342-3p in the more metastatic 4T1 clones, this difference is minimal and non-significant by one-way ANOVA testing.

The analyses at the reporter, mRNA and protein render different candidates, some confronted to clinical data yet there is not a clear line that can be followed by the reader and drives to the logical conclusion that E2F network is central. Of note, E2F is tested in 3H among other, but not in 3I, whereas the other are. Please address the inconsistencies and the flow.

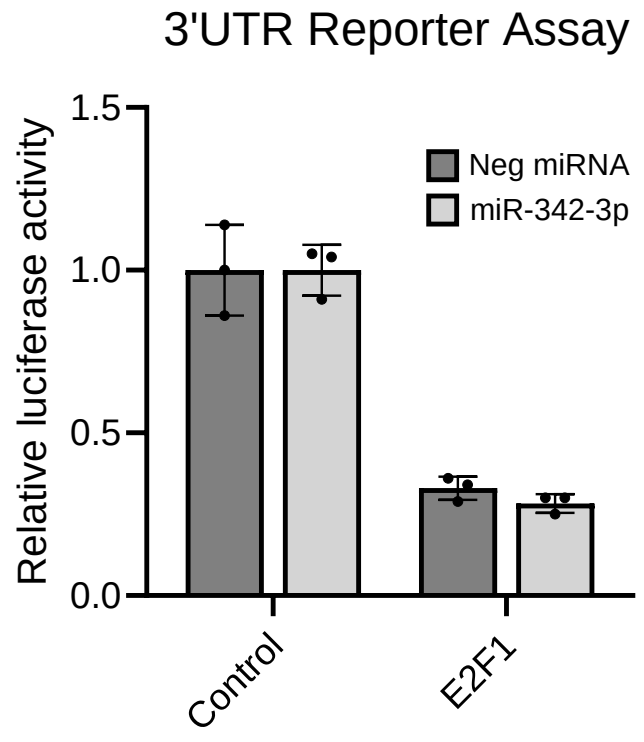

We cloned and tested the E2F1 3'UTR (858 bp, near full length) in reporter assays and found its basal activity was strongly suppressed relative to control, and consequently there was less scope to observe repression by co-transfected miR-342 (Reviewer Figure 5). This suggests other elements may contribute to the activity of the E2F1 3'UTR in this reporter context. However, our Ago-HITS-CLIP and EISA data, together with changes in protein and RNA clearly indicate E2F1 is a direct miR-342-3p target gene.

Reviewer Figure 5: Luciferase reporter assay of control (no 3'UTR) or E2F1 3'UTR transfected with miR-342-3p or control miRNA in 231HM cells.

<u>Questions about CDK4/6i inhibitor effect and dependence on miR-342</u>

231H cells are selected as those that express less miR342 (Figure 2A). In Figure 5, these cells, upon miR-342 are claimed to gain CDK4/6i response. How do the latter compared in terms of expression to the panel of BCa cells used in 2A? In those BCa cells expressing high levels of miR 342, what happens to CDK4/6i response upon the miR downregulation? Further, what happens when 231H miR342-expressing are treated with CDk4/6i? is there an additive effect? A synergistic effect? or both are mutually exclusive?

What is the p53 and Rb mutational and expression status of 231H cells? The doses used for the in vitro analyses are very high on the microM range and not nano molar. MCF7 BCa cells (CDK4/6i responders) show a very strong inhibition by CDK4/6i in vitro at 10-50 nM. The use of higher doses in the herein described model seems inconsequential and out of range.

In figure 5, several approaches, it is unclear how much of the CDK4/6i effect is miR342 dependent. What is the mechanistic and causal relationship? the link with E2F reads an association rather than causal

Both 231HM and parental MDA-MB-231 cells have elevated levels of E2F1 and downstream targets relative to cell lines with higher miR-342 expression in the breast cancer panel (Reviewer Figure 6). We confirmed that 231HM cells have both the TP53 R280K mutation and basal hyperphosphorylation of RB, as observed in parental MDA-MB-231 cells (Reviewer Figure 6 and 7). However, it is important to note that the response to CDK4/6i is dependent on many factors including the activity of other upstream kinases such as CDK2–Cyclin E, and not only the RB-E2F pathway. MCF-7 and other ER+ breast cancer lines typically have a higher dependence on the CDK4/6 pathway due the actions of ER signaling to drive CDK4/6 activity. In contrast, TNBC can display elevated cyclin E1, CDK2, and in some cases loss of RB1 expression that promotes CDK4/6i independence. As previously observed, the elevation of CDK2-Cyclin E and hyperphosphorylation of RB renders MDA-MB-231 cells (and indeed TNBCs in general) less sensitive to CDK4/6i than ER+ breast cancers (eg. PMID: 28606920). The effective 100nM dose we use is well within the range used for most CDK4/6i studies (eg. PMID: 28947417, 28606920) although, as the reviewer points out, lower doses (10-50nM) are often effective in cells that are highly dependent on CDK4/6 signaling.

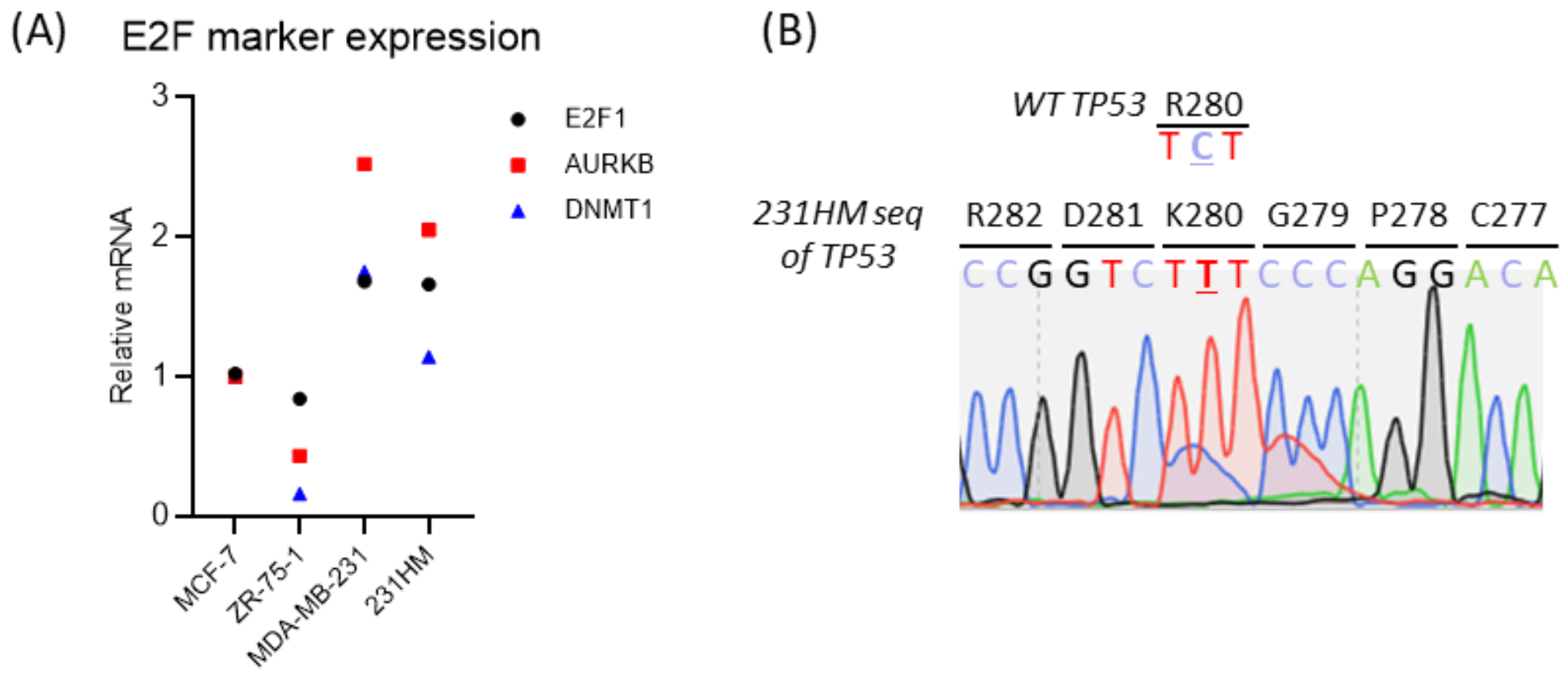

Reviewer Figure 6: (A) Quantitative PCR measuring the expression of E2F1 and downstream target genes in ER+/miR-342 high (MCF-7, and ZR-75-1) compared with TNBC/miR-342 low (MDA-MB-231 and 231HM) cell lines. (B) Genotyping of p53 in 231HM cell line showing it possesses the R280K mutation present in parental MDA-MB-231 cells.

The reviewer raises an important question about the relationship of miR-342 expression and the CDK4/6i effect. To determine whether the response of 231HM cells to CDK4/6i are influenced by miR-342, we performed a palbociclib dose-response colony formation assay in the absence or presence of miR-342 (induced with Dox in the 231HM-i342 cells as suggested). This included palbociclib levels ranging from 25-200nM. As we observed previously, neither palbociclib or Dox treatment affected colony number (Figures 5 and Expanded View Figure 5), however, both treatments reduced colony size consistent with their *in vivo* impacts on metastatic outgrowth (Reviewer Figure 7). Co-treatment of lower doses of palbociclib (25 and 50nM) with Dox led to a significant and additive decrease in colony size relative to the individual treatments alone, while this effect was not as evident at higher doses (100 and 200nM) due to their stronger impact on colony outgrowth. Examination of E2F1 also showed dose dependent and additive effects on its gene expression consistent with it directly impacting colony outgrowth. We also observed a dose dependent effect of palbociclib on RB phosphorylation consistent with it operating through this canonical pathway.

Collectively, our data demonstrate that CDK4/6i and miR-342 additively repress E2F signaling and colony outgrowth. While we are unable to directly test whether the CDK4/6i response is dependent on endogenous miR-342 levels (see response to reviewer 1), the strong inverse association of miR-342 with E2F signaling in patient TNBC samples and its correlation with metastasis (Figure 4 and 5L) indicates that tumours with low miR-342/high E2F signaling may benefit from treatment with CDK4/6i or miR-342, with a combination therapy potentially being most effective. We have added these data as Expanded View Figure 5F-I and have described these findings in the Results with commentary on its implications within the Discussion.

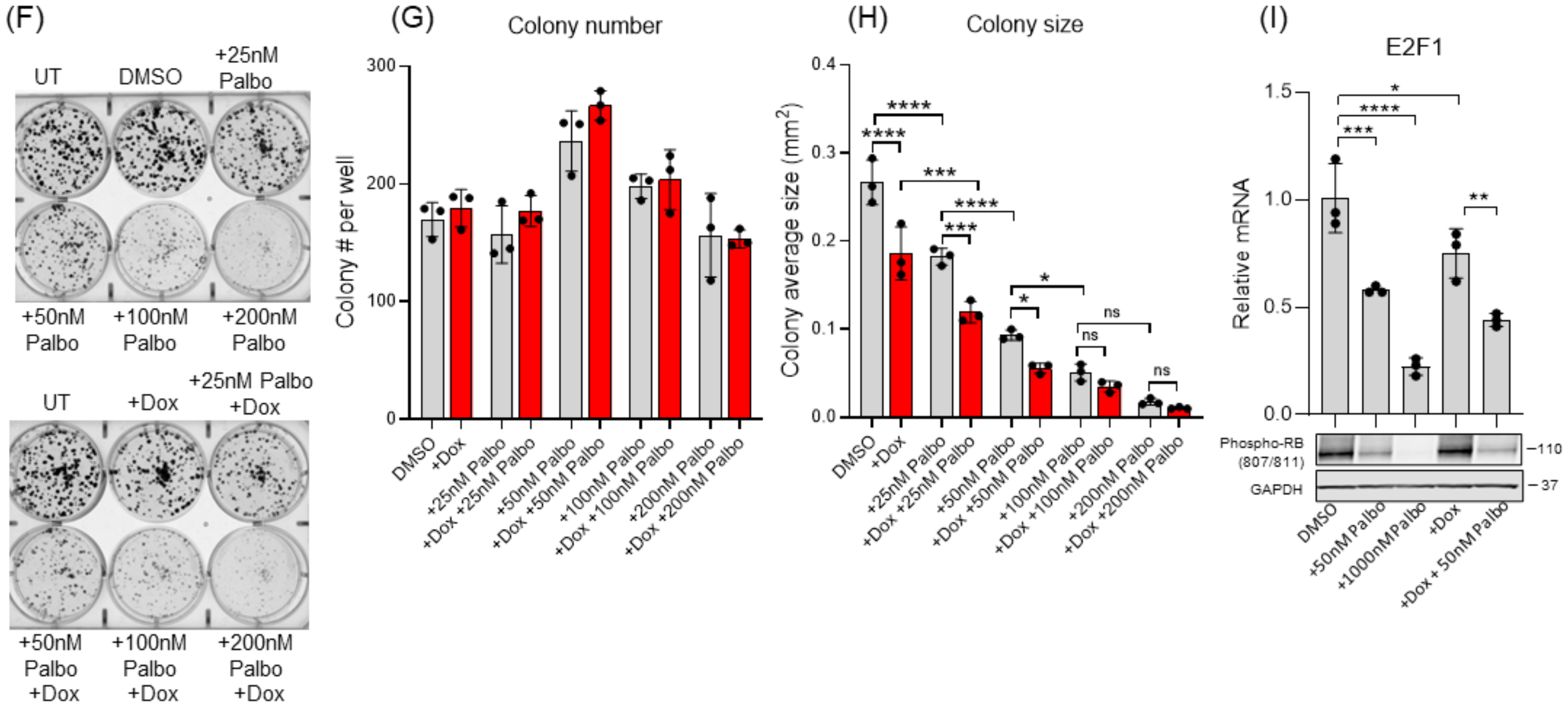

Reviewer Figure 7 (now Figure EV5 F-I): (F) Colony formation assays following individual or co-treatment of 231HM-i342 cells with Dox to induce miR-342 and different doses of palbociclib (harvest on d12). UT – untreated. (G-H) Quantitation of colony number and size with the indicated treatments (red bars are co-treatments). Data are plotted as mean ± SD (n=3). Significance was measured with one-way ANOVA with Bonferroni post-hoc tests, ns – not significant, *p<0.05 ***p<0.001 ****p<0.0001. (I) Quantitative PCR of E2F1 (upper panel) and Western blot of phosphorylated RB (lower panel) following treatment of 231HM-i342 cells with palbociclib and/or Dox for 24hr to measure immediate responses to treatment. Data are plotted as mean ± SD (n=3). Significance was measured with one-way ANOVA with Bonferroni post-hoc tests, *p<0.05 **p<0.01 ***p<0.001 ****p<0.0001.

In page 7 figure 3N and 3O-P are mislabeled and should read figure 2N and 3O-P.

Thank you for picking up this error which we have corrected.

Reviewer #3

The study shows a strong association between miR-342 expression, reduced E2F signaling, and diminished metastatic outgrowth, but does not definitively establish E2F repression as the primary driver of the anti-metastatic phenotype.

The conclusions regarding CDK4/6 inhibition and E2F signaling do not fully account for RB pathway heterogeneity across TNBC models, leaving uncertainty about pathway dependence of the observed effects.

Reduced metastatic burden is interpreted as impaired metastatic outgrowth, yet the data do not clearly distinguish effects on proliferation, survival, or cell-cycle dynamics in metastatic sites.

MiR-342 regulates multiple pathways beyond E2F signaling, and the relative contribution of these additional targets to metastasis suppression remains unclear.

Although both miRNA strands affect E2F signaling, their individual and combined roles in regulating metastatic progression are not clearly resolved.

The anti-metastatic effects of palbociclib are attributed to E2F inhibition, but broader CDK4/6 inhibitor effects complicate interpretation of pathway-specific mechanisms.

The proposed TNBC subgroup is central to the study's translational claims, yet its definition and robustness across datasets are not fully established.

The reviewer raises important points about the limitations of our study. Some of these, including the role of additional miR-342 targets and the function of the individual miR-342 strands, were briefly mentioned within the Discussion but we have now expanded on these, and on each of the other points raised by the reviewer, so that the reader is clear on current limitations and scope for future work. Importantly, while multiple lines of evidence support strong regulation of the E2F pathway by miR-342, we are not claiming that it is the sole pathway through which miR-342 exerts an anti-metastatic effect and have reiterated this further in the Discussion. We acknowledge that there will inevitably be heterogeneity in CDK4/6i response in TNBC due to multiple factors including RB activity, elevated levels of upstream pathways (eg. CDK2-Cyclin E), and MYC expression among others that all impinge on E2F signaling. However, our new findings showing that CDK4/6i and miR-342 cooperatively dampen E2F signaling and colony outgrowth (Reviewer Figure 7 and new Expanded View Figure 5F-I) indicate a convergence of these effects, suggesting TNBCs with low miR-342/high E2F signaling may benefit from co-treatment, although we acknowledge that this will require further exploration. We have modified our abstract to better reflect our current understanding of potential TNBC subgroups that may be amenable to CDK4/6i therapy.

Clarify biological replicate numbers for all in vivo experiments in figure legends.

We have clarified the biological replicate numbers in the figure legends.

Specify whether randomization and blinding were used for mouse studies.

We did not conduct specific randomization or blinding for mouse studies, although several investigators were involved across different laboratories to minimise potential biases. We have added this information to the Methods section.

Indicate whether sex-specific effects were considered (all female mice are used).

Sex specific effects weren't considered as breast cancer is a disease which primarily affects females and the majority of experiments were mammary fat pad (orthotopic) only relevant to female mice.

For Ago-HITS-CLIP, clarify how non-canonical binding events were filtered or weighted.

For the Ago-HITS-CLIP, peaks had to have a peak score of greater than or equal to 5 (ie. 5 reads or more above background as per Supplementary Tables 2 and 3) for it to be considered as a potential miR-342 target gene. For either canonical or non-canonical binding events to be classified as functional (ie.a miR-342 target gene), their transcripts had to be significantly repressed in expression (q<0.05) with EISA also demonstrating post-transcriptional repression. We have added this additional information to the Methods section.

Provide clearer rationale for EISA cutoffs and their robustness to threshold changes.

The EISA cutoff was any transcript with a negative "exon minus intron score", indicating post-transcriptional repression. This criterion, in addition to the others mentioned above, were used to classify miR-342-3p and -5p target genes. Within the miR-342 target gene lists, there is a strong correlation of EISA and the degree of gene repression (Reviewer Figure 8). As the degree of repression also correlate with more 3'UTR targeting (see Figure 3F and Expanded View Figure 3F) this suggests that some, but not all, of the non-canonical targets are of lower confidence and that the EISA cutoff chosen could be further refined to generate a higher confidence target list. For the network visualisation, transcriptional EISA thresholds were adopted to generate gene lists of

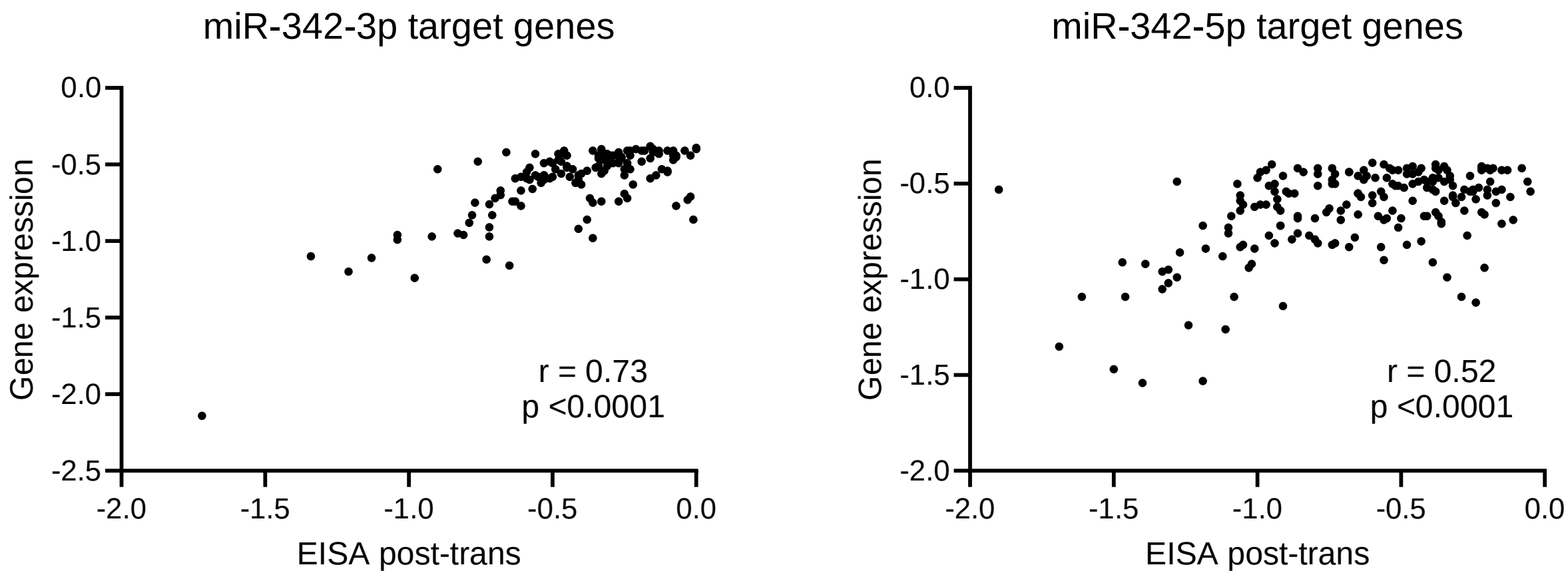

similar size to the miR-342-3p and -5p target genes as described in the Methods.

Reviewer Figure 8: Correlation of EISA post-transcriptional repression and gene expression in miR-342-3p and -5p target gene lists.

Ensure consistent terminology between "metastatic outgrowth," "colonization," and "burden."

We have corrected this terminology throughout the manuscript and use the term "colonisation" to only refer to initial metastatic seeding while "outgrowth" refers to growth of these initial deposits at the metastatic site ("burden" is now not used to avoid confusion).

27th Apr 2026

Decision on your manuscript EMM-2025-23012-V2

Dear Prof. Gregory,

Thank you for submitting your revised manuscript to EMBO Molecular Medicine. I apologize for the delay in our response, as one referee required additional time to complete their report. We have now received feedback from all three original referees.

As detailed below, while referee #3 is satisfied with the revisions, referees #1 and #2 raise two remaining major concerns:
• Lack of validation of the overexpression findings through knockdown or knockout of the miRNA in cell lines with high endogenous expression
• Use of, and comparison between, two different methodologies for metastasis quantification
In light of these issues, the referees remain unconvinced that the data fully support the conclusions.

Given the nature of these concerns, we regret that we are unable to consider your manuscript further.

I am very sorry to disappoint you in this occasion, and hope that the referees' comments are helpful in your continued work in this area.

With kind regards,

Lise Roth

Lise Roth
Senior Editor
EMBO Molecular Medicine

***** Reviewer's comments *****

Referee #1 (Comments on Novelty/Model System for Author):

A potential medical impact of the study remains unclear as the results from the experiments having performed during the revision process do not fully support the hypothesis about miRNA-342 in the context of growth and metastasis. Failure to validate overexpression by knocking down or out the miRNA in cell lines with high endogenous expression is an issue that should not be disregarded. The level of novelty can thus not be assessed given the presented data.

Referee #1 (Remarks for Author):

The authors repressed endogenous miR-342 expression using miR-342 Anti-miRs across a range of mouse and human cells, finding that this was toxic (?) in cell lines expressing miR-342, while this did not affect a cell line that lacked expression of the miRNA. Furthermore, this effect appeared to be strand-specific (only 3p effective). As the authors conclude, this observation was unexpected and, indeed, this does not support the role the miRNA is claimed have in metastasis. The authors should describe their findings in the manuscript and discuss them in the context of their claims.

Referee #2 (Comments on Novelty/Model System for Author):

Using to different methodologies and compare them, particularly when one of them reports very marginal differences is not standard. A reference from 2005 to justify not using bioluminescence (when the technology was barely available) seems poor. Quantification using IHC or bioluminescence is needed.

Referee #2 (Remarks for Author):

I have revised the manuscript again including all the provided changes. Overall, the authors have made a substantial effort to provide mechanistic angle regarding the role of regarding the p53 and Rb mutational and expression status of 231H cells. Some efforts have been made to the higher doses of CDK4/6i used as well as to clarify the different levels of miR342 expression and it's the causal relationship to CDK4/6i response. Clearly the review process has improved this. However, not a single effort has been made to streamline the message in the main text regarding the data integration which is still confusing to this reviewer.

My most serious concern is how metastasis was quantified. Using to different methodologies and compare them, particularly when one of them reports very marginal differences is not standard. A reference from 2005 to justify not using bioluminescence (when the technology was barely available) seems poor. Quantification using IHC or bioluminescence is needed.

Referee #3 (Comments on Novelty/Model System for Author):

The novelty is high as the miR-342-E2F network was not described in metastatic TNBC yet

Referee #3 (Remarks for Author):

The revised manuscript is well-improved and of great interest for the readers of this journal.

As a service to authors, EMBO provides authors with the possibility to transfer a manuscript that one journal cannot offer to publish to another EMBO publication. The full manuscript and if applicable, reviewers reports are automatically sent to the receiving journal to allow for fast handling and a prompt decision on your manuscript. For more details of this service, and to transfer your manuscript to another EMBO title please click on Link Not Available

Please do not share this URL as it will give anyone who clicks it access to your account.

Dear Lise,

Thank you for meeting with us last week and for the helpful discussion. I have attached our provisional rebuttal letters, separately for each referee, and also the miRNA inhibition data that we have proposed to Referee #1 that we include in our manuscript as they suggested in their comments to our rebuttal (new Figure EV3).

We look forward to hearing your response.

Best Regards

Philip and Robin

Dear Philippe, dear Robin,

I have now received feedback from both Referees #1 and #2, and I include excerpts from their reports below:

Referee #1:
"The authors have indeed taken seriously the concerns that I had. I feel that they have dealt with the issue in a reasonable way, also by now discussing the potential limitations of their findings."

Referee #2:
"I carefully reviewed the authors' comments and evaluated their arguments in detail. In particular, I examined PMID: 34168137 (Figure 5), where I could not identify any qPCR data, nor is it explicitly mentioned in the manuscript. In contrast, PMID: 37099632 (Figure 2) provides a direct comparison between luminescence/fluorescence measurements and gDNA qPCR analyses. Importantly, in that study, the magnitude of the measurements obtained with both approaches was comparable, which does not appear to be the case in the manuscript currently under discussion.

I do understand the authors' point that qPCR-based approaches have historically been used for these types of measurements. However, methodologies and technologies have evolved substantially, and newer approaches may provide improved robustness and consistency. If you believe the manuscript could still be of value to the journal, a more explicit discussion of these methodological aspects, together with the inclusion of additional references, would likely strengthen the study.

Regarding the data integration suggestions, I believe these additions would certainly be helpful."

Having considered the referees' responses and discussed your manuscript further within the editorial team, we would be willing to consider a substantially revised version of the manuscript, revised broadly along the lines outlined in your rebuttal letter and taking into account the referees' comments.

We would also like to emphasize that, with regard to the concerns raised by Referee #2, we would still encourage the inclusion of an additional independent replication, as this would further strengthen the overall robustness of the study.

Please note that any resubmission would be treated as an appeal and would therefore be sent out for external review again. At this stage, we cannot guarantee a favourable outcome.

Please do not hesitate to let me know if you would like to discuss the revisions further.

With kind regards,

Lise

Lise Roth, PhD
Senior Editor
EMBO Molecular Medicine

Response to Referees following proposed second amendments to the manuscript

We thank the Referees for their constructive feedback. In response to the remaining concerns of Referees #1 and #2, we have made substantial changes to the manuscript, including the addition of miRNA inhibition data and new analysis of metastasis data in accordance with the Referees suggestions. These new changes, along with those made in the first re-submission in response to comments from all 3 referees, have been submitted as track changes in the attached document. We trust you find our study is greatly improved and now suitable for publication in EMBO Molecular Medicine.

We have included the Referees response to our proposed amendments below followed by the actual amendments detailed in RED.

Referee #1 – Response of Referee to Editor following submission of proposed amendments:

"The authors have indeed taken seriously the concerns that I had. I feel that they have dealt with the issue in a reasonable way, also by now discussing the potential limitations of their findings."

Response to Referee #1 with proposed amendments:

Thank you for your constructive comments on our study. We agree that examining the effects of endogenous miR-342 is important and should not be disregarded. We initially chose not to include the miRNA inhibition data because we do not yet have a mechanistic explanation for the observed growth defect, and we felt that this finding would detract from the main emphasis and findings of the manuscript. However, the anti-miR experiments demonstrated that the growth-inhibitory effect was specific to the miR-342-3p strand and was observed only in cells expressing high levels of miR-342, consistent with a direct effect on the predominant mature product derived from the miR-342 precursor.

As described in our rebuttal, we also generated CRISPR-mediated knockout clones of miR-342 in metastatic MDA-MB-231 cells, which express low levels of miR-342, and obtained multiple viable homozygous knockout clones. We also applied the same strategy to non-metastatic MCF-7 cells, which express high levels of miR-342, and were unable to isolate any complete knockout clones (only heterozygous clones) despite several months of effort. Although preliminary, these findings are consistent with the miRNA inhibition data and support the notion that miR-342 is required for the growth of non-metastatic breast cancer cells.

Taken together, these miRNA knockdown studies suggest that miR-342 is important for the proliferation of non-metastatic cells, whereas metastatic cells appear to overcome this

growth inhibitory effect of miR-342 loss through other mechanisms, potentially including activation of E2F signaling. This interpretation is consistent with the broad concept that the acquisition of metastatic potential requires multiple genetic and epigenetic alterations. As suggested by the reviewer, we would greatly appreciate the opportunity to add the miRNA inhibition data and commentary to the paper (please find new Figure EV3 attached), acknowledging that this is an important limitation that prevents us assessing the direct impact of miR-342 repression on metastasis.

We would also like to emphasize that these findings do not diminish our conclusion that the miR-342–E2F axis regulates metastatic competency. Our results are highly specific and unlikely to be artefactual. We show restoration of miR-342 to physiological levels specifically suppressed metastatic outgrowth, without reproducible effects on primary tumour growth, cell migration, or invasion. This was demonstrated using mouse and human cell lines, with two different routes of cell administration, and with two different methods of miR-342 induction. In addition, colony formation assays showed that miR-342 reduced colony size, but not colony number, again demonstrating a specific effect of miR-342 in regulating outgrowth capacity (rather than initial colony establishment).

We further demonstrated that miR-342 directly modulates the E2F pathway at multiple levels - evidenced by direct HITS-CLIP binding of miR-342 and its post-transcriptional regulation of many E2F pathway members. These mechanistic findings are supported by strong correlations observed in patient tumour datasets and single-cell transcriptomic analyses, which collectively show that the miR-342–E2F axis is associated with TNBC metastatic progression and patient outcome, all of which is reinforced by our *in vivo* experiments.

As suggested by the reviewer, we would appreciate the opportunity to include these miRNA inhibition data in the revised manuscript and to discuss them in the context of our broader findings. We propose to present these results as new Expanded View EV3 (attached), describing these data on page 6 of the Results section (after we introduce the human cell lines in Figure 2A-B), and also within as a separate paragraph in the Discussion, noting it as an important limitation of our study.

Response to Referee #1 detailing new amendments to the manuscript:

1) We have written a new section in the Results on pages 7 and 8 describing the miR-342 inhibition data (see below), which is now included as Expanded View Figure 3. Details of these methods have also been included in the Methods section.

Expanded View Figure 3

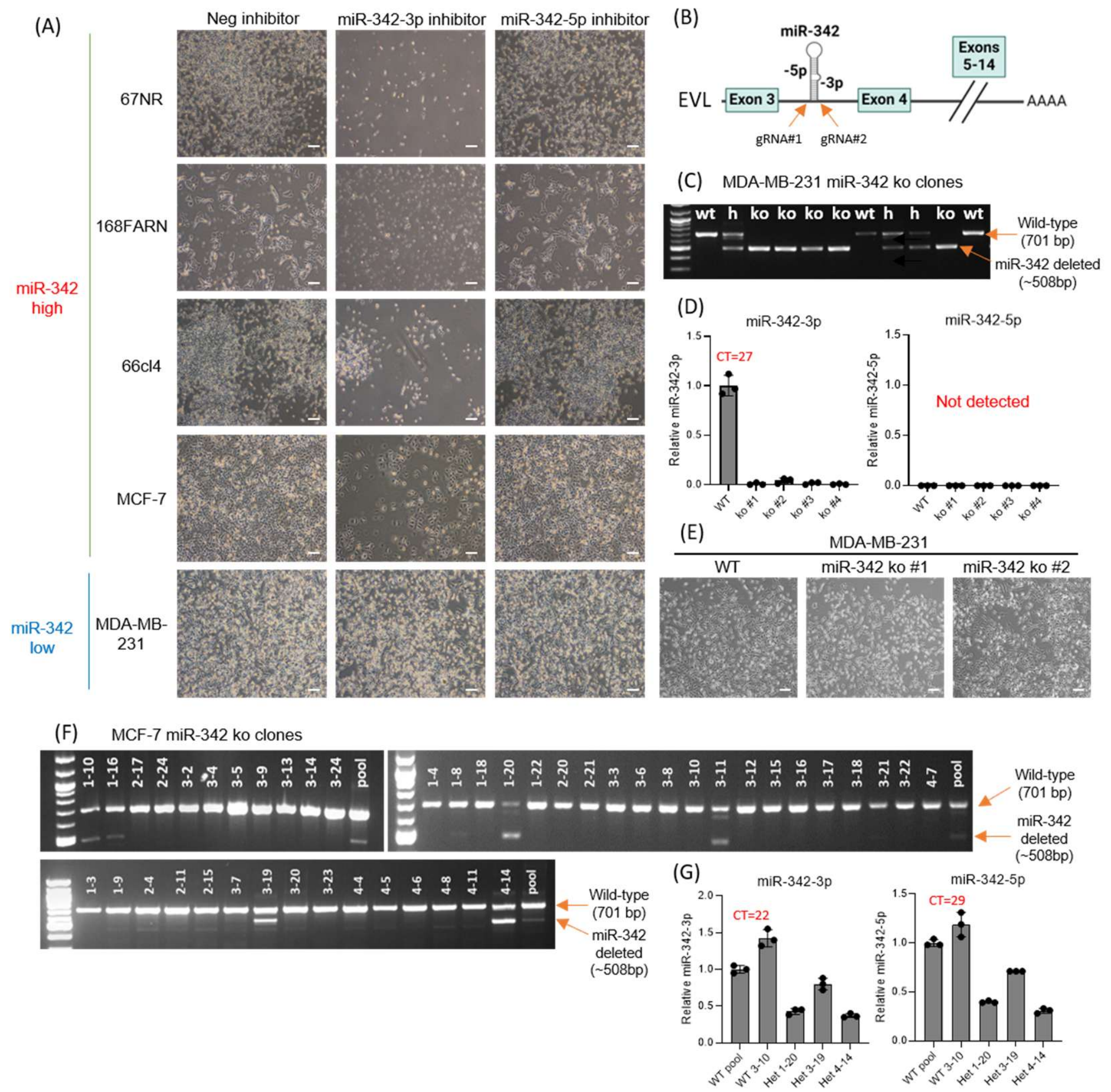

Figure EV3: miR-342 inhibition in mouse and human breast cancer cell lines.

(A) Phase contrast images of miR-342 high or miR-342 low expressing mouse and human breast cancer cell lines following 72hr transfection with control, miR-342-3p, or miR-342-5p Anti-miRs. Scale bars represent 50µm. (B) Schematic of miR-342 knockout (ko) approach using dual guide RNAs to cut out pre-miR-342 from the EVL host gene. (C) Gel showing PCR screen of genomic DNA isolated from MDA-MB-231 miR-342 ko clones (wt-wild-type, h-heterozygous). The size of the wild-type and full deletion of miR-342 based on the gRNA cut sites are shown. (D) Quantitation of miR-342-3p and -5p levels in WT and 4 different miR-342 ko clones. Cycle threshold (CT) of miR-342-3p in WT is shown. (E) Phase contrast images of WT and representative miR-342 ko clones. Scale bars represent 50µm. (F) Gels showing PCR screen of genomic DNA isolated from MDA-MB-231 miR-342 ko clones (clone numbers shown above; pool is cells collected after transfected with gRNA#1 and #2). The size of the wild-type and full deletion of miR-342 based on the gRNA cut sites are shown. (G) Quantitation of miR-342-3p and -5p levels in WT pool (parental cells), a WT single clone, and 3 different miR-342 heterozygous clones. The Cycle Threshold (CT) of miR-342-3p and 5p expression in WT pool is shown in red.

NEW RESULTS SECTION - MiR-342 is required for the growth of breast cancer cells with high endogenous miR-342 expression

To determine whether endogenous miR-342 suppresses metastatic potential in mouse and human breast cancer cell lines, we first examined the effects of transient miRNA inhibition in four cell lines that intrinsically express high levels of miR-342 and display low metastatic potential (Figures 1A, 1D, 2A–B, and EV2A). Unexpectedly, inhibition of miR-342-3p markedly reduced the growth of all four cell lines, whereas cells transfected with a miR-342-5p inhibitor behaved similarly to negative control transfected cells (Figure EV3A). In contrast, inhibition of miR-342-3p had no effect on the growth of MDA-MB-231 cells, which express intrinsically low levels of miR-342 (Figures 2A–B and EV3A). As miR-342-3p is the predominant mature product generated from the miR-342 precursor (Figures EV1C–D), these findings suggest that cells with high endogenous miR-342-3p expression may depend on this miRNA for proliferation.

To validate this observation using an independent approach, we employed a paired-guide RNA CRISPR strategy to delete the miR-342 precursor sequence in MDA-MB-231 cells (Figure EV3B). This approach generated many miR-342 knockout clones, which were confirmed by genomic PCR and quantitative PCR analysis of miR-342-3p and miR-342-5p expression (Figure EV3C–D). Consistent with the transient inhibition experiments, miR-342 knockout clones exhibited normal growth and were indistinguishable from wild-type MDA-MB-231 cells (Figure EV3E). We next applied the same strategy to MCF-7 cells, which express high endogenous levels of miR-342 (Figure 2A–B). However, despite extensive screening, no complete miR-342 knockout clones were recovered and only a small number of heterozygous clones were found (Figure EV3F–G). Together, these findings indicate that miR-342 is important for the growth of breast cancer cells with low metastatic potential that express high endogenous levels of this miRNA. We hypothesise that highly metastatic TNBC cells, which express low levels of miR-342, have acquired alternative mechanisms to bypass this requirement, potentially through the activation of miR-342 target genes and downstream pathways.

2) We have written a new section in the Discussion on page 16 (see below) discussing the implications of the miR-342 inhibition data and its limitations for our study.

NEW DISCUSSION SECTION “To determine whether endogenous miR-342 loss is sufficient to promote metastatic capacity, we inhibited each mature miR-342 species using transient anti-miRs or achieved sustained miR-342 loss through CRISPR-mediated deletion of the miR-342 precursor. Unexpectedly, loss of miR-342-3p markedly impaired the growth of several breast cancer cell lines with low metastatic potential and high endogenous miR-342 expression. Few studies have examined the consequences of miR-342 inhibition in cancer, with an early report finding that miR-342-3p inhibition restored the growth of tamoxifen-treated MCF-7 cells, although its effects in untreated cells were not assessed (Cittelly et al., 2010). MiR-342

knockout mice display context-dependent metabolic and immune related phenotypes (Fu et al, 2021; Zhang et al, 2021), whereas miR-342 appears dispensable for pancreatic acinar cell tumorigenesis (Dooley et al, 2017), suggesting that its functions are highly cell-type dependent. Our findings indicate that miR-342 is important for the proliferation of non-metastatic breast cancer cells, whereas metastatic cells appear to overcome this growth inhibitory effect of miR-342 loss through other mechanisms, potentially including activation of E2F signaling. This is consistent with the broad concept that the acquisition of metastatic potential requires multiple genetic and epigenetic alterations and suggests that loss of miR-342 alone is insufficient to confer metastatic potential. However, because miR-342 depletion impaired the growth of miR-342-high, non-metastatic cell lines, we were unable to directly assess whether miR-342 loss alone could enhance metastatic behaviour in these models, representing an important limitation of the study."

3) In line with the above changes and to improve our manuscript, we have also modified Expanded Figures 1C and 1D to include miR-342p-3p vs miR-342-5p expression data in the TCGA BRCA and METABRIC cohorts. The relative expression of miR-342-3p in the MCF-7 and MDA-MB-231 cell lines is also highlighted in Figure 2B.

Referee #2 – Response of Referee to Editor following submission of proposed amendments:

“I carefully reviewed the authors’ comments and evaluated their arguments in detail. In particular, I examined PMID: 34168137 (Figure 5), where I could not identify any qPCR data, nor is it explicitly mentioned in the manuscript. In contrast, PMID: 37099632 (Figure 2) provides a direct comparison between luminescence/fluorescence measurements and gDNA qPCR analyses. Importantly, in that study, the magnitude of the measurements obtained with both approaches was comparable, which does not appear to be the case in the manuscript currently under discussion.

I do understand the authors’ point that qPCR-based approaches have historically been used for these types of measurements. However, methodologies and technologies have evolved substantially, and newer approaches may provide improved robustness and consistency. If you believe the manuscript could still be of value to the journal, a more explicit discussion of these methodological aspects, together with the inclusion of additional references, would likely strengthen the study.

Regarding the data integration suggestions, I believe these additions would certainly be helpful.”

Response to Referee #2 with proposed amendments:

We thank the reviewer for their thoughtful assessment, which has helped to improve the overall quality of our manuscript. We believe, however, that our reference to the 2005 study may have been misunderstood. Our intention in citing this paper was simply to illustrate that qPCR-based quantification of metastatic burden is a long-established technique. It was not intended to justify the use of qPCR in place of bioluminescence imaging (BLI), which we also use in this study.

We also maintain that the use of orthogonal assays yielding concordant results strengthens, rather than weakens, our conclusions. Both BLI and qPCR are widely used methods for quantifying relative metastatic burden, and numerous studies have used both approaches within the same manuscript, in some cases directly comparing their results. Some examples include PMID: 34168137 (*Nature Communications*, Figure 5), PMID: 31521127 (*BMC Cancer*, Figure 6), and PMID: 37099632 (*Science Translational Medicine*, Figure 2).

In our study, the use of these two approaches reflects established practices in our collaborating laboratories. Specifically, in the 4T1.2 experiments, two studies were performed in the Gregory laboratory using BLI to quantify metastatic burden (Figures 1 and 2P), while a third study was conducted in the Anderson laboratory using qPCR (Figure EV2D). Importantly, both methods produced highly consistent results, demonstrating that they provide equivalent measures of metastatic burden in this setting. To clarify this point, we would appreciate the opportunity to revise the manuscript to explicitly note these

concordant findings, and to cite representative studies that have similarly employed both BLI and qPCR.

With respect to the reviewer's suggestion to streamline the manuscript by bringing the E2F signaling network more prominently into the narrative, we did perform the suggested E2F1 3'UTR reporter assay, but the results did not improve the clarity of the overall message. However, we believe that the manuscript would benefit from introducing the E2F pathway targets earlier in the Results section, before demonstrating the central role of the E2F pathway in Figure 4. We believe this would help avoid confusion and present a clearer message.

Response to Referee #2 detailing new amendments to the manuscript:

1) In line with the reviewer's suggestion, we have included an explicit discussion of of BLI and qPCR measurements, including their strengths and weaknesses, along with the inclusion of references on page 6 of the Results section (new text underlined). Furthermore, we have performed comparative BLI and qPCR analysis of the 4T1.2 tail vein experiment performed in Figure 2O-P. This analysis yielded highly concordant results, as shown in new Figure 2P, demonstrating that BLI and qPCR are both appropriate methods for assessing metastatic burden (new text on page 9 is underlined).

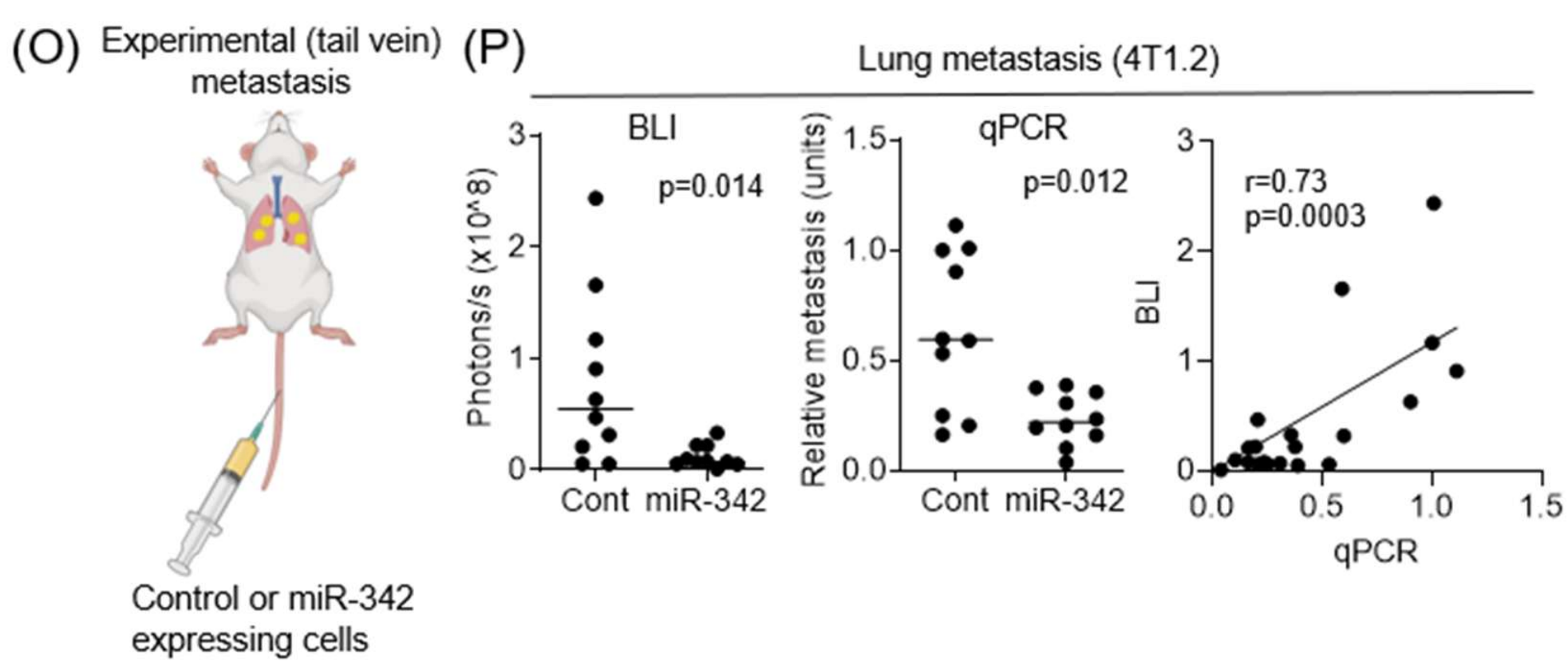

Figure 2: (P) Lung metastasis measured by ex vivo bioluminescence imaging (BLI, left panel) or by genomic DNA qPCR of murine tumour cells (mCherry) relative to all mouse cells (mouse Vimentin) in lung tissue (qPCR, middle panel) at harvest at experimental endpoint (day 13). Significance was calculated by two-tailed Mann-Whitney test (n=10). Correlation of BLI and qPCR data is shown in the right-hand panel with Pearson correlation coefficient and p-value indicated.

Page 6: "To investigate the functional role of miR-342 in TNBC metastasis, we stably overexpressed miR-342 in highly metastatic 4T1.2 cells (Figure EV2A). While increased

miR-342 expression had no effect on in vitro proliferation (Figure EV2B), it significantly suppressed tumour growth following orthotopic implantation into the mammary fat pad of Balb/c mice (Figures 1I-K). Bioluminescence imaging revealed miR-342 expression markedly reduced metastatic spread to the lungs and spine, even after normalizing for differences in primary tumour weight (Figures 1L-M). To confirm these findings, we conducted a second experiment in which tumours were harvested at matched sizes, thereby minimizing the potential confounding effect of primary tumour burden on metastatic outcome. Notably, even when allowed to grow for a longer duration, miR-342-expressing tumours displayed significantly reduced metastatic capacity (Figures EV2C-D). In this case, metastasis was measured by quantitative PCR of genomic DNA, where the amount of tumour cells are quantitated in whole lungs by the ratio of tumour specific mCherry gDNA to total gDNA levels (Chi et al, 2024; Eckhardt et al., 2005). This orthologous method has the advantage of being independent of the time and metabolism dependent bioluminescent reaction, with the potential disadvantage of reduced sensitivity, and has been used in conjunction with bioluminescence imaging in several studies (Chang et al, 2023; Samuel et al, 2021; Shekhar et al, 2019). Employing these complementary measurements, a significant reduction in lung metastasis was observed in both experiments implicating miR-342 as a potent suppressor of TNBC metastasis."

Pages 8-9: "To distinguish between impact on the initial colonisation or on outgrowth of metastases, we performed tail vein injection assays, that bypass the initial step of dissemination from the primary tumour. 231HM-i342 cells were either treated with Dox for two days prior to injection or the mice were administered Dox-containing food commencing four days after inoculation, before evaluation of lung metastasis on day 22 (Figure 2M). In both scenarios, miR-342 induction reduced metastatic outgrowth, with a larger and significant effect observed when miR-342 was activated after tumour cells had colonised the lung (Figure 23N). Similarly, stable expression of miR-342 in 4T1.2 cells markedly suppressed lung metastasis 13 days after tail vein injection, as assessed by bioluminescence imaging and confirmed by quantitative PCR of tumour-specific DNA in lung tissue, yielding highly concordant results. (Figures 2O-P). Together, these findings demonstrate that miR-342 inhibits the later stages of metastasis and most prominently metastatic outgrowth in TNBC models."

We appreciate the Referee's observation that the Results section of PMID: 34168137 does not explicitly state that the "Relative tumour burden" measurements shown in Figure 5 were obtained by qPCR. We agree that this could be clearer from the presentation of the data. However, the figure legends of both Figures 5e and 7e describe these measurements as "Relative metastatic tumour burden", and the Methods section further outlines a qPCR-based approach for quantification of metastatic burden (see "Quantification of metastatic burden"), suggesting that these

data were generated using this methodology. To clarify this point, we contacted the senior author of the study, Dr. Suresh Mathivanan, who confirmed that the measurements were indeed obtained by qPCR using the same approach described in our manuscript.

2) We have revised the section where we identify miR-342 target genes to bring identification of the E2F pathway earlier and to make it more prominent in the narrative. Changes that were made include:
    - Modifications to the text have been made on page 10-11 as below (new text underlined) with additional text changes also made in the subsequent section to maintain the narrative.

      Pages 10-11:" Since miRNAs can repress translation without necessarily reducing mRNA levels, we assessed protein changes using SILAC-based proteomics. Consistent with RNA-seq results, miR-342-3p induced modest changes in protein abundance, with several direct miR-342-3p targets noticeably downregulated at both the transcript and protein level (Figures 3G–H). <u>These included E2F1 and several E2F pathway members including RRM2, LMNB1, HMGB2, and DNMT1, as well as CDC42 and its downstream effector PAK2. Repression of selected E2F pathway members and other miR-342-3p 3'UTR targets were further validated by luciferase assays and qPCR following miR-342-3p transfection (Figures 3I–J). Together, these findings support a model in which miR-342 exerts modest repression across a broad network of target genes, with a potentially coordinated impact on the E2F pathway."</u>

    - Figure 3 has been modified to show the prominence of the E2F pathway
        - Figure 3G has E2F pathway members listed in bold (indicated in Figure legend).
        - Figure 3H has E2F1 and RRM2 at the first two Western blots shown
        - Figure 3J lists E2F1 and RRM2 first followed by other E2F pathway members and then other miR-342 target genes.

We thank the Referees for their constructive feedback and for improving the quality of our manuscript.

9th Jul 2026

Dear Prof. Gregory,

Thank you for submitting your revised manuscript following the agreed revision plan. Referees #1 and #2 have now evaluated the manuscript and, as you will see below, are satisfied with the revisions.

I will therefore be pleased to accept your manuscript once the following editorial points have been addressed:

1/ Manuscript text:
- Please accept the previous changes, and only keep in track changes any new modification.
- Methods:
o Cells: indicate the origin of the cells and whether they were authenticated.
o Animals: please indicate age and sex of the mice, include a statement of compliance with ethical regulations, and provide reference number for ethics approval. Please provide a statement on ethical endpoints and maximum tumor size.
o Statistics: please provide a paragraph on statistical analyses, and include a statement on sample size and exclusion/inclusion criteria.
- Data availability: please note that the datasets must be publicly accessible before acceptance of the manuscript.
- Remove Authors contribution and provide CRediT (Contributor Role Taxonomy) terms in the submission system instead. These replace a narrative author contribution section in the manuscript.
- Conflict of interest should be renamed "Disclosure and competing interests statement"

2/ Figures:
- Please provide size marker for Figure 2C.
- The EV tables should be uploaded as one file per table. Tables EV1-EV6 should be renamed Dataset EV1- Dataset EV6. Please add a legend to each file, in a separate tab for the 6 datasets, and to the top of the page for what will be Table EV1. The callout for Table EV7 should be updated to Table EV1.
- Please check the callouts for all figures and figure panels. There is currently a callout for a Fig. 4J, which does not exist.
- Please address the queries from our data editors in the figure legends:
1. Please define the annotated p values ****/***/**/* as well as provide the exact p-values for the same in the legend of figure 5L as appropriate.
2 Please note that the exact p values are not provided in the legends of figures 1D, H, J; 2B, 3I; 4F, G; 5C, D, G, K; EV1 E, EV3 H, EV5 D, E, H, I
3. Please indicate the statistical test used for data analysis in the legends of figures 1E, F, G; 2F, G, I, K; 3G, EV1 A
4. Please note that information related to n is missing in the legends of figures 1H, 2B, 4G
5. Please note that the error bars are not defined in the legend of figure 3I.
6. Please note that scale bar and its definition are missing for figure 2E.

3/ Checklist:
Please fill in the section Experimental study design and statistics, subsection Inclusion/exclusion criteria.

4/ Source data for figure 1F is missing.

5/ The Paper Explained should be added to the manuscript text, after the introduction.

6/ Please resize the visual abstract to 550px x 300px and make sure that the text remains legible.

7/ As part of the EMBO Publications transparent editorial process initiative (see our Editorial at http://embomolmed.embopress.org/content/2/9/329), EMBO Molecular Medicine will publish online a Review Process File (RPF) to accompany accepted manuscripts.
This file will be published in conjunction with your paper and will include the anonymous referee reports, your point-by-point response and all pertinent correspondence relating to the manuscript. Let us know whether you agree with the publication of the RPF and as here, if you want to remove or not any figures from it prior to publication.
Please note that the Authors checklist will be published at the end of the RPF.

I look forward to receiving your revised manuscript.

Yours sincerely,

Lise Roth

Lise Roth, PhD
Senior Editor
EMBO Molecular Medicine

***** Reviewer's comments *****

Referee #1 (Comments on Novelty/Model System for Author):

While the technical quality of the experiments appears to be high and the findings are novel, the potential medical impact of the findings cannot be assessed as feasibility of clinical inhibition of miR-342 has not been attempted.

Referee #1 (Remarks for Author):

The authors have sufficiently addressed the concerns I had raised.

Referee #2 (Remarks for Author):

I have revised and read the comments and modifications suggested and are in line with my review. They have carefully compared the two metastasis methodologies reporting their consistency and limitations. Also they have address the other issues, including streamline and other details. Overall the manuscript has largely improved through the revision process.

The authors addressed the remaining editorial issues.

20th Jul 2026

Dear Prof. Gregory,

Thank you for submitting your revised files. I am pleased to inform you that your manuscript is accepted for publication and is now being sent to our publisher to be included in the next available issue of EMBO Molecular Medicine.

Your manuscript will be processed for publication by EMBO Press. It will be copy edited and you will receive page proofs prior to publication. Please note that you will be contacted by Springer Nature Author Services to complete licensing and payment information.

You may qualify for financial assistance for your publication charges - either via a Springer Nature fully open access agreement or an EMBO initiative. Check your eligibility: https://link.springer.com/journal/44321/how-to-publish-with-us

Should you be planning a Press Release on your article, please notify the production team via this form in order to coordinate publication and release dates: https://forms.cloud.microsoft/e/eH4A8JVP6g

If you have any questions, please do not hesitate to contact the Editorial Office.
Thank you for your contribution to EMBO Molecular Medicine.

With kind regards,

Lise

Lise Roth, Ph.D
Senior Editor
EMBO Molecular Medicine

>>> Please note that it is EMBO Molecular Medicine policy for the transcript of the editorial process (containing referee reports and your response letter) to be published as an online supplement to each paper. If you do NOT want this, you will need to inform the Editorial Office via email immediately. More information is available here: https://link.springer.com/partners/embo-press/editorial-policies#Peer%20review

EMBO Press Author Checklist

Corresponding Author Name: Philip Gregory	
Journal Submitted to: EMBO Molecular Medicine	
Manuscript Number: EMM-2025-23012	

USEFUL LINKS FOR COMPLETING THIS FORM
The EMBO Journal - Author Guidelines
EMBO Reports - Author Guidelines
Molecular Systems Biology - Author Guidelines
EMBO Molecular Medicine - Author Guidelines

Reporting Checklist for Life Science Articles (updated January
This checklist is adapted from Materials Design Analysis Reporting (MDAR) Checklist for Authors. MDAR establishes a minimum set of requirements in transparent reporting in the life sciences (see Statement of Task: 10.31222/osf.io/9sm4x). Please follow the journal's guidelines in preparing your
Please note that a copy of this checklist will be published alongside your article.

Abridged guidelines for figures
1. Data
The data shown in figures should satisfy the following conditions:
- the data were obtained and processed according to the field's best practice and are presented to reflect the results of the experiments in an accurate and unbiased manner.
- ideally, figure panels should include only measurements that are directly comparable to each other and obtained with the same assay.
- plots include clearly labeled error bars for independent experiments and sample sizes. Unless justified, error bars should not be shown for technical
- if n<5, the individual data points from each experiment should be plotted. Any statistical test employed should be justified.
- Source Data should be included to report the data underlying figures according to the guidelines set out in the authorship guidelines on Data

2. Captions
Each figure caption should contain the following information, for each panel where they are relevant:
- a specification of the experimental system investigated (eg cell line, species name).
- the assay(s) and method(s) used to carry out the reported observations and measurements.
- an explicit mention of the biological and chemical entity(ies) that are being measured.
- an explicit mention of the biological and chemical entity(ies) that are altered/varied/perturbed in a controlled manner.
- the exact sample size (n) for each experimental group/condition, given as a number, not a range;
- a description of the sample collection allowing the reader to understand whether the samples represent technical or biological replicates (including how many animals, litters, cultures, etc.).
- a statement of how many times the experiment shown was independently replicated in the laboratory.
- definitions of statistical methods and measures:
  - common tests, such as t-test (please specify whether paired vs. unpaired), simple χ2 tests, Wilcoxon and Mann-Whitney tests, can be unambiguously identified by name only, but more complex techniques should be described in the methods section;
  - are tests one-sided or two-sided?
  - are there adjustments for multiple comparisons?
  - exact statistical test results, e.g., P values = x but not P values < x;
  - definition of 'center values' as median or average;
  - definition of error bars as s.d. or s.e.m.

Please complete ALL of the questions below.
Select "Not Applicable" only when the requested information is not relevant for your study.

Materials

Newly Created Materials	Information included in the manuscript?	In which section is the information available? (Reagents and Tools Table, Materials and Methods, Figures, Data Availability Section)
New materials and reagents need to be available; do any restrictions apply?	Not Applicable	

Antibodies	Information included in the manuscript?	In which section is the information available? (Reagents and Tools Table, Materials and Methods, Figures, Data Availability Section)
For **antibodies** provide the following information:<br>- Commercial antibodies: RRID (if possible) or supplier name, catalogue number and or/clone number<br>- Non-commercial: RRID or citation	Yes	Table EV7

DNA and RNA sequences	Information included in the manuscript?	In which section is the information available? (Reagents and Tools Table, Materials and Methods, Figures, Data Availability Section)
**Short novel DNA or RNA including primers, probes**: provide the sequences.	Yes	Table EV7

Cell materials	Information included in the manuscript?	In which section is the information available? (Reagents and Tools Table, Materials and Methods, Figures, Data Availability Section)
**Cell lines:** Provide species information, strain. Provide accession number in repository **OR** supplier name, catalog number, clone number, and/**OR** RRID.	Yes	Methods
**Primary cultures:** Provide species, strain, sex of origin, genetic modification status.	Not Applicable	
Report if the cell lines were recently **authenticated** (e.g., by STR profiling) and tested for mycoplasma contamination.	Yes	Methods

Experimental animals	Information included in the manuscript?	In which section is the information available? (Reagents and Tools Table, Materials and Methods, Figures, Data Availability Section)
**Laboratory animals or Model organisms:** Provide species, strain, sex, age, genetic modification status. Provide accession number in repository **OR** supplier name, catalog number, clone number, **OR** RRID.	Yes	Methods
**Animal observed in or captured from the field:** Provide species, sex, and age where possible.	Not Applicable	
Please detail **housing and husbandry conditions**.	Yes	Methods

Plants and microbes	Information included in the manuscript?	In which section is the information available? (Reagents and Tools Table, Materials and Methods, Figures, Data Availability Section)
**Plants:** provide species and strain, ecotype and cultivar where relevant, unique accession number if available, and source (including location for collected wild specimens).	Not Applicable	
**Microbes:** provide species and strain, unique accession number if available, and source.	Not Applicable	

Human research participants	Information included in the manuscript?	In which section is the information available? (Reagents and Tools Table, Materials and Methods, Figures, Data Availability Section)
If collected and within the bounds of privacy constraints report on age, sex and gender or ethnicity for all study participants.	Not Applicable	

Core facilities	Information included in the manuscript?	In which section is the information available? (Reagents and Tools Table, Materials and Methods, Figures, Data Availability Section)
If your work benefited from core facilities, was their service mentioned in the acknowledgments section?	Yes	Methods

Design

Study protocol	Information included in the manuscript?	In which section is the information available? (Reagents and Tools Table, Materials and Methods, Figures, Data Availability Section)
If study protocol has been **pre-registered, provide DOI in the manuscript**. For clinical trials, provide the trial registration number **OR** cite DOI.	Not Applicable	
Report the **clinical trial registration number** (at ClinicalTrials.gov or equivalent), where applicable.	Not Applicable	

Laboratory protocol	Information included in the manuscript?	In which section is the information available? (Reagents and Tools Table, Materials and Methods, Figures, Data Availability Section)
Provide DOI OR other citation details if **external detailed step-by-step protocols** are available.	Not Applicable	

Experimental study design and statistics	Information included in the manuscript?	In which section is the information available? (Reagents and Tools Table, Materials and Methods, Figures, Data Availability Section)
Include a statement about **sample size** estimate even if no statistical methods were used.	Yes	Methods
Were any steps taken to minimize the effects of subjective bias when allocating animals/samples to treatment (e.g. **randomization procedure**)? If yes, have they been described?	Yes	Methods
Include a statement about **blinding** even if no blinding was done.	Yes	Methods
Describe **inclusion/exclusion criteria** if samples or animals were excluded from the analysis. Were the criteria pre-established? If sample or data points were omitted from analysis, report if this was due to attrition or intentional exclusion and provide justification.	Yes	Methods
For every figure, are **statistical tests** justified as appropriate? Do the data meet the assumptions of the tests (e.g., normal distribution)? Describe any methods used to assess it. Is there an estimate of variation within each group of data? Is the variance similar between the groups that are being statistically compared?	Yes	Figure Legends

Sample definition and in-laboratory replication	Information included in the manuscript?	In which section is the information available? (Reagents and Tools Table, Materials and Methods, Figures, Data Availability Section)
In the figure legends: state number of times the experiment was **replicated** in laboratory.	Yes	Figure Legends
In the figure legends: define whether data describe **technical or biological replicates**.	Yes	Figure Legends

Ethics

Ethics	Information included in the manuscript?	In which section is the information available? (Reagents and Tools Table, Materials and Methods, Figures, Data Availability Section)
Studies involving **human participants**: State details of **authority granting ethics approval** (IRB or equivalent committee(s), provide reference number for approval.	Not Applicable	
Studies involving **human participants**: Include a statement confirming that **informed consent** was obtained from all subjects and that the experiments conformed to the principles set out in the WMA Declaration of Helsinki and the Department of Health and Human Services Belmont Report.	Not Applicable	
Studies involving **human participants:** For publication of **patient photos**, include a statement confirming that consent to publish was obtained.	Not Applicable	
Studies involving experimental **animals**: State details of **authority granting ethics approva**l (IRB or equivalent committee(s), provide reference number for approval. Include a statement of compliance with ethical regulations.	Yes	Methods
Studies involving **specimen and field samples:** State if relevant **permits** obtained, provide details of authority approving study; if none were required, explain why.	Not Applicable	

Dual Use Research of Concern (DURC)	Information included in the manuscript?	In which section is the information available? (Reagents and Tools Table, Materials and Methods, Figures, Data Availability Section)
Could your study fall under dual use research restrictions? Please check biosecurity documents and list of **select agents and toxins** (CDC): https://www.selectagents.gov/sat/list.htm	Not Applicable	
If you used a select agent, is the security level of the lab appropriate and reported in the manuscript?	Not Applicable	
If a study is subject to dual use research of concern regulations, is the name of the **authority granting approval and reference number** for the regulatory approval provided in the manuscript?	Not Applicable	

Reporting

The MDAR framework recommends adoption of discipline-specific guidelines, established and endorsed through community initiatives. Journals have their own policy about requiring specific guidelines and recommendations to complement MDAR.

Adherence to community standards	Information included in the manuscript?	In which section is the information available? (Reagents and Tools Table, Materials and Methods, Figures, Data Availability Section)
State if relevant guidelines or checklists (e.g., **ICMJE, MIBBI, ARRIVE, PRISMA**) have been followed or provided.	Not Applicable	
For **tumor marker prognostic studies,** we recommend that you follow the **REMARK** reporting guidelines (see link list at top right). See author guidelines, under 'Reporting Guidelines'. Please confirm you have followed these guidelines.	Not Applicable	
For **phase II and III randomized controlled trials**, please refer to the **CONSORT** flow diagram (see link list at top right) and submit the CONSORT checklist (see link list at top right) with your submission. See author guidelines, under 'Reporting Guidelines'. Please confirm you have submitted this list.	Not Applicable	

Data Availability

Data availability	Information included in the manuscript?	In which section is the information available? (Reagents and Tools Table, Materials and Methods, Figures, Data Availability Section)
Have **primary datasets** been deposited according to the journal's guidelines (see 'Data Deposition' section) and the respective accession numbers provided in the Data Availability Section?	Yes	Data Availability Section
Were **human clinical and genomic datasets** deposited in a public access-controlled repository in accordance to ethical obligations to the patients and to the applicable consent agreement?	Not Applicable	
Are **computational models** that are central and integral to a study available without restrictions in a machine-readable form? Were the relevant accession numbers or links provided?	Not Applicable	
If publicly available data were reused, provide the respective **data citations in the reference list**.	Yes	References
